# Genetic Architecture of Resistance to Stripe Rust in a Global Winter Wheat Germplasm Collection

**DOI:** 10.1534/g3.116.028407

**Published:** 2016-05-25

**Authors:** Peter Bulli, Junli Zhang, Shiaoman Chao, Xianming Chen, Michael Pumphrey

**Affiliations:** * Department of Crop and Soil Sciences, Washington State University, Pullman, Washington 99164-6420; † Department of Plant Sciences, University of California, Davis, California 95616; ‡ USDA-ARS Genotyping Laboratory, Biosciences Research Laboratory, Fargo, North Dakota 58102; § USDA-ARS, Wheat Health, Genetics and Quality Research Unit, Washington State University, Pullman, Washington 99164; ** Department of Plant Pathology, Washington State University, Pullman, Washington 99164

**Keywords:** hexaploid wheat, disease resistance, yellow rust, association mapping, QTL-tag SNP, genetics of immunity

## Abstract

Virulence shifts in populations of *Puccinia striiformis* f. sp. *tritici* (*Pst*), the causal pathogen of wheat stripe rust, are a major challenge to resistance breeding. The majority of known resistance genes are already ineffective against current races of *Pst*, necessitating the identification and introgression of new sources of resistance. Germplasm core collections that reflect the range of genetic and phenotypic diversity of crop species are ideal platforms for examining the genetic architecture of complex traits such as resistance to stripe rust. We report the results of genetic characterization and genome-wide association analysis (GWAS) for resistance to stripe rust in a core subset of 1175 accessions in the National Small Grains Collection (NSGC) winter wheat germplasm collection, based on genotyping with the wheat 9K single nucleotide polymorphism (SNP) iSelect assay and phenotyping of seedling and adult plants under natural disease epidemics in four environments. High correlations among the field data translated into high heritability values within and across locations. Population structure was evident when accessions were grouped by stripe rust reaction. GWAS identified 127 resistance loci that were effective across at least two environments, including 20 with significant genome-wide adjusted *P*-values. Based on relative map positions of previously reported genes and QTL, five of the QTL with significant genome-wide adjusted *P*-values in this study represent potentially new loci. This study provides an overview of the diversity of *Pst* resistance in the NSGC winter wheat germplasm core collection, which can be exploited for diversification of stripe rust resistance in breeding programs.

Wheat (*Triticum aestivum* L.) is the most widely cultivated cereal in the world with more than 215 million hectares planted annually (www.wheatinitiative.org). However, stripe rust (or yellow rust), caused by the basidiomycete fungus *Puccinia striiformis* Westend. f. sp. *tritici* Erikss (hereafter referred to as *Pst*), continues to pose a serious threat to global wheat production (Chen 2005, 2007; Hovmøller *et al.* 2008; Markell and Milus 2008; Milus *et al.* 2009; Wellings 2011). Controlling *Pst* through the use of foliar fungicides is less appealing in most cropping systems as it is costly, time sensitive, and complete control is impractical under severe epidemics. Therefore, improving the resistance of wheat varieties to cope with changing races of *Pst* is the preferred strategy for achieving global wheat demand. Resistance to *Pst* can be categorized as either race-specific (also referred to as all-stage resistance), which is controlled by genes with major effects, or race-nonspecific which is often expressed at the adult stages of growth (Lagudah 2011). Although cultivars with effective race-specific resistance genes are more attractive to farmers, growing cultivars with a single resistance gene often comes with a greater risk of emergence of a virulent race within a short period of time due to high selection pressure on the pathogen (Stubbs 1985; Kolmer *et al.* 2009; Jin *et al.* 2010; Wellings 2011; Hubbard *et al.* 2015). In 2000, the states of Arkansas and California suffered significant yield losses worth millions of dollars as new aggressive strains of *Pst* emerged with virulence to the race-specific resistance gene *Yr9* (Chen *et al.* 2002). These new strains of *Pst* are capable of attacking wheat growing in environments with warm temperatures that were previously considered unfavorable for development of the disease (Hovmøller *et al.* 2008; Milus *et al.* 2009). These challenges have triggered greater emphasis on the deployment of cultivars with combinations of race-nonspecific and race-specific resistance genes as a long term management strategy for *Pst* (Singh *et al.* 2000, 2005, 2011; Chen 2013; Chen *et al.* 2013; Hulbert and Pumphrey 2014). The slow-rusting resistance genes, *Yr18*, *Yr29*, and *Yr46*, and the high-temperature adult-plant (HTAP) resistance genes such as *Yr36*, *Yr39*, and *Yr52* are examples of race-nonspecific resistance genes that have been shown to provide durable resistance for over 50 years (Johnson 1984; Qayoum and Line 1985; Chen *et al.* 2013; Singh *et al.* 2014). Slow-rusting resistance is characterized by the combined effect of an increased latent period and reduced uredinial size, infection frequency, and spore production (Parlevliet 1975; Ohm and Shaner 1976; Wilcoxon 1981), and HTAP resistance is characterized by increased effectiveness with increase in temperatures and growth stage (Line and Chen 1995; Chen 2013).

There are 67 officially named stripe rust resistance genes (*Yr1-Yr67*), and 42 with temporary designations according to the 2013 Catalogue of Gene Symbols for Wheat (McIntosh *et al.* 2013) and the 2013–2014 Supplement (http://wheat.pw.usda.gov/GG2/Triticum/wgc/2013/2013-2014_Supplement.pdf). However, most of these cataloged race-specific genes are already ineffective against the group of post-2000 races of *Pst* (Chen *et al.* 2002). Frequent shifts in *Pst* populations dictate the strategy for deployment of the currently available resistance genes, and search for new sources of durable resistance. To date, over 200 loci are associated with resistance to stripe rust in wheat (Maccaferri *et al.* 2015). Most of the previous work involved in identifying these loci relied on classical linkage-mapping methods that are costly, characterized by poor resolution in QTL detection, and limit the number of alleles that can be studied simultaneously at any given locus (Flint-Garcia *et al.* 2003; Parisseaux and Bernardo 2004). Recent advances in genomic tools, including genome sequencing, high-density single nucleotide polymorphism (SNP) and genotyping by sequencing (GBS) markers, and statistical methods, have enabled the development of new approaches for mapping complex traits. Genome-wide association studies (GWAS) have emerged as the alternative approach, which maximize recent advances by exploiting cumulative recombination events that occur in the population and taking into account numerous alleles present in the population to identify significant marker-trait associations.

In wheat, GWAS has been successfully applied in mapping studies of several traits, including resistance to diseases (Gurung *et al.* 2011, 2014; Adhikari *et al.* 2011; Kollers *et al.* 2013; Bajgain *et al.* 2015; Maccaferri *et al.* 2015; Gao *et al.* 2016). Although GWAS using polyploid wheat is often characterized by poor mapping resolution, because wheat is a self-pollinating crop with a relatively short evolutionary history (Dubcovsky and Dvorak 2007), the high levels of linkage disequilibrium (LD) in wheat significantly reduces the number of markers required for finding marker-trait associations (MTAs) (Chao *et al.* 2010). While overcoming the constraints inherent to linkage mapping, GWAS introduces several other drawbacks. Population stratification, if not accounted for in GWAS, often leads to spurious associations (Flint-Garcia *et al.* 2003; Yu *et al.* 2006; Kang *et al.* 2008; Stich *et al.* 2008). Another major drawback is the limited power of GWAS to detect rare variants with individual large effects, or multi-allelic variants with minor effects (Brachi *et al.* 2011; Zhang *et al.* 2012). Thus, in order to discover robust genotype-trait associations, it is necessary to use a large population size that represents the genetic variation of a crop species, dense sets of molecular markers tagging most of the nonrepetitive genome, and mixed linear models to account for spurious associations as a result of population structure.

Germplasm core collections that reflect the genetic and phenotypic diversity of crop species are ideal platforms for association studies. The United States Department of Agriculture Agricultural Research Service National Small Grains Collection (USDA-ARS NSGC) Research Unit in Aberdeen, Idaho (http://www.ars.usda.gov/Main/docs.htm?docid=21891) maintains a collection of germplasm representing the global diversity of cereals, including wheat and its various wild relatives. The wheat germplasm core collection, constructed based on phenotypic information and passport data such as geographical origin and breeding pedigrees, is comprised of over 5000 spring and winter wheat accessions, which include cultivars, cultivated materials, breeding lines, genetic stocks, and landraces from 116 countries. Evaluation and use of the wheat accessions from the NSGC germplasm core collection for identifying genotype-trait associations through GWAS is already showing promising results. For example, the recent use of a core subset of the spring wheat germplasm collection in a GWAS revealed 97 resistance loci that are effective against the current populations of *Pst* in the western United States, and provided a detailed insight into the distribution of resistance genes and frequencies of the resistance alleles in spring wheat subpopulations around the world (Maccaferri *et al.* 2015).

The NSGC wheat germplasm core collections are currently being evaluated extensively for resistance to a variety of diseases, abiotic stresses, and agronomic and morphological traits. Here, we report a GWAS for resistance to *Pst* in a core subset of the NSGC winter wheat germplasm collection, which was conducted in order to identify *Pst* resistance loci that may be useful and accessible to wheat breeding programs throughout the world.

## Materials and Methods

### Association mapping panel

A core subset of the NSGC hexaploid winter wheat (*T. aestivum* ssp. *aestivum*) germplasm collection comprised of 1414 diverse accessions was evaluated for reactions to *Pst*. Out of the 1414 accessions, only 1221 were genotyped using the Infinium wheat SNP 9K iSelect assay. After preprocessing for missing data cutoff ≤ 10%, 1175 accessions, comprised of 174 breeding lines, 7 genetic stocks, 362 cultivars, 119 cultivated lines, and 513 landraces, were retained for the GWAS. The 1175 accessions originated from 67 countries in six continents, with Asia, Europe, North America, and South America contributing 34, 54, 6, and 3% of the accessions, respectively. Only four accessions are from both Africa and Oceania. The names of the accessions, their improvement status, and countries of origin are listed in Supplemental Material, File S1. The country-specific distribution of the accessions based on their improvement status is illustrated in Figure S1.

### Field-based evaluation of reaction to stripe rust

The accessions were evaluated in two-way crossed nonreplicated field trials as single rows (0.75 m long and 0.25 m apart) under natural disease epidemics during the 2011–2012 and 2012–2013 crop seasons at Pullman (46.744653° N, 117.11554° W) in eastern Washington State, and during the 2011–2012 and 2013–2014 crop seasons at Mount Vernon (48.439138° N, 122.387459 W) in western Washington State. Both locations are characterized by recurrence of stripe rust epidemics as a result of oversummering and overwintering of *Pst* (Sharma-Poudyal *et al.* 2014) that supply sufficient inoculum. The two sites are 500 km apart and differ in weather patterns and populations of *Pst*. The predominant local *Pst* races commonly found in the two locations during the 2011–2014 crop seasons and their virulence status are described in Table S1. The susceptible winter wheat ‘PS 279’ was planted in spreader rows bordering the nurseries to ensure sufficient inoculum for uniform disease infection. PS 279 was also used as the susceptible check every 20 rows. Reactions to stripe rust were recorded at flowering through grain filling stages of growth. Infection type (IT) was based on a 0–9 scale (Line and Qayoum 1992), with 0 = no visible sporulation; 2 = necrotic and/or chlorotic stripes with no sporulation; 3 = necrotic and/or chlorotic stripes with only a trace of sporulation; 4, 5, and 6 = necrotic and/or chlorotic stripes with light, intermediate, and moderate sporulation, respectively; and 7, 8, and 9 = abundant sporulation with necrotic and/or chlorotic stripes, chlorosis behind the sporulation area, and no chlorosis or necrosis, respectively. On the basis of the 0–9 IT scale, accessions were grouped as highly resistant to moderately resistant (IT 0–3), intermediate (IT 4–6), and moderately susceptible to highly susceptible (IT 7–9). Disease severity (SEV) was estimated visually as percentage of infected leaf area when rust severities on flag leaves of the susceptible check reached 80–100%. Early onset of the disease epidemics in the 2011–2012 crop seasons in Mount Vernon allowed us to record IT at the tillering (seedling) stage of growth.

### SNP genotyping

The method of DNA extraction was described in Maccaferri *et al.* (2015). Briefly, tissue samples were collected in 96-well coster boxes from 10-day-old seedlings, the same source of seeds used in the present study. Genomic DNA was extracted from tissues using the cetyltrimethyl ammonium bromide (CTAB) protocol (Stewart and Via 1993) at the USDA-ARS NSGC Research Unit. Chloroform:isoamyl alcohol (24:1) extraction was performed once. DNA in the aqueous phase was precipitated by adding ice-cold isopropanol, followed by washing of the DNA pellet with ice-cold 70% ethanol, and resuspension in 200 μl of TE buffer (pH 8.0).

The accessions were genotyped for 9K gene-based SNPs using the custom Infinium iSelect bead chip assay developed by the International Wheat Consortium (Cavanagh *et al.* 2013) on an iScan instrument following the manufacturer’s instructions, and the raw image data files were imported into the computer program GenomeStudio ver. 2011.11 (Illumina Inc., San Diego, CA) for SNP genotype calling. Before exporting genotype data, all calls were manually inspected to ensure call accuracy. The SNPs were ordered based on the published scaled map positions of the hexaploid wheat 9K consensus map (Cavanagh *et al.* 2013), with the chromosome arms of 4A, 5A, and 5B reoriented as described in Maccaferri *et al.* (2015).

### Quality control

For quality control, datasets were filtered on the basis of 10% threshold for missing data. To account for inflated false MTAs, SNPs with minor allele frequency (MAF) < 0.05 were excluded. These data preprocessing steps yielded 5347 polymorphic SNPs, with 94% of the retained SNPs positioned on the hexaploid wheat consensus map.

For internal control, the SNP-derived KASP (Kompetitive Allele Specific PCR) assay wMAS000003 (http://www.cerealsdb.uk.net/cerealgenomics/CerealsDB/indexNEW.php) for the slow-rusting resistance gene *Yr18/Lr34* was incorporated into the GWAS. Marker data for the slow-rusting resistance gene *Yr46/Lr67* (Forrest *et al.* 2014) was not used as an internal control because the frequency of the resistance allele was below the 5% MAF threshold. Marker screening for alleles associated with resistance gene *Yr15* and the HTAP *Yr36* resistance gene indicated that they were not present in any of the 1175 accessions based on the best available DNA markers developed by Distelfeld *et al.* (2006) and Ramirez-Gonzalez *et al.* (2015). Data for the control markers are summarized in File S2.

### Linkage disequilibrium

Information on patterns of linkage disequilibrium (LD) is required for the design of GWAS, determining the interval defining a QTL, and tracking of alleles through marker-assisted selection (MAS) (Ardlie *et al.* 2002; Kim *et al.* 2007; Mather *et al.* 2007; Khatkar *et al.* 2008; Huang *et al.* 2010). We estimated the correlation coefficient (*r*^2^) values for all pairwise comparison between intrachromosomal SNPs positioned on the 9K consensus map of hexaploid wheat (Cavanagh *et al.* 2013), and fitted a first-order natural smoothing spline regression model to visualize the LD as a function of genetic distance between markers on the same chromosome using the software JMP Genomics ver. 6.0 (SAS Institute Inc., Cary, NC). A critical value of LD *r*^2^, as evidence of linkage, was derived from distribution of the unlinked *r*^2^ following the approach of Breseghello and Sorrells (2006). The parametric 95th percentile of the unlinked *r*^2^ distribution was taken as a population-specific critical value of *r*^2^, beyond which the LD was likely to be caused by genetic linkage. The intersection of the spline curve fit to the *r*^2^ of the syntenic SNP loci with this baseline was considered as the estimate of the extent of LD in the genome.

### Population structure and genetic diversity

All 5347 SNPs were used for Fast Ward identity-by-descent clustering analysis algorithm implemented in JMP Genomics ver. 6.0. A subset of 1202 tag SNPs were used to assess population genetic structure using the model-based Bayesian clustering algorithm implemented in the computer program STRUCTURE ver. 2.3.3 (Pritchard *et al.* 2000) under the admixture model, assuming correlation allele frequencies and no informative priors, with five independent runs for each hypothetical number of subpopulations (*K*) ranging from 1–10. For each run, burn-in length of 50,000 in cycles followed by 100,000 Monte Carlo Markov Chain (MCMC) iterations was used. All other parameters were set at default. Based on the ∆*K* statistic approach (Evanno *et al.* 2005) implemented in STRUCTURE HARVESTER (Earl and vonHoldt 2012), the minimum and optimum numbers of *K* were two and three, respectively. Population structure (*Q*) matrix values from different replicates of STRUCTURE runs were analyzed by CLUMPP ver. 1.1.2 (Jakobsson and Rosenberg 2007). Individual accessions were assigned to specific population groups based on highest membership coefficient values, and population assignment was plotted using DISTRUCT ver. 1.1 (Rosenberg 2004).

To further explore the population structure, we calculated fixation index (*F_ST_*), a genetic distance measure for interpopulation differentiation compared to intrapopulation variation. All 5347 SNPs were used in the calculation with settings of *P*-value threshold = 0.05 and 10,000 permutations for testing the significance of the pairwise *F_ST_* values. We also assessed genetic diversity of the accessions in each subpopulation using PowerMarker ver. 3.25 (Liu and Muse 2005).

### Statistical analyses

All statistical analyses were performed with SAS ver. 9.3 (SAS Institute Inc., Cary, NC). To keep with standard statistical nomenclature, years from field studies were represented by environments. The residual plots revealed a non-normal distribution of the data, and thus the data were transformed based on Box-Cox power transformation, to achieve near-normality. Analyses of variance of the transformed data were performed using the SAS Mixed Procedure (Proc Mixed). Estimates of variance components were calculated by the restricted maximum likelihood method (Corbeil and Searle 1976), assuming full random model. Broad-sense heritability (*H*^2^) was calculated by ratio of estimate of genetic variance to population mean variance. A model describing the data was:

$$Y_{ijk}=\mu +l_{i}+f_{j}+lf_{ij}+e_{ij}$$

Variance of the population mean is calculated as:

$$\sigma_{y\cdot j\cdot }^{2}=\sigma_{G}^{2}+\frac{\sigma_{E}^{2}}{i}+\frac{\sigma_{\mathrm{GE}}^{2}}{i}+\frac{\sigma_{e}^{2}}{i}$$

where *i* = number of environments, *j* = population size, *l_i_* = environment variance (*σ^2^_E_*), *f_j_* = genotypic variance (*σ^2^_G_*), *lf_ij_* = genotype × environment variance (*σ^2^_GE_*), and *e_ij_* = residual variance (*σ^2^_e_*). Best linear unbiased estimates (BLUEs) were calculated across environments assuming fixed effects for genotypes. Transformed data from each environment, and BLUEs of data for each location (Mount Vernon = BLUE-MTV and Pullman = BLUE-PLM) and across environments (BLUE-ALL), were used for GWAS.

### Genome-wide association analyses

Both the model-based Bayesian and distance-based hierarchical clustering algorithms revealed a strong population structure in the panel, and thus genome-wide association mapping tests for resistance to *Pst* were performed using a mixed linear model with both kinship (*K*) and population structure (*Q*) matrices as covariates. To eliminate linear dependence between columns of the *Q* matrix, the last column was removed prior to GWAS. All analyses were conducted using the *R* package GAPIT (Lipka *et al.* 2012). Loci that are significant in at least two of the environments, with *P* ≤ 0.01 in at least one of the environments, were reported.

Our GWAS approach consisted of two steps: the first GWAS used all the 1175 accessions, and the second GWAS was performed on only 1104 accessions with IT-BLUE-ALL ranging from 3–9. The second GWAS was intended to identify loci that were either completely or partially masked by large-effect, or seedling resistance loci. Confidence intervals for the QTL identified in this study were established based on a ± 2.3 cM genetic distance corresponding to the standard critical LD *r*^2^ threshold of 0.3. Since the Benjamini-Hochberg FDR-controlling procedure provides less stringent control of Type I errors compared to familywise error rate (FWER) controlling procedures such as Bonferroni correction, we retested the individual effects of the loci with genome-wide significant associations using an analysis of variance (ANOVA) model with *Q* as a covariate. A previously developed integrated map (Maccaferri *et al.* 2015) was used to determine the relationships of the loci identified in this study with previously reported *Yr* genes and QTL. Names assigned to the QTL start with the prefix “*Q*” for QTL, followed by “*Yr*” for yellow rust, “*wsu*” for Washington State University, chromosome name, and number in cases where more than one QTL were reported per chromosome.

### Characterization of loci with significant MTAs

To saturate QTL confidence intervals of significant MTAs in this study, we projected the 90K consensus map of hexaploid wheat onto the 9K consensus map using BioMercator ver. 4.2 (Sosnowski *et al.* 2012). Full nucleotide sequences of contigs, from which the anchor and projected SNPs within the QTL confidence intervals were derived, were retrieved from the Unité de Recherche Génomique Info (URGI) (http://wheat-urgi.versailles.inra.fr/) database. The contig sequences were used for a BLASTx search against nonredundant protein database for *Brachypodium* (*Brachypodium distachyon*) and rice (*Oryza sativa* L.) in Phytozome ver. 10.3 (http://phytozome.jgi.doe.gov/pz/portal.html#!search?show=BLAST). Functional annotations of *Brachypodium* and rice proteins corresponding to the best hits were used as reference to infer the putative biological functions of the loci identified in the present study (data summarized in File S4).

### Data availability

Both the genotypic and phenotypic information for the winter wheat diversity panel presented in this study are available at the T3 Wheat database (https://triticeaetoolbox.org/wheat/). The seeds are available to scientists worldwide through the National Small Grains Collection which is a component of the National Plant Germplasm System of the United States Department of Agriculture – Agricultural Research Service (http://www.ars.usda.gov/Main/site_main.htm?modecode=20-50-05-00).

## Results

### Population structure and genetic diversity

**Figure 1:**
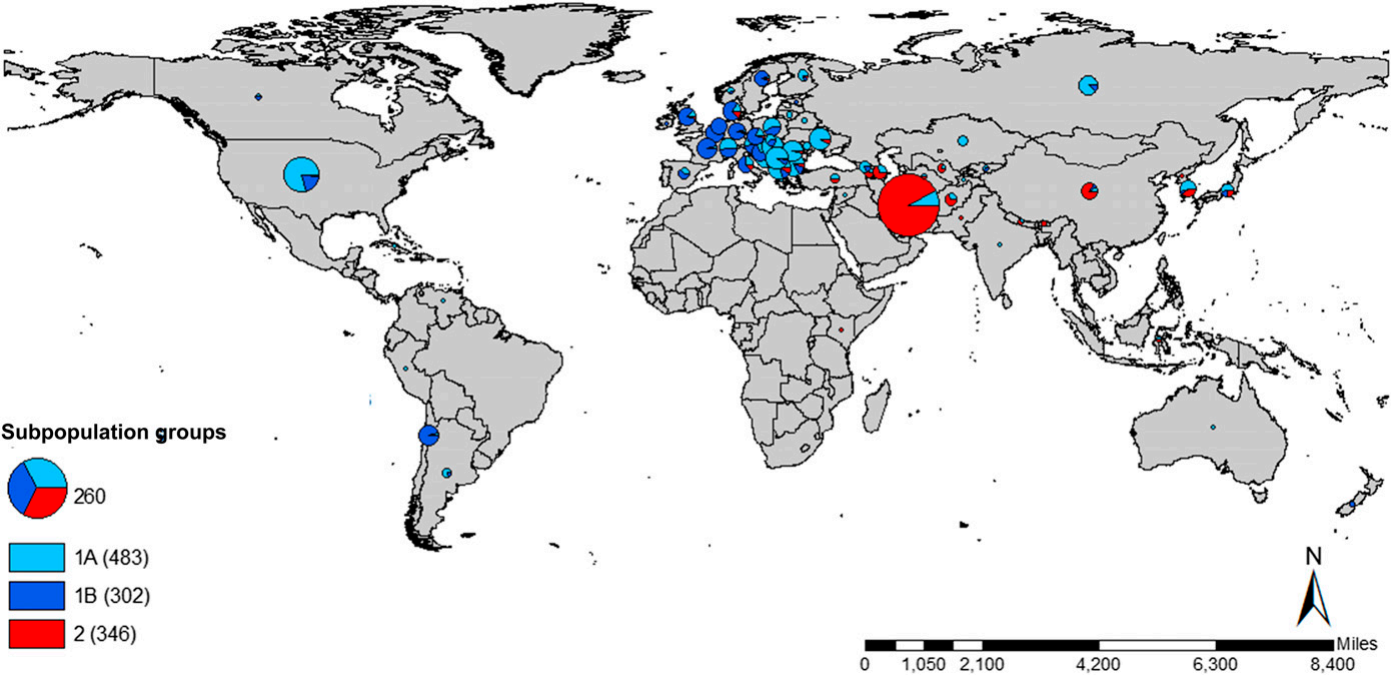
Country-specific distribution of population structure subgroups of the global winter wheat germplasm collection. Only 1131 accessions were assigned to individual countries. The 44 unassigned accessions are from the former Union of the Soviet Socialist Republic (USSR), Yugoslavia, Czechoslovakia, and other unknown parts of Europe.

**Figure 2:**
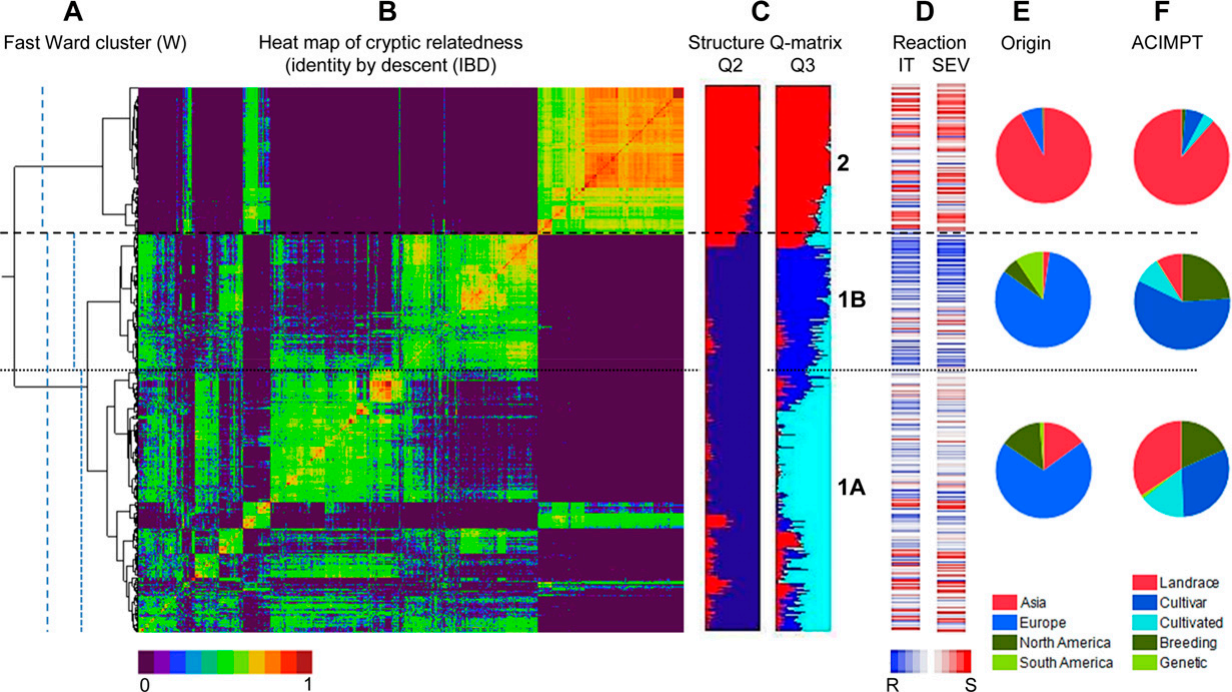
Relationship of population structure and stripe rust resistance. (A) Fast Ward clustering. Vertical dotted lines indicate genetic similarity thresholds used to classify accessions into two major groups and three subgroups. (B) Heat map of identity-by-descent (IBD) kinship matrix. The two major cluster groups and three cluster subgroups are separated by dashed and dotted lines, respectively. (C) Membership of accessions in the STRUCTURE-derived population structure subgroups. (D) Heat map of reactions of the accessions to stripe rust. IT, infection type; SEV, disease severity. Blue lines indicate resistance and red lines susceptibility based on BLUE-ALL for infection type and disease severity. (E) Frequency of population structure subgroups in geographical regions of origin. (F) Frequency of population structure subgroups in improvement status of accessions (ACIMPT).

The population genetic structure obtained from the Bayesian model-based and the Fast Ward distance-based hierarchical clustering methods revealed similar genetic variation in the accessions (Figure 2). The population of 1175 accessions was structured into two main subpopulation groups, henceforth referred to as groups 1 and 2, respectively. Group 1 further subdivides into 1A and 1B. The geographical distribution of the Bayesian model-based subpopulation groups is shown in Figure 1. European countries contributed 69% and 82% of the accessions in 1A and 1B, respectively. Cultivars and breeding lines from the US accounted for 88% of the group 1A accessions collected from the Americas, while breeding lines and landraces from Chile comprised 61% of the group 1B accessions originating from the Americas. Landraces comprised 89% of accessions in group 2, with Iran contributing 83% of the landraces.

Considerable variation in the level of admixture was evident across a large number of the accessions. Admixture among the cultivars, cultivated materials, and breeding lines in this study is consistent with our previous findings from a study on a core subset of the USDA spring wheat germplasm collection (Maccaferri *et al.* 2015). Nearly half of the landraces from Asia and Europe are classified as founders of landraces with membership probability ≥ 0.90. The majority of founders of the Asian and European landraces are from Iran, and Eastern and Southern European countries, respectively. Since information derived from population structure studies on germplasm core collections are not necessarily reflective of the range of the genetic diversity in specific countries or regions, a great deal of caution should always be exercised when interpreting the information.

### Phenotypic distribution

The IT and SEV scores for the population maxima and minima for all locations and across environments are listed in Table 1. Wide variation occurred among the accessions in all environments, with *Pst* reactions ranging from very resistant to very susceptible. On the basis of the 0–9 scale for infection type, 35.5% of the accessions were very resistant to moderately resistant, 40.3% displayed intermediate resistance, and 24.2% were moderately susceptible to very susceptible. The susceptible check PS 279 displayed very high ITs (8–9) and SEVs (80–100%) in all environments. The population showed a non-normal distribution in the field for IT-BLUE-ALL and SEV-BLUE-ALL, with frequency distributions skewed toward resistant accessions. Based on tests of power transformations, the IT and SEV data were transformed by the square root and arcsine of data to achieve near-normality.

**Table 1:** Estimates of variance components of IT and SEV of stripe rust for the global winter wheat germplasm collection

	Environment					
	MTV		PLM		Across	
	IT	SEV	IT	SEV	IT	SEV
Minimum	0	0	2	2	0	0
Maximum	9	100	9	100	9	100
$\sigma_{G}^{2}$	0.002400****	0.01022****	0.001873****	0.010050****	0.002135****	0.009748****
$\sigma_{E}^{2}$	0.000341^ns^	0.00009^ns^	0.000064^ns^	0.000165^ns^	0.000190^ns^	0.000060^ns^
$\sigma_{GE}^{2}$	0.000000	0.00000	0.000000	0.000000	0.000000	0.000000
$\sigma_{e}^{2}$	0.000919****	0.002285****	0.000594****	0.001751****	0.000751****	0.002387****
*H*^2^	0.79	0.90	0.85	0.91	0.90	0.94

Variance components were estimated from the random model using the restricted maximum likelihood method. $\sigma_{G}^{2}$, $\sigma_{E}^{2}$, $\sigma_{GE}^{2}$, and $\sigma_{e}^{2}$ represent estimates of genetic, environment, genetic × environments and residual variance components. **** *P* < 0.0001. MTV, Mount Vernon; PLM, Pullman; IT, infection type; SEV, disease severity; *H*^2^, broad-sense heritability estimate; ns, not significant.

The zero estimates of genotype × environment interactions and the high Pearson correlation coefficient (*r*) values for IT and SEV suggest stability of genotypic performance across environments. The high correlation between IT and SEV in the two locations (Table 2 and Table S2) is also a strong indication of the reliability and consistency of the data collected from the nonreplicated field trials. Taken together, these observations translated into high heritability estimates for both IT and SEV in each of the locations and across all environments.

**Table 2:** Pearson correlation coefficients among IT and SEV of stripe rust data for four environments

Trait*^a^*	MTV-IT-12	MTV-IT-14	PLM-IT-12	PLM-IT-13	MTV-SEV-12	MTV-SEV-14	PLM-SEV-12	PLM-SEV-13
MTV-IT-12	1.00	0.75	0.81	0.69	0.86	0.74	0.74	0.67
MTV-IT-14		1.00	0.77	0.76	0.82	0.94	0.79	0.75
PLM-IT-12			1.00	0.79	0.82	0.75	0.88	0.76
PLM-IT-13				1.00	0.76	0.74	0.82	0.91
MTV-SEV-12					1.00	0.84	0.85	0.77
MTV-SEV-14						1.00	0.79	0.76
PLM-SEV-12							1.00	0.85
PLM-SEV-13								1.00

All correlation coefficients are highly significant (*P* < 0.0001). MTV, Mount Vernon; IT, infection type; PLM, Pullman; SEV, disease severity.

a Trait: two locations (MTV, Mount Vernon, WA; PLM, Pullman, WA) and 3 yr (2012, 2013, and 2014).

### Influence of population structure on reaction to Pst

Population structure clearly influenced reactions to *Pst* (Figure 2D and Figure 5, A and B). Based on the heat map for IT and SEV reactions of the individual accessions (Figure 2D), we found that highly susceptible accessions had very high cluster membership in subpopulation group 2. Similarly, most of the accessions clustering in subpopulation group 2 were characterized with high IT and SEV values (Figure 5, A and B). However, for subpopulation groups 1A and 1B the opposite was true, with 1B accessions displaying a relatively high level of resistance compared to 1A accessions. Population structure (*Q*) accounted for significant proportions of the observed variance for IT-BLUE-ALL and SEV-BLUE-ALL across environments (Table S7).

Similar global trends in reaction to *Pst* were also observed among accessions based on improvement status and geographic origin. As expected, cultivars had relatively higher numbers of favorable alleles, and displayed increased resistance to *Pst* (Figure S2, A and B). We also observed a high number of favorable alleles and high levels of resistance in the accessions from Europe and South America (Figure S2, D and E). These observations are a clear demonstration of the success of modern wheat breeding practices that include resistance gene pyramiding. The relatively lower number of favorable alleles and intermediate resistance displayed by cultivars and breeding lines from the US are likely a reflection that the majority of wheat acres in the US were not historically subjected to stripe rust epidemics (Sharma-Poudyal *et al.* 2014). Not surprisingly, landraces had the lowest number of favorable alleles, and generally high levels of susceptibility.

### Linkage disequilibrium

The analysis of LD with mapped SNP markers was based on pairwise squared-allele frequency correlation (*r*^2^) for all intrachromosomal SNP loci. SNPs mapped to the 9K wheat consensus map (Cavanagh *et al.* 2013) were grouped into 28 linkage groups, covering a total map length of 1871.19 cM, with average marker density of three markers per 1.0 cM. However, marker coverage for the D genome chromosomes was poor, with an average marker density of 2.6 cM per marker.

**Figure 3:**
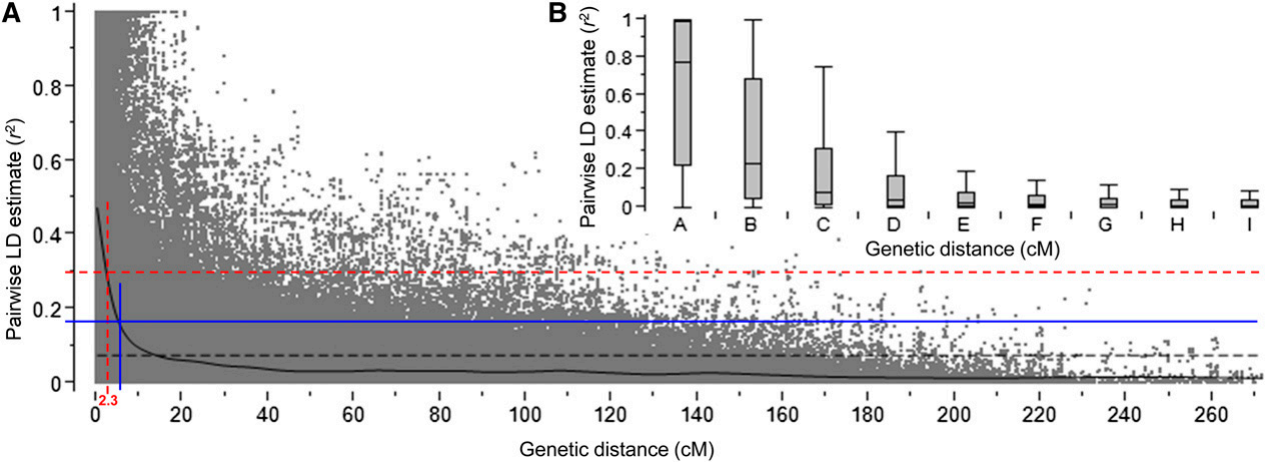
Genome-wide average linkage disequilibrium (LD) decay over genetic distance. (A) Plot of pair-wise SNP LD *r*^2^ values as a function of intermarker map cM distance based on a reference consensus map (Cavanagh *et al.* 2013). The curve represents the model fit to LD decay. The red dashed line represents the standard critical *r*^2^ = value (0.3) used for establishing QTL confidence intervals in this study; the blue solid line represents the population-specific critical *r*^2^ value (0.16) beyond which LD is likely due to linkage; and the black dashed line represents the mean. (B) Boxplot showing LD *r*^2^ values for categories of SNP pairwise map distances (A–I): 0, 0.1–1, 1.1–5, 5.1–10, 10.1–20, 20.1–30, 30.1–40, 40.1–50, and > 50 cM. SNP, single nucleotide polymorphism.

Across the 1175 accessions, the genome-wide LD generally declined with genetic distance (Figure 3, A and B). The median *r*^2^ value for pairs of completely linked markers based on the 9K wheat consensus map was 0.77, with interquartile values ranging from 0.22–0.99. At the average distance ranging from 0.1–1.0 cM, LD decayed to *r*^2^ 0.24. The median *r*^2^ decreased to 0.08 for pairs of markers within 1.1–5.0 cM genetic distance. Based on the 95th percentile of the distribution of unlinked pairwise *r*^2^ estimates across the genome, values of *r*^2^ > 0.16 (corresponding to genetic distance < 5.1 cM) were probably due to genetic linkage. QTL confidence intervals were established based on a ± intermarker genetic distance of 2.3 cM corresponding to the standard critical LD *r*^2^ of 0.3.

### Genome-wide association analyses

We compared the performance of two GWAS approaches: a general linear model (GLM) with population structure adjustment (*Q* matrix) *vs.* a mixed linear model (MLM) incorporating *K* and *Q* matrices. Distribution of observed –log_10_ *P*-values from the GLM with *Q* approach separated early from the expected distribution under a model of no association, with significant inflation of nominal *P*-values leading to a high level of false positives. On the other hand, the MLM using *K* and *Q* as covariates eliminated the moderately significant *P*-values that showed departure from the expected distribution under GLM with *Q*, and thus was selected for performing GWAS in this study. In a diverse population such as the one in this study, phenological traits are also expected to influence reactions of the plants to *Pst*. To address this, we performed GWAS on heading date and plant height data recorded in 2012 and 2013 crop seasons at the Pullman location. All SNP markers in LD with *Pst* resistance QTL in this study that were also consistently associated with heading date and/or plant height in the two environments (*P* < 0.05) were excluded from the final list of QTL-tag and -associated SNPs.

Using the 1175 accessions, we identified 100 QTL based on significant MTAs to *Pst* in at least two of the four environments (Table S3). To identify QTL that were either completely or partially masked by major effect QTL, we performed a second GWAS using only 1104 accessions with IT-BLUE-ALL ranging from 3–9, which also detected 100 QTL with significant MTAs to *Pst* (Table S4). Excluding the common QTL between the two GWAS, the total number of QTL identified in this study is 127, with 24 showing significant associations at a genome-wide adjusted significance level of *P* < 0.1 based on the False Discovery Rate (FDR) correction method (Benjamini and Hochberg 1995). However, 20 of the QTL were selected for further characterization after the effects of the significant loci (adjusted *P* < 0.1) were retested using an ANOVA model that takes population structure into account. The 20 loci (QTL-tag SNPs) are located on chromosomes 1A (*IWA5505* and *IWA3215*), 1B (*IWA5963*, *IWA5915*, and *IWA62*), 2A (*IWA2526* and *IWA5824*), 3A (*IWA3401*), 4A (*IWA3981*, *IWA3774*, *IWA6697*, *IWA4651*, and *IWA3422*), 4D (*IWA5381*), 5A (*IWA5002*), 5B (*IWA5166*), 6A (*IWA8595*), and 6B (*IWA4169*, *IWA7257*, and *IWA349*). Effects of the 20 loci and their pairwise interactions are summarized in Table 3 and Table S7.

**Table 3:** QTL significantly associated with reactions to *Puccinia striiformis* f. sp. *tritici* in at least three environments (genome-wide adjusted *P* < 0.1)

				Stages of Growth																
				Seedling	Flowering to Grain Filling															
SNP IWA*^a^*	Chr	Pos*^b^*	Allele*^c^*	IT	IT							SEV							*R*^2^ (%)	
				MTV	MTV		PLM		BLUE			MTV		PLM		BLUE			BLUE_ALL	
				2012	2012	2014	2012	2013	MTV	PLM	ALL	2012	2014	2012	2013	MTV	PLM	ALL	IT	SEV
**5505**	1A	132.0	A/G	****	****	**	****	***	****	****	****	****	**	**	**	***	**	***	4.89	4.04
**3215**	1A	182.7	A/G	ns	ns	**	**	**	**	***	**	**	**	****	***	**	****	****	2.25	2.20
5963	1B	46.0	A/C	ns	**	**	****	ns	**	**	**	*	**	**	*	**	**	**	2.66	1.60
**5915**	1B	97.1	T/C	ns	****	**	****	**	****	****	****	****	**	****	**	****	****	****	2.25	1.86
**62**	1B	NA	A/G	****	****	****	****	*	****	****	****	**	**	*	*	***	*	**	4.84	3.12
**2526**	2A	46.1	T/C	ns	*	*	*	***	*	**	**	****	**	***	***	***	****	****	3.49	4.34
**5824**	2A	72.3	A/G	ns	***	**	****	***	***	****	****	****	**	****	***	***	****	****	2.69	2.62
**3401**	3A	131.4	T/C	****	****	***	****	***	****	****	****	****	**	****	**	***	***	****	6.60	5.84
**3981**	4A	85.2	A/G	ns	****	*	*	*	**	*	**	*	*	ns	ns	*	ns	*	3.74	3.21
**3774**	4A	131.7	A/G	****	****	***	****	***	****	****	****	**	*	***	*	**	**	**	1.53	1.09
**6697**	4A	184.2	A/G	ns	*	ns	*	*	*	*	**	****	*	****	***	****	***	****	1.33	1.75
**4651**	4A	193.2	T/C	ns	**	**	**	**	**	**	***	****	***	****	**	****	****	****	1.02	1.53
**3422**	4A	198.7	T/C	ns	****	ns	****	**	**	**	**	***	ns	*	ns	**	*	**	2.62	2.32
**5381**	4D	22.4	A/G	ns	**	**	****	***	**	****	****	*	**	**	**	**	**	**	6.12	5.00
**5002**	5A	184.5	A/G	****	****	**	****	***	****	****	****	****	**	****	***	****	****	****	2.75	2.94
5166	5B	62.9	T/C	ns	*	****	**	***	***	***	***	**	****	**	***	***	***	****	1.52	1.58
8595	6A	204.5	T/C	ns	*	**	**	****	**	****	***	**	***	**	***	***	***	****	0.86	1.08
**7257**	6B	47.66	T/G	ns	***	*	****	**	**	****	****	ns	*	ns	ns	ns	ns	ns	2.14	1.20
**4169**	6B	62.22	T/G	ns	****	**	**	**	****	**	****	**	**	**	*	***	**	***	1.40	1.14
**349**	6B	126.0	T/C	ns	**	****	****	**	****	****	****	**	***	****	***	****	****	****	3.64	3.38

Marker-wise significance: * *P* < 0.05; ** *P* < 0.01; *** *P* < 0.001. Genome-wide significance: **** Benjamini-Hochberg FDR adjusted *P* < 0.10. SNP, single nucleotide polymorphisms; IWA, loci in linkage disequilibrium and with significant association with reaction to *Pst*; Chr, chromosome; Pos, position; IT, infection type; SEV, disease severity; MTV, Mount Vernon; PLM, Pullman; BLUE, best linear unbiased estimates; ALL, across environments; NA, not applicable; ns, not significant.

a SNP indexes from Illumina iSelect 9K wheat assay (Cavanagh *et al.* 2013). SNPs in **bold** were identified in the analyses using 1104 accessions with IT scores ≥ 3, and **bold and underlined** SNPs were detected in both the first and second approaches for GWAS. Loci in linkage disequilibrium and with significant association with reaction to *Pst* (IWA): 5505/475/4934, 3215/none, 5963/none, 5915/5749, 62/none, 2526/none, 5824/5495, 3401/2263/2264/2265/2266, 3981/none, 3774/none, 6697/none, 4651/none, 3422/none, 5381/none, 5002/none, 5166/none, 8595/none, 4169/none, 7257/none, and 349/none.

b Scaled positions from the hexaploid wheat 9K SNP consensus map (Cavanagh *et al.* 2013).

c Resistance allele underscored.

The genetic variance explained by the individual QTL ranged from 1.02–6.60% for IT, and 1.09–5.84% for SEV (Table 3). The cumulative genetic variances explained by the 20 QTL were 28 and 26% for IT and SEV, respectively (Table S7). When significant pairwise interactions were factored into the model, the variations increased to 37% and 33% for IT and SEV, respectively. However, the significant effects of *IWA62*, *IWA2526*, *IWA5824*, *IWA3401*, *IWA6697*, *IWA3422*, and *IWA5166* were reversed when the pairwise interactions were included in the ANOVA model. In a similar study, Maccaferri *et al.* (2015) found that the reduction or elimination of significant effects of loci involved in pairwise interaction could be attributed to either negative effects of other interacting loci or the presence of the susceptibility alleles.

Marker loci *IWA5505*, *IWA62*, *IWA3401*, *IWA3774*, and *IWA5002* also showed genome-wide significant associations with IT at the tillering (seedling) stage of growth at the Mount Vernon field site in 2012. Analysis of the field data collected at the seedling stage also revealed additional loci on 5B (*IWA7815*), 6D (*IWA93*), and 7A (*IWA7592*) that were not detected at the adult stages of growth. This can likely be explained by an increase in population of *Pst* race(s) with virulence on these genes.

Incorporation of the marker data for *Yr18/Lr34* into the GWAS as an internal control allowed us to detect significant association of *Yr18* with IT and SEV only in Mount Vernon (*P* < 0.05). In our previous study on the spring wheat core collection, *Yr18* was also associated with reactions to *Pst* in Mount Vernon but not in Pullman. We could not find linkage between *KaspLr34* and any of the SNP markers on chromosome 7D (File S2) due to inadequate marker coverage of the D genome. Accessions carrying the *Yr18* marker displayed a frequency distribution skewed toward susceptibility with 5.3, 43.4, and 51.3% of the accessions classified as resistant-moderately resistant, intermediate, and moderately susceptible-very susceptible (File S2). This is not surprising, since *Yr18* is not expected to confer adequate resistance by itself and the effect of this gene has noted environmental sensitivity (Singh and Gupta 1992; Singh *et al.* 2000).

### Influence of number of favorable alleles on reaction to Pst

**Figure 4:**
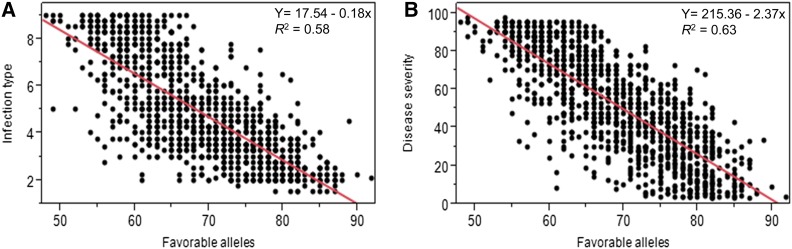
Regression of reaction to *Puccinia striiformis* f. sp. *tritici* against number of favorable alleles in each of the 1175 accessions. (A) Infection type (IT). (B) Disease severity (SEV). Both regressions were highly significant (*P* < 0.0001). Original data are available in File S1.

The total number of individual QTL (represented by QTL-tag SNPs) with significant associations with reactions to *Pst* in this present study is 127. The number of the favorable alleles in the 1175 accessions ranged from 48–92 (File S1). When accessions were ranked in descending order based on the number of favorable alleles, the top 10% with mean number of favorable alleles of 83.1 ± 0.2 showed significantly lower mean IT and SEV values of 2.6 ± 0.1 and 20.0 ± 1.2% (*P* < 0.0001) compared to the bottom 10% accessions (55.6 ± 0.2 mean number of favorable alleles), which showed higher IT and SEV values of 7.5 ± 0.1 and 82.4 ± 1.1%. Pearson correlation analyses showed a highly significant relationship (*P* < 0.0001) between the number of favorable alleles and reactions to *Pst* (*r* = −0.76 for IT-BLUE-ALL, and *r* = −0.79 for SEV-BLUE-ALL). The relatively large variation (58% for IT and 63% for SEV) explained by differences in the number of favorable alleles (Figure 4) suggests that the GWAS results in this study could be useful for analyzing the effect of genetic architecture on genomic selection (GS) prediction accuracy of resistance to wheat stripe rust, which is required for effective utilization of GS as an alternative to the traditional MAS in breeding programs (Daetwyler *et al.* 2010; Wimmer *et al.* 2013; Spindel *et al.* 2015).

### Distribution of favorable alleles for Pst resistance

The distribution of frequencies of favorable alleles of the QTL-tag SNPs based on the population structure subgroups, improvement status and geographical regions of origin are summarized in Table 4. Relatively higher frequencies of favorable alleles for *IWA8595*, *IWA4169*, and *IWA7257* were present in subpopulation group 1A. In subpopulation group 1B, we observed higher frequencies for *IWA3215*, *IWA4651*, and *IWA5166*, and very low frequencies for *IWA62*, *IWA3401*, and *IWA5002*. In subpopulation group 2, favorable alleles of *IWA5505*, *IWA62*, *IWA3401*, *IWA3774*, *IWA3422*, and *IWA349* were present at relatively higher frequencies, while *IWA5915*, *IWA2526*, and *IWA5824* were present at low frequencies. Resistance alleles were almost fixed for *IWA5963*, *IWA3981*, *IWA6697*, and *IWA5381* in all the subpopulation groups. Similar distributions were also evident when accessions were grouped by improvement status and geographical regions of origin.

**Table 4:** Frequencies of stripe rust resistance alleles

Chr	Pos*^a^*	SNP IWA*^b^*	Alleles*^c^*	Panel	Subpopulation			ACIMPT					Origin						
														Europe				Americas	
					1A	1B	2	Breeding	Genetic	Cultivar	Cultivated	Landrace	Asia	East	West	South	North	North	South
1A	132.0	**5505**	A/G	0.50	0.42	0.14	0.94	0.22	0.00	0.23	0.45	0.81	0.86	0.32	0.23	0.55	0.29	0.09	0.08
1A	182.7	**3215**	A/G	0.67	0.67	0.85	0.51	0.66	0.57	0.76	0.71	0.61	0.53	0.67	0.87	0.81	0.81	0.62	0.72
1B	46.0	5963	A/C	0.94	0.97	0.99	0.86	0.94	1.00	0.98	0.95	0.92	0.87	0.97	1.00	0.99	1.00	0.96	0.97
1B	97.1	**5915**	T/C	0.66	0.78	0.90	0.25	0.78	0.86	0.83	0.73	0.47	0.35	0.74	0.96	0.77	0.85	0.80	0.92
1B	NA	**62**	A/G	0.07	0.06	0.01	0.13	0.00	0.14	0.02	0.04	0.12	0.14	0.04	0.02	0.04	0.01	0.01	0.03
2A	46.1	**2526**	T/C	0.31	0.39	0.44	0.09	0.26	0.29	0.44	0.47	0.21	0.13	0.46	0.48	0.28	0.63	0.16	0.47
2A	72.3	**5824**	A/G	0.60	0.73	0.78	0.24	0.66	0.71	0.73	0.71	0.45	0.31	0.67	0.82	0.78	0.81	0.72	0.75
3A	131.4	**3401**	T/C	0.09	0.07	0.00	0.21	0.03	0.00	0.04	0.10	0.15	0.19	0.08	0.03	0.04	0.00	0.04	0.03
4A	85.2	**3981**	A/G	0.90	0.78	0.98	1.00	0.78	1.00	0.89	0.87	0.95	0.95	0.73	0.99	0.93	0.99	0.80	0.97
4A	131.7	**3774**	A/G	0.20	0.07	0.05	0.53	0.07	0.00	0.09	0.08	0.36	0.47	0.05	0.06	0.04	0.09	0.08	0.14
4A	184.2	**6697**	A/G	0.91	0.91	0.88	0.94	0.93	0.86	0.88	0.87	0.93	0.94	0.91	0.87	0.87	0.91	0.88	0.97
4A	193.2	**4651**	T/C	0.23	0.09	0.69	0.02	0.42	0.14	0.41	0.19	0.04	0.03	0.18	0.60	0.11	0.61	0.28	0.81
4A	198.7	**3422**	T/C	0.73	0.68	0.65	0.88	0.56	0.71	0.67	0.70	0.85	0.84	0.61	0.70	0.78	0.74	0.62	0.50
4D	22.4	**5381**	A/G	0.92	0.96	0.93	0.84	0.89	1.00	0.93	0.95	0.91	0.84	0.95	1.00	0.93	1.00	0.93	0.94
5A	184.5	**5002**	A/G	0.17	0.19	0.07	0.20	0.14	0.00	0.13	0.22	0.19	0.19	0.15	0.11	0.23	0.13	0.17	0.06
5B	62.9	5166	T/C	0.17	0.05	0.58	0.00	0.30	0.14	0.33	0.14	0.03	0.03	0.09	0.56	0.07	0.51	0.14	0.69
6A	204.5	8595	T/C	0.35	0.42	0.33	0.27	0.31	0.14	0.39	0.47	0.31	0.26	0.43	0.37	0.46	0.30	0.41	0.14
6B	47.7	7257	T/G	0.24	0.48	0.11	0.00	0.27	0.14	0.32	0.34	0.16	0.05	0.56	0.20	0.36	0.14	0.23	0.17
6B	62.2	**4169**	T/G	0.49	0.69	0.20	0.40	0.48	0.71	0.42	0.64	0.47	0.45	0.70	0.30	0.42	0.25	0.52	0.36
6B	126.0	349	T/C	0.45	0.20	0.39	0.89	0.24	0.14	0.35	0.28	0.65	0.77	0.20	0.43	0.28	0.35	0.22	0.36
Mean reaction to stripe rust																			
Infection type (0–9 scale)				5	5	4	6	5	5	4	5	5	6	5	3	5	3	6	4
Disease severity (0–100%)				52	53	32	67	52	56	41	50	59	67	50	28	49	27	60	30

Chr, chromosome; Pos, position; SNP, single nucleotide polymorphisms; IWA, loci in linkage disequilibrium and with significant association with reaction to *Pst*; ACIMPT, population structure subgroups in improvement status of accessions.

a Scaled position from hexaploid wheat consensus map (Cavanagh *et al.* 2013).

b SNP indexes from Illumina iSelect 9K wheat assay (Cavanagh *et al.* 2013). SNPs in **bold** were identified in the analyses using the 1104 accessions with IT scores ≥ 3, and **bold and underlined** SNPs were detected in both the first and second approaches for GWAS. Loci in LD and with significant association with reaction to *Pst* (IWA): 5505/475/4934, 3215/none, 5963/none, 5915/5749, 62/none, 2526/none, 5824/5495, 3401/2263/2264/2265/2266, 3981/none, 3774/none, 6697/none, 4651/none, 3422/none, 5381/none, 5002/none, 5166/none, 8595/none, 4169/none, 7257/none, 349/none.

c Resistance allele underscored.

### Comparison of Pst resistance QTL and Yr genes

Positions of QTL identified in this study relative to that of previously known *Yr* genes and QTL are visualized in Figure 6. Only 19 of the QTL in this study are positioned on the standardized chromosomes because *IWA62* is not mapped. However, *IWA62* loci are present in scaffolds from chromosomes 1A, 1B, and 1D based on TGACv1.3 genome assembly of *T. aestivum* cultivar ‘Chinese Spring’ generated by The Genome Analysis Center (TGAC), Norwich, UK. Loci identified in this study are highlighted in green, loci from our previous study on a core subset of the USDA spring wheat germplasm collection (Maccaferri *et al.* 2015) are highlighted in yellow, and common or overlapping loci between the two germplasm collections are highlighted in blue. Detailed description of Figure 6 is summarized in File S3.

Positions of SNPs tagging *IWA5505* (*QYr.wsu-1A.1*), *IWA5824* (*QYr.wsu-2A.2*), *IWA3981* (*QYr.wsu-4A.1*), *IWA3774* (*QYr.wsu-4A.2*), and *IWA349* (*QYr.wsu-6B.3*) do not correspond to that of any previously identified genes or QTL for resistance to *Pst*, and thus represent potentially novel loci. We previously reported *IWA7257* as a potential new resistance locus (Maccaferri *et al.* 2015). However, recent mapping of *IWA7257* (unpublished data) repositioned the locus in a region overlapping with previously reported APR QTL *QYr.wgp-6B.1* in the cultivar ‘Stephens’ (Santra *et al.* 2008), *QYr.sun-6B* in the Australian cultivar ‘Janz’ (Bariana *et al.* 2010), and *QYr.caas-6BS* in the Chinese landrace ‘Pingyuan 50’ (Lan *et al.* 2010). The association of *IWA7257* with resistance to *Pst* was also reported in a GWAS study on winter wheat from the Pacific Northwest region of the United States (Naruoka *et al.* 2015). Intervals where the remaining 13 QTL reside overlap with positions of either previously reported *Yr* genes or QTL for *Pst* resistance.

#### Group 1:

*IWA3215* (*QYr.wsu-1A.2*) maps within the confidence interval of *QYr.tam-1AL* identified in the hard red winter wheat ‘TAM 112’ (Basnet *et al.* 2014). However, the relationship between *IWA3215* and the QTL reported in TAM 112 could not be established because a locus with marker-wise significant associations in this study, *IWA4277*, also overlaps with *QYr.tam-1AL*. *IWA5963* (*QYr.wsu-1B.1*) maps to the gene-rich region of 1BS that includes *YrAlp*, *Yr15*, and *YrH52* (Lin and Chen 2007, 2008), and the position of *IWA5915* (*QYr.wsu-1B.2*) overlaps with the race-specific gene *YrExp1* (Lin and Chen 2008). Based on the available KASP markers R5 and R8 for *Yr15* (Ramirez-Gonzalez *et al.* 2015), the gene is not present in the 1175 winter wheat accessions. Also, *YrH52* is not expected to be present in the *T. aestivum* accessions in this study as the gene was recently identified in *T. dicoccoides* (Peng *et al.* 1999). The post-2000 races of *Pst* in the Pacific Northwest region have high virulence on both *YrAlp* and *YrExp1*, which were derived from the spring wheat cultivars ‘Alpowa’ and ‘Express,’ respectively. The position of *IWA5915* also overlaps with *IWA3017*, which showed significant marker-wise association in our previous study on the spring wheat germplasm collection (Maccaferri *et al.* 2015).

Several stripe rust resistance QTL have been mapped to the same 1BS region as *IWA5963* (Quan *et al.* 2013; Case *et al.* 2014; Naruoka *et al.* 2015). The distance between *IWA5963* and the closest QTL *QYr.wpg-1B.1* (Naruoka *et al.* 2015) is 4.71 cM, suggesting that they may represent the same QTL. *IWA5915* maps 28.33 cM proximal to the slow-rusting resistance gene *Yr29/Lr46*, and very close to the HTAP QTL *QYrex.wgp-1BL* identified in Express (Lin and Chen 2009).

#### Group 2:

*IWA2526* (*QYr.wsu-2A.1*) should be different from *Yr17* (Helguera *et al.* 2003) because *Yr17* is no longer effective against the *Pst* populations in the Pacific Northwest (Wan *et al.* 2016). The position of *IWA2526* also overlaps with the confidence intervals of QTL identified in the soft winter wheats ‘Pioneer 26R61’ (Hao *et al.* 2011) and ‘VA00W-38’ (Christopher *et al.* 2013). *IWA2526* lies 36.20 cM proximal to *QYr.ucw-2A.2*, which we recently identified in a similar study (Maccaferri *et al.* 2015). The relationship between *IWA2526* and the QTL reported in Pioneer 26R61 and VA00W-38 could not be established because a locus with marker-wise significant associations in this study, *IWA5087*, also overlaps with these QTL.

#### Group 3:

*IWA3401* (*QYr.wsu-3A*) maps in the same proximal region where Hou *et al.* (2015) identified a APR QTL, *QYrdr.wgp-3AL*, in the French winter wheat ‘Druchamp.’

#### Group 4:

*IWA6697* (*QYr.wsu-4A.3*) maps to a similar position as the previously reported *IWA1034* (*QYr.ucw-4A*) (Maccaferri *et al.* 2015), and thus are likely representing the same locus. Positions of the seedling resistance gene *Yr60* (Herrera-Foessel *et al.* 2015) and a QTL in the cultivar ‘Kariega’ (Prins *et al.* 2011) also overlap with the interval of *IWA6697*. Loci *IWA4651* (*QYr.wsu-4A.4*) and *IWA3422* (*QYr.wsu-4A.4*) reside in the 4AL/7BS translocation region where several QTL have been identified (Prins *et al.* 2011; Chen *et al.* 2012a; Vazquez *et al.* 2015). Although *IWA5381* (*QYr.wsu-4D*) on 4DS overlaps with *QYr.cim-4DS* in the CIMMYT spring wheat cultivar ‘Pastor’ (Rosewarne *et al.* 2012), we could not establish a definitive relationship between the two QTL due to overlap of a linked marker-wise significant locus *IWA2122* with *QYr.cim-4DS*.

#### Group 5:

*IWA5002* (*QYr.wsu-5A*) overlaps with positions of the APR QTL identified on the 5AL arm of the Chinese landrace Pingyuan 50 (Lan *et al.* 2010), hard red spring wheat PI 610750 (Lowe *et al.* 2011), and the spring wheat line ‘SHA3/CBRD’ (Ren *et al.* 2012). *IWA5166* (*QYr.wsu-5B*) is located within the C.I. of *QYr.cim-5BL* identified on 5BL of the CIMMYT spring wheat ‘Chapio’ (Yang *et al.* 2013). *IWA5166* is 58.87 cM proximal to the genome-wide significant seedling locus in this study, *IWA7815*, which corresponds to the race-specific gene *YrExp2* (Lin and Chen 2008).

#### Group 6:

*IWA8595* (*QYr.wsu-6A*) maps to the proximal region of 6AL, where QTL for APR have also been reported in the cultivars Stephens (Vazquez *et al.* 2012) and ‘Avocet’ (William *et al.* 2006). However, the position of *IWA7894*, which showed marker-wise significant associations with *Pst* in this study (Table S3), also overlaps with *QYrpl.orr-6AL* in Stephens (Vazquez *et al.* 2012), and thus the relationship between *IWA8595* and *QYrpl.orr-6AL* could not be established. The same region of *IWA8595* has also been associated with resistance to powdery mildew in the cultivar ‘Saar’ (Lillemo *et al.* 2008). In a RIL population derived from the rust-resistant winter wheat cultivar ‘Einstein’ and susceptible winter wheat ‘Tubbs,’ Vazquez *et al.* (2015) identified *QYrtb.orz-6AL* in a region 7.67 cM distal to *IWA8585*, suggesting the presence of more than one QTL in the proximal region of 6AL. *IWA4169* (*QYr.wsu-6B.2*) resides in the same proximal region of 6BL where a QTL for APR has been identified in the CIMMYT spring wheat ‘Pavon 76’ (William *et al.* 2006) and the French winter wheat cultivar ‘Renan’ (Dedryver *et al.* 2009). The position of *IWA4169* also overlaps with *IWA2090*, which showed marker-wise significant association with *Pst* resistance in the spring wheat germplasm collection (Maccaferri *et al.* 2015).

### Saturation of QTL regions, and comparative analyses of the genomes of wheat, Brachypodium, and rice

Enrichment of the regions of the QTL with known chromosomal locations by projecting the 90K consensus map SNPs (Wang *et al.* 2014) onto the 9K SNP consensus map (Cavanagh *et al.* 2013) increased the total number of SNPs to 979, with an average of 52 SNPs per QTL C.I. (Table S9). However, nucleotide sequences of contigs from which the SNPs were derived were available for only 490 SNPs in the wheat database of the Unité de Recherche Génomique Info, Institut National de la Recherche Agronomique in France. BLASTx searches against *Brachypodium* and rice databases assigned functional annotations to 398 of the loci, and 63 of the annotated protein domains correspond to either LRR-containing proteins or NB-ARC domains. Thirty-eight percent of these resistance-related protein domains are within the C.I. of *IWA4651* and *IWA3422*, which reside in the 4AL/7BS translocation region. Details of the 9K anchor SNPs and 90K projected SNPs, and information on transcripts corresponding to top hits with proteins in the *Brachypodium* and rice databases, are given in File S4.

## Discussion

### Population genetic structure and genetic diversity

Knowledge about genetic diversity and population structure is essential for the utilization of core collections as genetic resources for germplasm development and the diversification of the gene pool in stripe rust resistance breeding programs. In this study, the distance-based hierarchical Fast Ward clustering approach revealed two distinct major genetic groups in the population that could be explained by geography (Figure 2, A and B). The first major group mostly consisted of accessions collected from Europe and the Americas (henceforth referred to as the Western Subpopulation), and the second major group comprised primarily accessions from Asia (henceforth referred to as the Asian Subpopulation). In a study on the USDA spring wheat core collection, Maccaferri *et al.* (2015) reported the presence of two major clusters (Asian and Western) based on distance-based hierarchical clustering. Wang *et al.* (2013) also reported similar findings based on an analysis of genetic diversity in the wheat D genome. In the present study, 98% of accessions from the Americas clustered in the Western Subpopulation. Only 3% of accessions originating from Europe were found in the Asian Subpopulation. In contrast, a higher number of accessions originating from Asia (28%) were found in the Western Subpopulation. The observed pattern of distribution of the clustering-inferred groups is indicative of the path of distribution of *T*. *aestivum* following its domestication in the Fertile Crescent around 10,000 years ago (Dubcovsky and Dvorak 2007). As domesticated wheat spread from the Fertile Crescent to northwestern Europe, the crop was subjected to intensive natural selection, and enrichment of favorable alleles for key traits essential for successful adaptation in the new ecological environments.

The model-based Bayesian clustering algorithm implemented in the software STRUCTURE also revealed the presence of two distinct genetic groups that were similar to the distance-based clustering, with the ad hoc measure ∆*K* peaking at *K* = 2. However, we considered *K* = 3 as the optimum number representing genetic subgroups in the winter wheat germplasm collection as the population structures divided into well-defined subgroups, based on geographical regions of origin and improvement status (Figure 2, C, E, and F). Both subgroups 1A and 1B, which correspond to the Western Subpopulation, were dominated by accessions from Europe. The North American accessions mostly clustered in subgroup 1A, while a majority of the South American accessions were found in 1B. Accessions from the United States represent 96% of the North American accessions in 1A, and breeding lines from Chile account for 90% of the South American accessions in 1B. Subgroup 2, which corresponds to the distance-based Asian Subpopulation, is comprised mostly of landraces from Iran.

To explore the genetic diversity and relationships among and within the STRUCURE clustering-inferred groups, various diversity statistics were assessed. *F*_ST_ estimates revealed high levels of genetic differentiation between the Western Subpopulations and the Asian Subpopulation (Table S8). In contrast, minor genetic differences were observed between the 1A and 1B Western Subpopulations. The similar polymorphism information content (PIC) values of the Western Subpopulations (Table S5 and Table S6) illustrate the likely presence of a large number of accessions in both groups, with identical alleles in most of the genomic regions with low gene diversity as a result of continuous allele reshuffling and selections through modern breeding practices. This explanation is further supported by the observed considerable variation in the level of admixture in the Western Subpopulations (Figure 2C). The relatively low PIC value in the Asian Subpopulation is not surprising as it is comprised of a large number of genetically similar landraces (Figure 2B) as a result of shared ancestry, which is reflected in the high average membership coefficient value of 0.88 for accessions in the model-based subgroup 2 (Figure 2D). Compared to studies using multi-allelic markers (Roussel *et al.* 2004; Zhang *et al.* 2010; Chen *et al.* 2012b), the PIC values in this study are generally low; this is because, for biallelic markers such as SNPs, the PIC is not expected to exceed the 0.5 value for equal allele frequencies.

The variance component among the subpopulations accounted for 21.78% of the genetic variation (Table S8). Although the *F*_ST_-based differences between groups were highly significant (*P* < 0.0001), the within-subgroup component of the variance was predominant, accounting for 78.22% of the total variation. The low within-Asian Subpopulation variance component further confirms findings from the *F*_ST_ analysis that, among the defined subpopulations, the Asian subpopulation was the most distinct.

### Genome-wide association analyses

**Figure 5:**
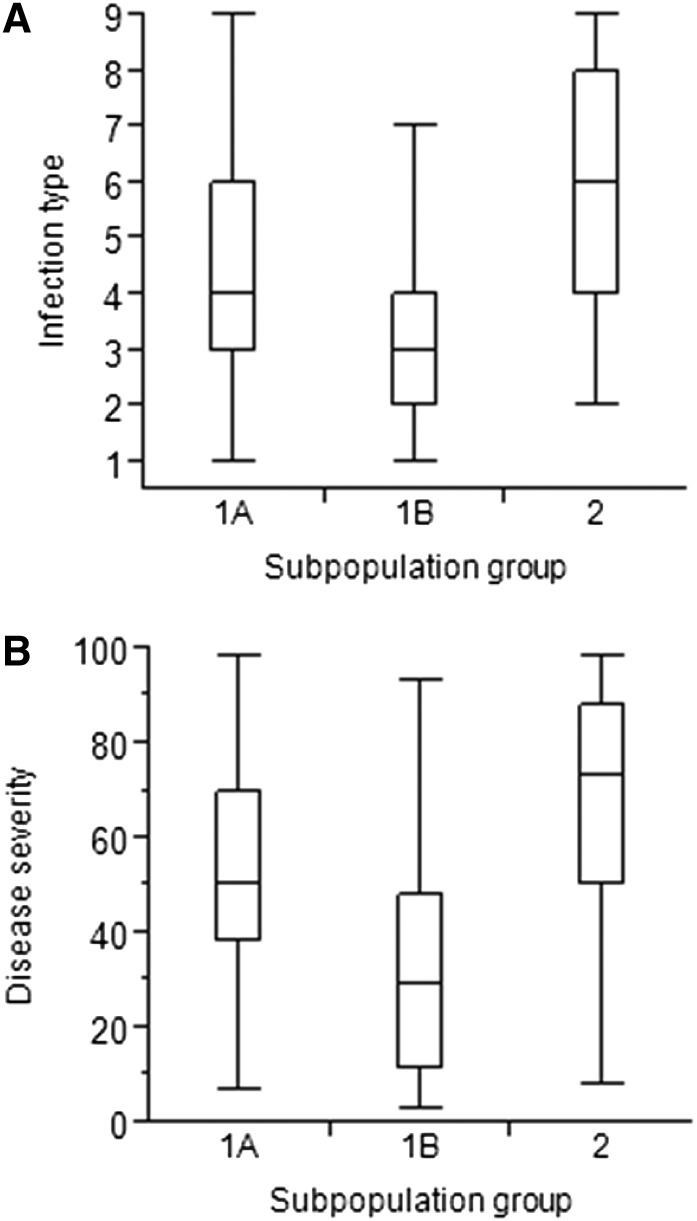
Box plot of (A) infection type (IT), and (B) disease severity (SEV) in the population structure subgroups. Horizontal lines in the boxes represent 25, 50 (median), and 75% of values; error bars include 5 and 105% of these values.

In this study, we used a mixed linear model with *K* and *Q* as covariates to account for the strong influence of population structure on *Pst* reaction (Figure 5, A and B). The two-step GWAS approach employed in this study identified a total of 127 loci on all chromosomes (except 7D) with significant associations with reactions to *Pst* in at least two environments. A more stringent approach that included FDR multiple testing correction, followed by retesting of the effects of QTL with genome-wide significant associations (adjusted *P* < 0.1) using ANOVA with population structure (*Q*) as a covariate, yielded a total of 20 loci (Table 3). Among the 20 loci, nine and 11 were detected in the GWAS using all accessions and accessions with IT-BLUE-ALL ≥ three, respectively.

To test whether the stringency of the analyses in this study created false-negatives or whether known resistance loci could not be detected due to poor marker coverage in some regions of the genome, we included marker data for the slow-rusting resistance gene complex *Yr18/Lr34/Pm38* in the GWAS. Detection of *Yr18* KASP marker-*Pst* significant associations at the Mount Vernon location (File S2) is consistent with the findings of Maccaferri *et al.* (2015), who also reported marker-wise significant associations of *Yr18* with *Pst* reactions at Mount Vernon and Davis locations. The *Yr18* KASP marker is not in LD with any of the chromosome 7D SNP markers, suggesting that the number of resistance loci in the germplasm in this study could increase with higher density of SNP markers on the array from the wheat D genome chromosomes with low LD.

The individual effects of the QTL on the observed variations ranged from 0.86–6.6%, consistent with the complex nature of the wheat-*Pst*-pathosystem, which involves numerous types of resistance genes with varying levels of effects on the pathogen. Given the heterogeneity of the germplasm collection and the likely effects of developmental genes on the observed variations in reactions to *Pst*, well-designed seedling tests are required to determine the identity of the QTL overlapping with positions of known seedling genes. The cumulative effect of the QTL with genome-wide significant associations in this study explained up to 26 and 28% of the variations for SEV and IT, respectively, which increased to 33% and 37% when significant pairwise interactions of the QTL were included in the ANOVA model (Table S7). We excluded 107 of the loci summarized in File S1 because we hypothesized that their detection is more prone to Type II error, *i.e.*, failure to reject false MTAs. However, the proportion of the variations for IT-BLUE-ALL and SEV-BLUE-ALL increased from 28% to 58% and 26% to 63%, respectively, when all 127 QTL were included in the combined ANOVA model, suggesting that the FDR multiple testing corrections in the model also resulted in Type I error, *i.e.*, rejection of true MTAs. Evidence for the presence of true MTAs among the 107 QTL is further confirmed by high correlations between reactions to *Pst* and the number of favorable alleles of all the 127 QTL, with the level of resistance increasing with increase in the number of favorable alleles (Figure 4). Similar observations were also reported by Maccaferri *et al.* (2015). To address the trade-offs between the mixed linear models accounting for population structure and familial relatedness and the general linear model with population structure adjustment, we have initiated the development of a nested association mapping population (NAM) from crosses of accessions representing the range of diversity for *Pst* resistance in the germplasm collection to a common genetic background of the susceptible Washington winter wheat breeding line, Sd1-Sd2 (Cltr 17330).

The number of QTL significantly associated with *Pst* resistance (adjusted *P* < 0.1) in this study is twice that in our previous study (Maccaferri *et al.* 2015). This could be explained by the use of a larger sample size, and the less stringent FDR multiple corrections testing to minimize the chance of any false MTAs. The post-2000 *Pst* populations in Pullman and Mount Vernon have high virulence on most of the cataloged and temporary designated *Yr* genes (Chen 2005, 2007; Wan and Chen 2014; Wan *et al.* 2016), and thus the QTL identified in this study should be useful to breeding programs that deploy combinations of both race-specific and race-nonspecific genes.

### Influence of population structure on Pst reaction

The Asian Subpopulation was generally more susceptible to *Pst* compared to the Western Subpopulations. This was not unexpected, as three-quarters of the Western Subpopulation accessions represent cultivars, cultivated lines, breeding lines, and genetic stocks that have been subjected to genome manipulation through modern breeding practices. However, differences in reaction to *Pst* have also been observed between the Western Subpopulations, with 1B exhibiting a relatively higher level of resistance. These differences could be attributed to enrichment of favorable *Pst* resistance alleles in subpopulation 1B at the six key loci *IWA3215*, *IWA5915*, *IWA2526*, *IWA5824*, *IWA4651*, and *IWA5166* (Table 4). The effect of these six key loci is even more pronounced when the accessions are grouped by geographic region of origin, with very high frequencies found in accessions from Western and Northern Europe, and South America. In contrast, very low frequencies of favorable alleles at the six loci were observed in the Asian Subpopulation, which is comprised of 89% landraces. This information can allow breeders in regions of Eastern and Southern Europe, and North America, where frequencies of subpopulation 1A are high, to develop strategies to utilize subpopulation 1B accessions as genetic resources for diversification of the stripe rust resistance gene pool in their programs.

### Extent of LD and its implications for comparative genomics

We observed a slower rate of LD decay in the winter wheat population compared to that reported by Maccaferri *et al.* (2015). This is because the winter wheat germplasm collection in this study is less diverse when compared to the spring wheat core collection, which is comprised of seven model-based subpopulation groups (Maccaferri *et al.* 2015). The high level of diversity in the spring wheat germplasm collection is not unexpected, since the crop is cultivated over a wider geographical area with different climatic conditions. Because of the relatively slower rate of LD decay, the genetic distance of ± 2.3 cM used for establishing confidence intervals for QTL in this study is also relatively higher compared to the ± 1.6 cM used in the spring wheat core collection (Maccaferri *et al.* 2015).

To enrich the regions of the QTL with additional SNP markers, we projected the 90K Illumina data (Wang *et al.* 2014) onto the 9K reference map (Cavanagh *et al.* 2013). This led to a fourfold increase in the number of SNP markers per QTL C.I., enabling collinearity analyses between the genome of wheat and the genomes of *Brachypodium* and rice (File S4). In addition to CloudBlast, the *Brachypodium* and rice protein databases in Phytozome were also used for similarity searches using sequences of wheat contigs from which the SNPs were derived. Putative functions of candidate genes in wheat were inferred based on the functional annotations of proteins of the two model species. Among the wheat SNPs with corresponding annotated *Brachypodium* or rice proteins, 16% were classified as *R* genes, with high concentration in the 4AL/7BS translocation region where *IWA4651* and *IWA3422* (Table S9) reside. Interestingly, comparative analyses between the 4AL/7BS translocation and the genomes of *Brachypodium* and rice also revealed a large number of NB-LRR and LRR-receptor-like kinases in the collinear regions of the model species. The 4AL/7BS translocation region harboring *IWA4651* and *IWA3422* showed collinearity with a 9.0–47.0 Mb region in chromosome 4 of *Brachypodium*, and a 2.0–2.7 Mb region in chromosome 11 of rice. The presence of a high number of *R* genes in collinear regions of the model species suggests that these resistance loci represent a conserved cluster of genes that evolved during ancient times, predating the event of this translocation in wheat. In a study of a segregating population derived from the durum wheat cultivars ‘Creso’ and ‘Pedroso’, Marone *et al.* (2013) identified a QTL for resistance to powdery mildew in the same region of *IWA4651*, suggesting a potential pleiotropic effect of this locus. Associations of the 4AL/7BS translocation with other adaptive traits have also been reported (Nezhad *et al.* 2012; Zanke *et al.* 2014), further confirming the selective advantage provided by this translocation during the course of evolution and the domestication of wheat crop. With the currently available dense wheat SNP and GBS markers, our understanding of the importance of this translocation will improve as more results from GWAS on other traits become publicly available.

Enrichment of the QTL confidence intervals could facilitate identification of haplotypes associated with resistance alleles, development of molecular markers, and construction of high-density genetic maps as a first step toward cloning of the defense-related candidate genes identified in this study. It is noteworthy that we could not find sequences coding for disease resistance-related proteins within the C.I. of some of the QTL in this study. This may be due, in part, to ascertainment bias where our QTL-tag SNPs are relatively far from the target loci, or the functional annotations of some of the proteins are resistance factors that are not known.

### Relative position-based comparison of Pst resistance QTL and Yr genes

**Figure 6:**
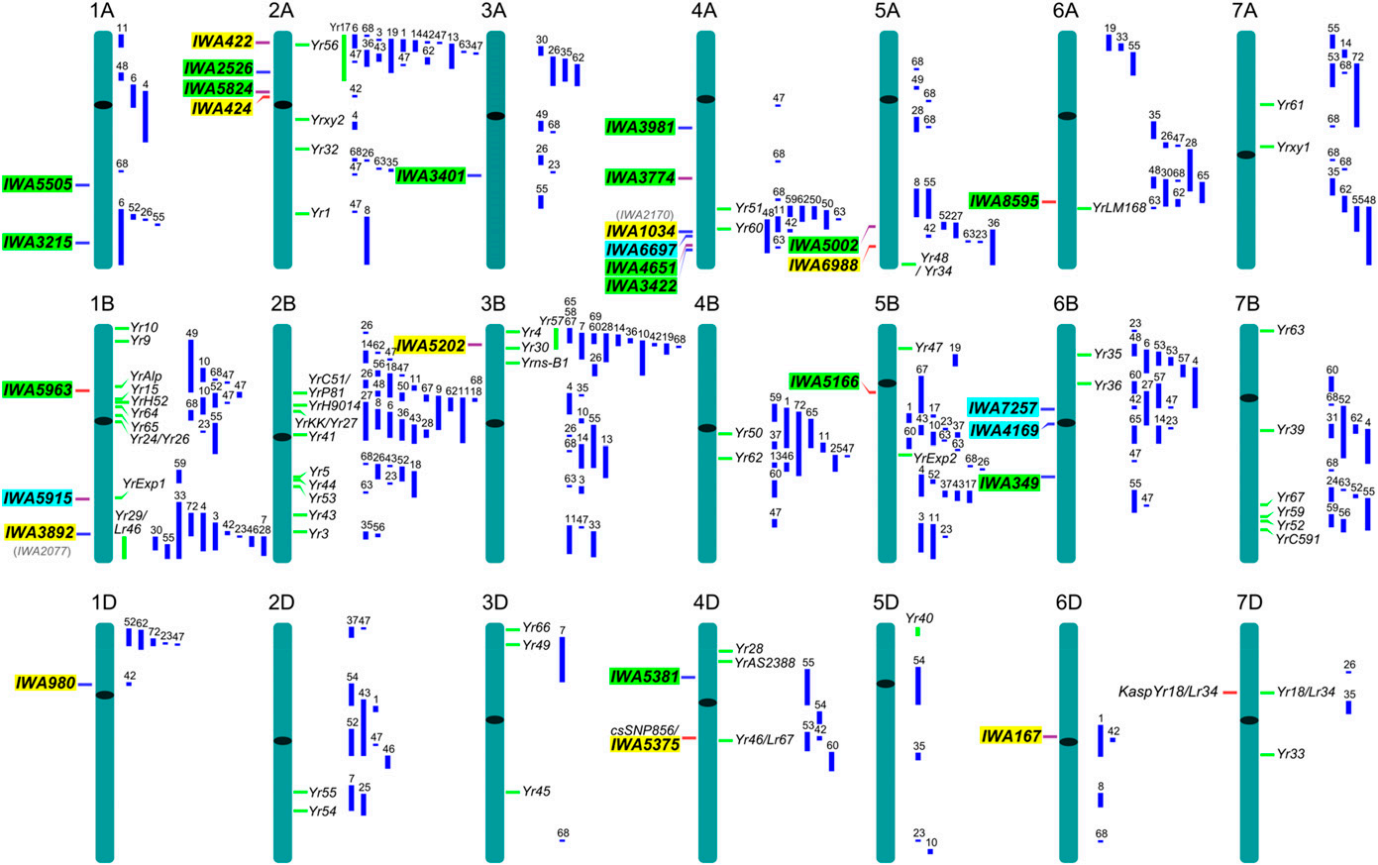
Chromosomal positions of loci associated with reactions to *Puccinia striiformis* f. sp. *tritici* identified in this study relative to positions of previously mapped *Yr* genes and QTL. All chromosomes are of standardized same relative lengths. Loci identified in this study are highlighted in green, loci from our previous study on core subset of the United States Department of Agriculture spring wheat germplasm collection (Maccaferri *et al.* 2015) are highlighted in yellow, and common or overlapping loci between the two germplasm collections are highlighted in blue. Previously mapped *Yr* genes (green bar) and QTL for stripe rust resistance (blue bar) are on right side of the chromosomes. All positions are approximations, and thus should be treated as guidelines for future studies. The relationships of the loci markers identified in this study with previously mapped *Yr* genes and QTL are described in the supporting File S3. Only loci for which the relative positions could be estimated with a precision of less than 10 cM are reported. QTL, quantitative trait loci.

Relationships of the 20 loci with genome-wide significant associations (adjusted *P* < 0.1) in this study to previously mapped QTL and *Yr* genes are summarized in Figure 6 and File S3. Although the focus in this study is on the 20 loci with genome-wide significant associations, the remaining 107 loci can be used as references for future QTL validation work. The reported positions of the QTL in this study may not be precise in some cases due to lack of sufficient common markers among the maps used for developing the integrated map. An integrated map with more accurate positions would be possible as more genetic maps constructed from SSR, DArT, SNP, and GBS markers become available.

The confidence intervals of QTL tagged by SNPs IWA5505, IWA5824, IWA3981, IWA3774, and IWA349 do not overlap with positions of any of the previously reported QTL or *Yr* genes, and thus are likely novel resistance loci. *IWA5824* and the QTL *QYr.ucw-2A.3* (*IWA424*) reported by Maccaferri *et al.* (2015) may represent two closely linked genes near the centromeric region of 2AS, since the distance between them is more than 4.6 cM. The locus *IWA3774* is present in very low frequencies in all the population structure subgroups (Table 4), providing an opportunity for exploiting this QTL for diversification of germplasm in stripe rust resistance breeding programs. *IWA62* is not in LD with any of the SNP loci on the Group 1 chromosomes, and thus we cannot estimate its map positions on 1A, 1B, and 1D. We could not establish definitive relationships between previously mapped QTL overlapping with the positions of QTL tagged by IWA3215, IWA2526, IWA5381, and IWA8595, because their confidence intervals also overlap with positions of other QTL with marker-wise significant associations in this study. Based on the integrated map developed by Maccaferri *et al.* (2015), the QTL tagged by IWA5963, IWA5915, IWA3401, IWA6697, IWA3422, IWA5002, IWA5166, IWA8595, IWA7257, IWA4169, and IWA4651 are likely corresponding to alleles of previously reported QTL (File S3).

In our previous study on a global collection of spring wheat accessions, we reported association of *IWA7257* with *Pst* resistance (Maccaferri *et al.* 2015). In that same study, we also identified a QTL *QYr.ucw-4A* tagged by IWA1034 in a similar position as *IWA6697*, suggesting these are likely alleles of the same locus. The confidence intervals of *IWA5915* and *IWA4169* also overlap with the respective positions of *IWA3017* and *IWA2090*, which were significant at marker-wise *P*-value in the spring wheat germplasm collection. Similarly, the QTL *QYr.ucw-1B* and *QYr.ucw-2A.3* with genome-wide association in our previous study also overlap with the respective positions of *IWA1791* and *IWA690*, which showed marker-wise significant associations in this study. The observed overlaps between marker-wise and experiment-wise trait associations is a clear indication that the use of multiple correction procedures to account for false positive associations in the two studies also increased the likelihood of false negatives, supporting our decision to also report the marker-wise trait associations (Table S3 and Table S4). The testing strategy we employed in both studies is further validated by the identification of 26 similar genomic regions with marker-wise trait associations on chromosomes 1A, 2A, 2B, 3A, 3B, 4A, 4B, 5B, 6A, 6B, and 7B in both the winter and spring wheat germplasm collections.

### Conclusion

The overview of the allele composition of the USDA winter wheat germplasm core collection anchored to SNP markers presented in this study can be exploited by breeders for targeted pyramiding of the alleles. Furthermore, the data in this study can be utilized for designing GWAS and genomic selection cross-validation experiments to predict performance of genotypes under high *Pst* pressure, while simultaneously analyzing the effect of all the 127 QTL on genomic selection. The identification of resistance genes as major contributing factors to the separation between the three population structure subgroups suggests the possibility of introducing the resistance alleles of the six key loci *IWA3215*, *IWA5915*, *IWA2526*, *IWA5824*, *IWA4651*, and *IWA5166* into bread varieties in regions with low frequencies of population structure subgroup 1B accessions. To assess the potential for conducting allelism tests to validate the established relationships between the QTL in this study and previously reported *Pst* resistance genes and QTL, we have initiated a study focused on introgression of individual QTL into background of the Washington *Pst*-susceptible breeding line Sd1-Sd2. However, all the QTL may not be amenable to allelism testing, since some are expected to display susceptible reactions when isolated in a genetic background without other QTL.

## Supplementary Material

Supplemental Material
