# Supplementary material for: Genetic Architecture of Resistance to Stripe Rust in a Global Winter Wheat Germplasm Collection
Source: G3 (Bethesda). 2016 May 25;6(8):2237–53. doi: 10.1534/g3.116.028407 (PMC4978880; doi:10.1534/g3.116.028407)
Supplement: Supplemental Material [file supp_6_8_2237__index.html]

Genetic Architecture of Resistance to Stripe Rust in a Global Winter Wheat Germplasm Collection — Supplemental Material 

# Genetic Architecture of Resistance to Stripe Rust in a Global Winter Wheat Germplasm Collection

## Supplemental Material for Bulli, *et al*, 2016

**Files in this Data Supplement:**

- Table S2 - Pearson correlation coefficients among the best linear unbiased estimates (BLUEs) of infection type (IT) and disease severity (SEV) for individual locations (MTV and PLM) and across locations (ALL). (.pdf, 257 KB)
- Table S1 - Predominant races of *Puccinia striiformis* f. sp. *tritici* during the 2011 to 2014 crop seasons. (.pdf, 336 KB)
- Table S3 - Loci associated with resistance to *Puccinia striiformis* f. sp. *tritici* in the global winter wheat germplasm collection in at least two environments (with marker-wise P <0.01 in at least one of the environments). (.pdf, 478 KB)
- Table S4 - Loci associated with partial resistance to *Puccinia striiformis* f. sp. *tritici* in the global winter wheat germplasm collection in at least two environments with (marker-wise P <0.01 in at least one of the environments). (.pdf, 481 KB)
- Table S5 - Frequencies of favorable alleles of QTL-tag SNPs in population structure subgroups of the global winter wheat germplasm collection. (.pdf, 360 KB)
- Table S6 - Frequencies of favorable alleles of QTL-tag SNPs associated with partial resistance in population structure subgroups of the global winter wheat germplasm collection. (.pdf, 361 KB)
- Table S7 - Analysis of variance for infection type (IT) and disease severity (SEV) using 20 quantitative trait loci (QTL) with significant genome-wide associations (adjusted P <0.1). (.pdf, 372 KB)
- Table S8 - Analysis of molecular variance (AMOVA), and FST values between pairs of subpopulations of subset of the global winter wheat core germplasm collection based on 5,347 SNPs. (.pdf, 335 KB)
- Table S9 - Summary of enrichment of genomic regions with significant marker-trait associations with reactions to *Puccinia striiformis* f. sp. *tritici*, and loci annotations. (.pdf, 364 KB)
- File S3 - Supporting information for Figure 6. (.pdf, 635 KB)
- Figure S1 - Country-specific distribution of the improvement status (ACIMPT) of the global winter wheat germplasm collection. (.pptx, 129 KB)
- Figure S2 - Box plots. (.pptx, 104 KB)
- File S1 - Genetic profiles of the 1,175 winter wheat accessions based on the 127 QTL-tag SNPs. (.xlsx, 618 KB)
- File S2 - Control resistance genes *Yr18/Lr34* and *Yr46/Lr67*. (.xlsx, 126 KB)
- File S4 - Results of Blastx search against *Brachypodium* and rice proteins using contigs of SNPs within confidence intervals of the 20 QTL with genome-wide significant association with stripe rust reaction. (.xlsx, 299 KB)
