# Supplementary material for: Genetic Architecture of Resistance to Stripe Rust in a Global Winter Wheat Germplasm Collection
Source: G3 (Bethesda). 2016 May 25;6(8):2237–53. doi: 10.1534/g3.116.028407 (PMC4978880; doi:10.1534/g3.116.028407)
Supplement: Supplemental Material [file supp_g3.116.028407_TableS2.pdf]

Table S2 Pearson correlation coefficients among the best linear unbiased estimates (BLUEs) of infection type (IT) and disease severity (SEV) for individual locations (MTV and PLM) and across locations (ALL)

Trait[a]	IT_MTV	IT_PLM	IT_ALL	SEV_MTV	SEV_PLM	SEV_ALL
IT_MTV	1.00	0.86	0.97	0.93	0.81	0.90
IT_PLM		1.00	0.96	0.85	0.92	0.92
IT_ALL			1.00	0.93	0.89	0.94
SEV_MTV				1.00	0.86	0.96
SEV_PLM					1.00	0.96
SEV_ALL						1.00

[a]Trait: IT = infection type; SEV = disease severity; MTV = Mount Vernon location; PLM = Pullman location; ALL = all environments. All correlations are significant at *P* <0.0001.
