# Supplementary material for: Genetic Architecture of Resistance to Stripe Rust in a Global Winter Wheat Germplasm Collection
Source: G3 (Bethesda). 2016 May 25;6(8):2237–53. doi: 10.1534/g3.116.028407 (PMC4978880; doi:10.1534/g3.116.028407)
Supplement: Supplemental Material [file supp_g3.116.028407_TableS1.pdf]

Table S1 Predominant races of *Puccinia striiformis* f. sp. *tritici* during the 2011 to 2014 crop seasons

Location	*Pst* Races	Virulence on genes
Pullman	PSTv-11	*Yr1*, *Yr6*, *Yr7*, *Yr8*, *Yr9*, *Yr17*, *Yr27*, *Yr43*, *Yr44*, *YrExp2*, *YrTye*
	PSTv-37	*Yr6*, *Yr7*, *Yr8*, *Yr9*, *Yr17*, *Yr27*, *Yr43*, *Yr44*, *YrTr1*, *YrExp2*
	PSTv-48	*Yr1*, *Yr6*, *Yr9*, *YrTye*
	PSTv-52	*Yr6*, *Yr7*, *Yr8*, *Yr9*, *Yr17*, *Yr27*, *Yr43*, *Yr44*, *YrExp2*
	PSTv-73	*Yr6*, *Yr7*, *Yr8*, *Yr9*, *Yr17*, *Yr27*, *Yr43*, *Yr44*, *YrExp2*, *YrTye*
	PSTv-79	*Yr1*, *Yr7*, *Yr9*, *Yr44*
Mount Vernon	PSTv-11	*Yr1*, *Yr6*, *Yr7*, *Yr8*, *Yr9*, *Yr17*, *Yr27*, *Yr43*, *Yr44*, *YrExp2*, *YrTye*
	PSTv-14	*Yr1*, *Yr6*, *Yr7*, *Yr8*, *Yr9*, *Yr17*, *Yr27*, *Yr43*, *Yr44*, *YrTr1*, *YrExp2*, *YrTye*
	PSTv-37	*Yr6*, *Yr7*, *Yr8*, *Yr9*, *Yr17*, *Yr27*, *Yr43*, *Yr44*, *YrTr1*, *YrExp2*
	PSTV-47	*Yr1*, *Yr6*, *Yr7*, *Yr8*, *Yr9*, *Yr17*, *Yr27*, *Yr43*, *Yr44*, *YrTr1*, *YrExp2*
	PSTv-52	*Yr6*, *Yr7*, *Yr8*, *Yr9*, *Yr17*, *Yr27*, *Yr43*, *Yr44*, *YrExp2*
	PSTv-71	*Yr1*, *Yr6*, *Yr7*, *Yr9*, *Yr27*, *Yr43*, *Yr44*, *YrExp2*, *YrTye*
