# Supplementary material for: Genetic Architecture of Resistance to Stripe Rust in a Global Winter Wheat Germplasm Collection
Source: G3 (Bethesda). 2016 May 25;6(8):2237–53. doi: 10.1534/g3.116.028407 (PMC4978880; doi:10.1534/g3.116.028407)
Supplement: Supplemental Material [file supp_g3.116.028407_TableS3.pdf]

Table S3 Loci associated with resistance to *Puccinia striiformis* f. sp. *tritici* in the global winter wheat germplasm collection in at least two environments (with marker-wise *P* <0.01 in at least one of the environments)

QTL-tag SNP					Associated SNP	Seedling	-log(*P*) of BLUE-IT			-log(*P*) of BLUE-SEV		
Chr[a]	Pos[b]	Index IWA[c]	Alleles[d]	Freq[e]	Index IWA	MTV-2012	MTV	PLM	ALL	MTV	PLM	ALL
1A	52.28	7715	T/C	0.11		-	1.47	**2.33**	**2.05**	**2.25**	**3.01**	**2.93**
1A	57.95	5435	A/G	0.87		-	-	1.46	-	-	1.94	1.42
1A	72.78	3666	A/G	0.56		**2.95**	**2.28**	**2.68**	**2.63**	1.90	**3.02**	**2.73**
1A	78.76	6835	A/G	0.16	1615	-	**2.14**	**2.60**	**2.63**	1.58	1.88	1.94
1A	104.02	3859	A/G	0.24	5493	1.37	1.91	**2.53**	**2.45**	**2.50**	**2.07**	**2.57**
1A	120.29	5822	T/C	0.50		-	1.32	-	-	**2.22**	-	1.50
1A	132.02	5505	A/G	0.50	475, 3284, 4934	**4.37**	**2.46**	**3.30**	**3.17**	**2.25**	1.91	**2.33**
1A	152.23	2819	A/G	0.53		**3.48**	**2.74**	**3.17**	**3.18**	**3.12**	**2.48**	**3.04**
1A	176.39	4271	T/C	0.49		-	**2.46**	**2.48**	**2.71**	1.65	-	1.63
1A	N/A	3680	A/G	0.07		1.76	**2.05**	1.38	1.81	1.39	-	-
1B	28.19	2150	T/G	0.86	8275	-	-	-	-	-	**2.11**	1.67
1B	46.00	**5963**	A/C	0.95		-	**2.33**	**2.63**	**2.76**	**2.03**	**2.28**	**2.47**
1B	63.93	5779	T/C	0.22	573	-	**2.07**	**2.70**	**2.62**	**2.34**	**2.09**	**2.41**
1B	68.72	6018	T/C	0.64		1.37	**2.03**	**2.22**	**2.38**	1.82	**2.34**	**2.38**
1B	97.13	**5915**	T/C	0.66	5749	**2.70**	**4.46**	**4.13**	**4.71**	**2.79**	**2.55**	**2.88**
1B	101.89	3097	T/C	0.68		**2.99**	**2.81**	**3.14**	**3.31**	**3.65**	**4.03**	**4.38**
1B	127.17	1791	A/C	0.84		-	**2.15**	-	1.50	-	-	-
1B	136.89	545	A/C	0.77		-	-	1.93	1.69	1.94	**2.17**	**2.22**
1B	N/A	**62**	A/G	0.07		**5.74**	**4.77**	**3.30**	**4.35**	1.88	1.52	1.84
1D	80.84	4032	T/C	0.90	7282	-	**2.29**	1.36	**2.10**	**2.36**	1.51	**2.23**
2A	4.67	6745	A/C	0.72	5424	**2.78**	**2.77**	**2.33**	**2.80**	**3.71**	1.73	**2.86**
2A	39.36	5087	A/G	0.85		-	1.73	**2.26**	**2.10**	1.62	1.98	1.97
2A	46.11	2526	T/C	0.31		**2.57**	1.80	**2.55**	**2.22**	**2.31**	**3.46**	**3.06**
2A	72.31	**5824**	A/G	0.60	5495	1.78	**3.84**	**4.94**	**4.75**	**4.15**	**4.36**	**4.67**
2A	82.27	690	T/C	0.73		-	**2.01**	1.53	1.98	**2.40**	**2.36**	**2.71**
2A	158.93	544	T/C	0.70		-	1.45	1.34	1.55	**2.07**	1.51	1.95
2B	4.98	8128	T/C	0.84		**2.09**	**2.02**	1.96	**2.12**	1.65	**2.48**	**2.27**
2B	20.46	2407	T/G	0.56		-	-	1.72	1.40	**2.40**	1.92	**2.41**
2B	112.35	6075	T/C	0.33		**2.49**	**2.10**	1.74	**2.13**	**2.33**	1.61	**2.19**
2B	121.97	4388	A/G	0.85		-	1.71	1.81	1.92	-	-	-
2B	162.84	243	A/G	0.70		-	1.31	1.95	1.74	**2.08**	**3.13**	**2.93**
2B	199.32	4096	T/C	0.17		-	1.52	**2.56**	**2.17**	-	**4.21**	**2.89**
2B	264.59	4118	A/G	0.93	3773	1.47	1.61	**2.52**	**2.20**	1.39	**2.40**	**2.11**

Table S3 continued

2B	271.78	2946	A/G	0.73		-	1.57	**2.97**	**2.39**	**2.44**	**3.24**	**3.10**
2D	116.64	5637	A/G	0.35		-	1.64	1.98	**2.12**	1.60	1.56	1.87
2D	159.88	6851	T/C	0.27		-	-	**2.55**	1.96	1.41	**3.19**	**2.58**
3A	62.49	132	T/C	0.63		1.89	**2.44**	-	1.81	-	-	-
3A	82.94	133	T/C	0.52		1.70	**3.18**	1.57	**2.60**	1.52	-	1.45
3A	100.69	5315	A/G	0.37		-	**2.04**	-	1.41	-	-	-
3A	113.34	7073	T/C	0.45		-	-	**2.24**	1.62	-	**2.62**	**2.10**
3A	135.53	3401	T/C	0.10	2263, 2264, 2265, 2266	**3.42**	**3.56**	**3.36**	**3.71**	**2.65**	**3.22**	**3.11**
3B	13.82	5106	T/C	0.20		**2.95**	1.63	1.52	1.85	**2.40**	-	1.99
3B	52.48	7342	A/G	0.51		-	-	1.37	-	-	**2.02**	1.37
3B	72.92	5677	A/G	0.83		-	1.45	**2.26**	1.94	-	**2.09**	1.83
3B	84.55	3218	T/C	0.31		-	**2.27**	**2.28**	**2.57**	1.31	1.74	1.78
3B	103.72	3601	T/C	0.86		-	1.78	**2.40**	**2.24**	-	-	-
4A	53.14	5897	T/C	0.16		-	-	1.36	1.33	**2.13**	1.90	**2.25**
4A	85.19	3981	A/G	0.90		-	**2.74**	1.60	**2.42**	1.59	-	1.56
4A	117.63	3757	T/G	0.24	3758	**2.06**	1.87	1.80	1.96	**2.30**	-	1.80
4A	131.65	**3774**	A/G	0.20		**6.12**	**4.91**	**5.27**	**5.53**	**2.52**	**2.70**	**2.85**
4A	151.29	**4527**	A/G	0.61		1.75	**2.65**	**4.05**	**3.59**	**3.45**	**4.73**	**4.57**
4A	166.59	1066	T/C	0.41	1067	1.64	**2.10**	-	1.58	1.43	-	-
4A	184.19	6697	A/G	0.93		**2.40**	1.71	-	1.39	**3.06**	**2.90**	**3.30**
4A	193.19	**4651**	T/C	0.23		**2.35**	1.97	**2.64**	**2.48**	**4.12**	**3.97**	**4.57**
4A	198.74	3422	T/C	0.74		**2.00**	1.92	**2.45**	**2.25**	**2.60**	**2.09**	**2.51**
4B	10.92	5739	T/C	0.91		-	1.62	1.48	1.74	**2.04**	1.97	**2.28**
4B	82.30	7566	T/G	0.81		-	1.42	**2.08**	1.84	-	1.33	1.40
4B	119.74	408	A/G	0.30		-	1.99	1.84	**2.12**	1.59	1.57	1.78
4D	22.36	5381	A/G	0.92		-	**2.08**	**3.16**	**2.79**	1.50	1.86	1.78
4D	52.81	2122	T/C	0.30	55, 286, 287, 2121, 3815	-	-	**2.64**	1.63	-	**2.10**	1.50
5A	36.39	8154	T/G	0.86	6287	-	1.56	-	-	**2.23**	-	-
5A	58.02	114	A/G	0.54	291, 1253, 1988	-	-	1.66	-	-	**2.73**	**2.06**
5A	64.45	5529	T/C	0.89		-	**3.27**	**2.33**	**3.19**	**2.10**	1.85	**2.25**
5A	71.10	5329	T/C	0.95		-	**2.11**	-	1.85	1.98	-	1.63
5A	107.93	5668	T/C	0.45	12, 3996	-	**2.29**	1.98	**2.32**	1.79	**2.23**	**2.18**
5A	129.60	454	T/C	0.07		-	1.82	-	-	**2.44**	-	-
5A	184.48	**5002**	A/G	0.18	5003	**3.69**	**4.03**	**4.14**	**4.52**	**5.69**	**5.34**	**6.26**
5B	12.07	4790	A/C	0.62		-	**2.15**	**2.30**	**2.41**	**2.60**	**2.28**	**2.62**

Table S3 continued

5B	32.79	4856	T/C	0.78		-	-	1.85	1.63	1.70	1.53	1.79
5B	62.90	**5166**	T/C	0.17		-	**3.53**	**3.16**	**3.80**	**3.78**	**3.07**	**3.84**
5B	151.16	4774	T/C	0.08		-	1.76	-	1.30	**3.06**	-	1.76
5B	172.48	584	T/G	0.18		-	1.33	1.58	1.64	**2.41**	**2.38**	**2.70**
5B	178.56	4954	A/G	0.58		1.60	**2.08**	**2.22**	**2.35**	**3.06**	**3.11**	**3.48**
5B	205.91	1786	A/G	0.68		-	-	1.67	-	-	**2.85**	**2.02**
5D1cult	43.91	6052	T/C	0.63		-	-	1.65	1.60	1.40	**2.68**	**2.33**
5D3cult	13.24	6190	T/C	0.53	6189	-	1.32	1.46	1.45	1.64	**2.19**	**2.07**
6A	7.84	3627	T/C	0.12	6871	-	**2.27**	1.49	1.99	**2.86**	1.72	**2.44**
6A	45.73	7286	T/C	0.81	1523	1.44	1.84	**3.20**	**2.66**	-	1.96	1.59
6A	63.61	2018	A/C	0.27	2017	-	-	**2.08**	1.46	-	1.72	1.51
6A	89.35	2458	T/C	0.34	2457, 3231, 2249, 5041, 5257, 5619	**2.45**	**2.29**	**2.84**	**2.85**	**2.05**	1.57	1.97
6A	204.49	**8595**	T/C	0.35		-	**2.30**	**3.85**	**3.21**	**3.52**	**3.71**	**3.90**
6A	210.29	7894	T/C	0.77		-	-	**2.17**	-	-	**2.05**	-
6B	27.78	1850	A/C	0.75		-	-	**2.21**	1.88	-	1.53	1.34
6B	36.68	4408	A/G	0.52	7369	-	1.64	**2.28**	**2.16**	-	-	-
6B	47.66	7257	T/G	0.25		-	1.87	**2.79**	**2.55**	-	-	-
6B	62.22	4169	T/G	0.47	4924, 5966, 4848, 6101	1.41	**2.93**	**2.52**	**2.95**	**3.11**	**2.42**	**3.04**
6B	103.69	4338	T/C	0.92	4339	1.44	**3.29**	**3.29**	**3.77**	**2.59**	**3.12**	**3.30**
6B	126.02	349	T/C	0.46		-	**2.36**	**2.54**	**2.70**	**2.61**	**2.74**	**2.92**
6B	147.91	7098	A/C	0.59		1.58	**2.30**	-	1.58	**3.35**	1.68	**2.67**
6D1	0.00	6360	T/G	0.27		-	-	1.94	-	-	-	-
6D2	64.57	4307	A/G	0.14		-	**2.50**	-	1.92	**2.87**	**2.12**	**2.68**
7A	32.82	954	A/G	0.78		-	-	-	-	1.34	**2.16**	1.99
7A	55.88	3737	A/C	0.90		-	**2.32**	1.38	**2.13**	**2.35**	1.47	**2.20**
7A	80.94	4574	T/C	0.06		**2.68**	**2.10**	1.90	**2.15**	1.98	1.92	**2.18**
7A	105.21	6868	A/G	0.61	4845, 4846, 7755, 7756	-	-	**2.53**	1.67	1.65	**2.83**	**2.45**
7A	133.84	1031	T/C	0.46	1032	**2.10**	1.35	1.93	1.69	1.47	**2.31**	**2.03**
7B	1.73	1525	T/C	0.80		-	-	1.70	-	1.50	1.96	1.89
7B	14.03	2568	A/G	0.88		-	**2.35**	-	1.85	1.69	1.72	1.88
7B	64.03	1361	A/G	0.93		-	-	-	-	**2.07**	1.64	2.00
7B	98.22	1971	T/C	0.89		**2.01**	**2.86**	**2.62**	**2.95**	**2.46**	1.55	**2.14**

[a]Chromosome
[b]Scaled position from hexaploid wheat consensus map (Cavanagh *et al*. 2013).
[c]SNP indexes from Illumina iSelect 9K wheat assay (Cavanagh *et al*. 2013). Loci in **bold** represent QTL with genome-wide significant adjusted P <0.1 in at least one environment.

[d]Underlined allele represent SNP variant associated with resistance.
[e]Frequency of favorable allele variant.
[f]SNP loci in linkage disequilibrium with QTL-tag SNP and significantly associated with reactions to *Pst*.
[g]Best linear unbiased estimates of infection type (IT) and disease severity (SEV).
[h]'-' = not significant; -log(*P*-value) 1.3, and >2 correspond to *P*-values <0.05 and ≤ 0.01, respectively; -log(*P*-values) corresponding to genome-wide adjusted *P* <0.1 are in **bold and underlined**.
Positions of QTL tagged by shaded SNPs overlap with QTL associated with resistance in spring wheat germplasm collection (Maccaferri *et al*. 2015).
