# Supplementary material for: Genetic Architecture of Resistance to Stripe Rust in a Global Winter Wheat Germplasm Collection
Source: G3 (Bethesda). 2016 May 25;6(8):2237–53. doi: 10.1534/g3.116.028407 (PMC4978880; doi:10.1534/g3.116.028407)
Supplement: Supplemental Material [file supp_g3.116.028407_TableS4.pdf]

Table S4 Loci associated with partial resistance to *Puccinia striiformis* f. sp. *tritici* in the global winter wheat germplasm collection in at least two environments with (marker-wise *P* <0.01 in at least one of the environments)

QTL-tag SNP					Associated SNP	Seedling	-log(*P*) of BLUE-IT			-log(*P*) of BLUE-SEV		
Chr	Pos	Index IWA	Alleles	Freq	Index IWA	MTV-2012	MTV	PLM	ALL	MTV	PLM	ALL
1A	52.28	7715*	T/C	0.10		-	-	**2.47**	2.00	**2.29**	**3.41**	**3.24**
1A	72.78	3666*	A/G	0.54		**2.95**	1.63	**2.27**	**2.07**	1.31	**2.09**	1.88
1A	78.76	6835*	A/G	0.16	1615	-	1.38	1.97	1.78	-	-	-
1A	104.02	3859*	A/G	0.25	5493	1.37	**2.24**	**2.81**	**2.80**	**2.89**	**2.44**	**3.03**
1A	120.29	5822*	T/C	0.52		-	-	-	-	**2.12**	-	-
1A	125.50	4523	T/C	0.63		-	**2.29**	-	1.91	1.45	1.81	1.79
1A	132.02	**5505***	A/G	0.52	475, 3284, 4934	**4.37**	**4.00**	**3.82**	**4.29**	**3.44**	**2.48**	**3.29**
1A	152.23	**2819***	A/G	0.54		**3.48**	**3.53**	**2.99**	**3.49**	**3.67**	**2.13**	**3.09**
1A	176.39	4271*	T/C	0.48		-	**2.75**	**2.98**	**3.08**	1.84	1.69	1.96
1A	182.67	**3215**	A/G	0.67		-	**2.11**	**3.03**	**2.61**	**2.61**	**3.94**	**3.51**
1A	N/A	3680*	A/G	0.07		1.76	**2.74**	**2.42**	**2.84**	**2.04**	-	1.48
1B	9.56	406	A/G	0.67		1.43	**2.08**	-	1.48	-	-	-
1B	46.00	5963*	A/C	0.94		-	**2.46**	**2.31**	**2.63**	1.78	1.97	**2.14**
1B	63.93	5779*	T/C	0.20	573	-	**2.07**	**2.31**	**2.37**	**2.40**	1.89	**2.34**
1B	68.72	6018*	T/C	0.63		1.37	1.71	1.80	1.94	1.51	1.83	1.91
1B	76.37	606	A/G	0.15		-	**2.14**	-	1.58	1.43	-	-
1B	97.13	**5749***	T/C	0.66	5749	**2.31**	**5.75**	**4.71**	**5.82**	**4.81**	**3.46**	**4.57**
1B	101.89	3097*	T/C	0.67		**2.99**	**2.00**	**2.03**	**2.19**	**2.63**	**2.95**	**3.13**
1B	136.89	545*	A/C	0.76		-	-	1.52	1.32	**2.37**	**2.13**	**2.45**
1B	N/A	**62***	A/G	0.06		**5.74**	**7.17**	**4.13**	**6.12**	**3.28**	1.73	**2.65**
2A	4.67	6745*	A/C	0.72	5424	**2.78**	**2.27**	**2.12**	**2.41**	**2.86**	1.43	**2.28**
2A	39.36	5087*	A/G	0.85		-	1.87	**2.48**	**2.33**	1.53	**2.02**	1.98
2A	46.11	**2526***	T/C	0.30		**2.57**	1.88	**2.85**	**2.46**	**3.07**	**4.05**	**3.90**
2A	72.31	**5824***	A/G	0.58	5495	1.78	**3.14**	**4.91**	**4.26**	**3.78**	**4.53**	**4.62**
2A	82.27	690*	T/C	0.72		-	1.60	-	1.45	1.73	1.66	1.88
2A	158.93	544*	T/C	0.70		-	1.78	1.88	**2.04**	**2.45**	**2.19**	**2.63**
2B	4.98	8128*	T/C	0.84		**2.09**	**2.47**	**2.74**	**2.81**	**2.68**	**3.12**	**3.24**
2B	47.95	**4285**	T/C	0.25		-	1.41	1.61	1.72	**2.64**	**2.03**	**2.71**
2B	112.35	6075*	T/C	0.31		**2.49**	1.83	**2.08**	**2.20**	**2.10**	1.74	**2.22**
2B	162.84	243*	A/G	0.69		-	1.95	**2.85**	**2.62**	**2.55**	**4.00**	**3.72**
2B	199.32	4096*	T/C	0.16		-	-	1.89	1.44	-	**3.55**	**2.00**
2B	225.51	2343	T/C	0.18		1.93	**2.48**	-	1.95	**2.02**	1.47	1.95
2B	240.34	570	A/G	0.86		-	1.80	1.68	1.96	**2.10**	-	1.60

Table S4 continued

2B	264.59	4118*	A/G	0.93	3773	1.47	**2.23**	**2.74**	**2.72**	**2.24**	**2.26**	**2.52**
2B	271.78	2946*	A/G	0.73		-	1.34	**2.24**	1.90	**2.06**	**2.27**	**2.37**
2D	159.88	6851*	T/C	0.26		-	-	1.50	-	-	**2.12**	1.53
2D	173.84	2792	T/C	0.34		1.52	1.65	1.41	1.68	**2.00**	1.70	**2.07**
3A	5.88	2993	T/C	0.84		-	-	-	-	1.44	1.55	1.63
3A	62.49	132*	T/C	0.61		1.89	**2.12**	-	1.63	1.54	-	1.58
3A	82.94	133*	T/C	0.50		1.70	**2.85**	1.35	**2.22**	1.85	-	1.63
3A	135.53	**3401***	T/C	0.09	2263, 2264, 2265, 2266	**3.42**	**4.14**	**4.05**	**4.46**	**3.19**	**3.66**	**3.72**
3B	13.82	5106*	T/C	0.20		**2.95**	1.51	1.51	1.69	**2.29**	-	1.89
3B	84.55	3218*	T/C	0.31		-	1.83	**2.02**	**2.14**	-	1.36	1.49
3B	103.72	3601*	T/C	0.85		-	1.70	**2.48**	**2.24**	1.40	-	1.50
3D3	15.21	1715	T/G	0.43	7274	1.35	1.75	**2.27**	**2.10**	1.94	**2.71**	**2.52**
4A	53.14	5897*	T/C	0.17		-	-	1.32	1.38	**2.29**	**2.03**	**2.45**
4A	61.63	4513	A/G	0.33		-	-	-	-	1.37	1.88	1.85
4A	85.19	**3981***	A/G	0.89		-	**2.98**	1.71	**2.52**	1.79	-	1.52
4A	117.63	3757*	T/G	0.25	3758	**2.06**	**2.30**	**2.72**	**2.75**	**3.21**	1.53	**2.53**
4A	131.65	**3774***	A/G	0.20		**6.12**	**3.91**	**4.49**	**4.58**	1.70	**2.14**	**2.14**
4A	166.59	1066*	T/C	0.40	1067	1.64	**2.40**	-	1.84	**2.07**	-	1.73
4A	184.19	**6697***	A/G	0.93		**2.40**	1.98	1.58	**2.00**	**3.70**	**3.61**	**4.18**
4A	193.19	**4651***	T/C	0.20		**2.35**	**2.92**	**2.84**	**3.17**	**5.36**	**3.76**	**5.12**
4A	198.74	**3422***	T/C	0.73		**2.00**	**2.60**	**2.86**	**2.89**	**2.70**	1.59	**2.24**
4A	207.06	4083	A/G	0.64		-	-	1.51	-	**2.07**	1.41	1.84
4B	68.33	4347	T/C	0.51		-	1.49	-	1.42	1.71	-	1.37
4B	119.74	408*	A/G	0.28		-	**2.28**	**2.56**	**2.66**	1.99	1.92	**2.21**
4D	22.36	**5381***	A/G	0.92		-	**2.75**	**3.79**	**3.58**	**2.32**	**2.18**	**2.46**
4D	52.81	2122*	T/C	0.27	55, 286, 287, 2121, 3815,	-	-	**2.88**	1.56	-	**2.24**	1.37
5A	12.83	7801	T/C	0.59		-	-	-	-	-	**2.20**	1.95
5A	36.39	8154*	T/G	0.86	6287	-	1.97	-	-	**2.69**	-	-
5A	58.02	114*	A/G	0.53	291, 1253, 1988	-	-	**2.36**	1.55	1.62	**3.51**	**2.79**
5A	64.45	5529*	T/C	0.89		-	**2.65**	**2.32**	**2.75**	1.96	1.72	**2.05**
5A	71.10	5329*	T/C	0.94		-	**2.12**	1.54	**2.01**	**2.17**	1.32	1.91
5A	101.10	2363	T/C	0.34		-	-	1.45	-	-	1.97	1.37
5A	107.93	5668*	T/C	0.44	12, 3996	-	1.86	**2.29**	**2.17**	1.38	**2.45**	**2.02**
5A	146.15	2959	T/G	0.42		-	-	**2.05**	1.63	1.80	**2.79**	**2.44**
5A	184.48	**5002***	A/G	0.18	5003	**3.69**	**4.13**	**4.55**	**4.77**	**4.76**	**5.62**	**5.89**

Table S4 continued

5B	0.00	868	T/C	0.79	757	-	-	**2.16**	-	-	**2.31**	1.56
5B	32.79	4856*	T/C	0.77		-	**2.04**	**2.41**	**2.44**	**2.05**	1.69	**2.12**
5B	62.90	5166*	T/C	0.15		-	1.37	1.82	1.70	**2.24**	1.60	**2.08**
5B	151.16	**4774***	T/C	0.08		-	**2.17**	-	1.86	**3.59**	-	**2.36**
5B	172.48	584*	T/G	0.18		-	-	-	-	1.50	**2.65**	**2.31**
5B	212.38	3360	T/C	0.64		-	-	**2.87**	1.97	1.54	**2.36**	**2.12**
5B	N/A	1621	T/C	0.94		-	1.39	**2.26**	1.97	-	1.54	1.56
5D3cult	13.24	6190*	T/C	0.52	6189	-	1.43	**2.17**	1.86	**2.27**	**2.86**	**2.82**
6A	7.84	3627*	T/C	0.11	6871	-	1.56	1.58	1.64	**2.80**	-	**2.11**
6A	45.73	7286*	T/C	0.81	1523	1.44	-	**2.42**	1.68	-	-	-
6A	63.61	2018*	A/C	0.27	2017	-	1.39	**3.41**	**2.46**	1.73	**2.89**	**2.52**
6A	115.76	6938	A/C	0.64	1856, 3269, 6811, 6812	**2.31**	**2.27**	1.43	**2.06**	**2.01**	-	1.53
6A	180.19	**3487**	T/C	0.80	2705	-	**3.64**	**2.84**	**3.54**	**2.05**	**2.06**	**2.28**
6A	204.49	8595*	T/C	0.34		-	**2.01**	**3.22**	**2.82**	**3.29**	**3.10**	**3.58**
6A	N/A	8617	T/C	0.08		-	1.82	**2.79**	**2.58**	1.63	**2.36**	**2.28**
6B	36.68	4408*	A/G	0.50	7369	-	1.40	**2.73**	**2.23**	-	1.59	-
6B	47.66	**7257***	T/G	0.25		-	**2.64**	**3.50**	**3.33**	-	-	-
6B	62.22	**4169***	T/G	0.49	4924, 5966, 4848, 6101	1.41	**3.83**	**2.81**	**3.58**	**3.30**	**2.52**	**3.17**
6B	103.69	4338*	T/C	0.92	4339	1.44	**2.30**	**2.45**	**2.67**	1.84	**2.33**	**2.38**
6B	126.02	**349***	T/C	0.45		-	**3.44**	**3.66**	**3.90**	**3.74**	**4.16**	**4.44**
6D1	0.00	6360*	T/G	0.28		-	-	**2.64**	1.59	-	-	-
6D2	64.57	4307*	A/G	0.12		-	1.99	1.58	1.91	**3.35**	**2.77**	**3.39**
7A	67.23	3351	T/C	0.75		-	-	1.34	-	-	1.89	1.50
7A	80.94	4574*	T/C	0.06		**2.68**	1.55	-	1.35	1.78	1.36	1.72
7A	93.50	2252	T/C	0.68		-	1.89	-	1.55	**2.46**	-	1.53
7A	105.21	6868*	A/G	0.59	4845, 4846, 7755, 7756	-	-	**2.42**	1.72	1.80	**2.38**	**2.35**
7A	133.84	1031*	T/C	0.44	1032	**2.10**	-	1.77	1.60	-	**2.08**	1.82
7B	14.03	2568*	A/G	0.87		-	**2.11**	-	1.83	1.95	1.88	**2.17**
7B	26.03	4549	T/C	0.66		-	**2.09**	-	1.68	**2.27**	-	1.53
7B	40.62	418	T/C	0.93		-	-	1.32	-	-	**2.11**	-
7B	50.22	2272	T/G	0.56		-	1.32	**2.46**	**2.03**	-	1.31	1.36
7B	98.22	1971*	T/C	0.89		**2.01**	**3.05**	**2.33**	**2.82**	**3.35**	1.72	**2.65**

[a]Chromosome
[b]Scaled position from hexaploid wheat consensus map (Cavanagh *et al*. 2013).
[c]SNP indexes from Illumina iSelect 9K wheat assay (Cavanagh *et al*. 2013). Loci in **bold** represent QTL with genome-wide significant adjusted *P* <0.1 in at least one environment.

[d]Underlined allele represent SNP variant associated with resistance.
[e]Frequency of favorable allele variant.
[f]SNP loci in linkage disequilibrium with QTL-tag SNP and significantly associated with reactions to *Pst*.
[g]Best linear unbiased estimates of infection type (IT) and disease severity (SEV).
[h]'-' = not significant; -log(*P*-value) 1.3, and >2 correspond to *P*-values <0.05 and ≤ 0.01, respectively; -log(*P*-values) corresponding to genome-wide adjusted *P* <0.1 are in **bold and underlined**.
Positions of QTL tagged by shaded SNPs overlap with QTL associated with resistance in spring wheat germplasm collection (Maccaferri *et al*. 2015).
[*]Loci identified in GWAS using entire 1,175 accessions (Table S3).
