# Supplementary material for: Genetic Architecture of Resistance to Stripe Rust in a Global Winter Wheat Germplasm Collection
Source: G3 (Bethesda). 2016 May 25;6(8):2237–53. doi: 10.1534/g3.116.028407 (PMC4978880; doi:10.1534/g3.116.028407)
Supplement: Supplemental Material [file supp_g3.116.028407_TableS5.pdf]

Table S5 Frequencies of favorable alleles of QTL-tag SNPs in population structure subgroups of the global winter wheat germplasm collection

QTL-tag SNP					Associated SNP[f]	Allele frequency		
						Population genetic structure (subgroups by Bayesian clustering)		
						1A	1B	2
Chr[a]	Pos[b]	Index IWA[c]	Alleles[d]	Freq[e]	Index IWA	(518)	(309)	(348)
1A	52.28	7715	T/C	0.11		0.11	0.22	0.00
1A	57.95	5435	A/G	0.87		0.88	0.69	0.99
1A	72.78	3666	A/G	0.56		0.31	0.90	0.62
1A	78.76	6835	A/G	0.16	1615	0.24	0.18	0.02
1A	104.02	3859	A/G	0.24	5493	0.16	0.02	0.55
1A	120.29	5822	T/C	0.50		0.39	0.15	0.99
1A	132.02	5505	A/G	0.50	475, 3284, 4934	0.42	0.14	0.95
1A	152.23	2819	A/G	0.53		0.61	0.22	0.68
1A	176.39	4271	T/C	0.49		0.51	0.73	0.26
1A	Unknown	3680	A/G	0.07		0.11	0.03	0.03
1B	28.19	2150	T/G	0.86	8275	0.81	0.92	0.87
1B	46.00	**5963**	A/C	0.95		0.97	0.99	0.86
1B	63.93	5779	T/C	0.22	573	0.22	0.39	0.05
1B	68.72	6018	T/C	0.64		0.89	0.84	0.08
1B	97.13	**5915**	T/C	0.66	5749	0.78	0.90	0.25
1B	101.89	3097	T/C	0.68		0.76	0.92	0.36
1B	127.17	1791	A/C	0.84		0.82	0.72	0.97
1B	136.89	545	A/C	0.77		0.88	0.96	0.43
1B	Unknown	**62**	A/G	0.07		0.06	0.01	0.13
1D	80.84	4032	T/C	0.90	7282	0.94	0.74	0.99
2A	4.67	6745	A/C	0.72	5424	0.73	0.67	0.73
2A	39.36	5087	A/G	0.85		0.79	0.85	0.95
2A	46.11	2526	T/C	0.31		0.39	0.44	0.09
2A	72.31	**5824**	A/G	0.60	5495	0.73	0.78	0.24
2A	82.27	690	T/C	0.73		0.95	0.97	0.20
2A	158.93	544	T/C	0.70		0.78	0.69	0.58
2B	4.98	8128	T/C	0.84		0.75	0.83	0.99
2B	20.46	2407	T/G	0.56		0.50	0.78	0.45
2B	112.35	6075	T/C	0.33		0.27	0.48	0.27
2B	121.97	4388	A/G	0.85		0.91	0.86	0.73
2B	162.84	243	A/G	0.70		0.89	0.85	0.30

Table S5 continued

2B	199.32	4096	T/C	0.17		0.13	0.36	0.05
2B	264.59	4118	A/G	0.93	3773	0.90	0.97	0.95
2B	271.78	2946	A/G	0.73		0.66	0.58	0.93
2D	116.64	5637	A/G	0.35		0.42	0.61	0.01
2D	159.88	6851	T/C	0.27		0.34	0.42	0.03
**3A**	**62.49**	**132**	**T/C**	**0.63**		**0.87**	**0.72**	**0.18**
3A	82.94	133	T/C	0.52		0.65	0.67	0.18
3A	100.69	5315	A/G	0.37		0.25	0.86	0.12
3A	113.34	7073	T/C	0.45		0.65	0.60	0.02
3A	135.53	3401	T/C	0.10	2263, 2264, 2265, 2266	0.07	0.00	0.21
3B	13.82	5106	T/C	0.20		0.08	0.16	0.41
3B	52.48	7342	A/G	0.51		0.61	0.70	0.18
3B	72.92	5677	A/G	0.83		0.78	0.84	0.89
3B	84.55	3218	T/C	0.31		0.37	0.43	0.11
3B	103.72	3601	T/C	0.86		0.81	0.89	0.89
4A	53.14	5897	T/C	0.16		0.12	0.06	0.30
4A	85.19	3981	A/G	0.90		0.78	0.98	1.00
4A	117.63	3757	T/G	0.24	3758	0.16	0.07	0.51
4A	131.65	**3774**	A/G	0.20		0.07	0.05	0.53
4A	151.29	**4527**	A/G	0.61		0.59	0.23	0.96
4A	166.59	1066	T/C	0.41	1067	0.20	0.68	0.46
4A	184.19	6697	A/G	0.93		0.91	0.88	0.94
4A	193.19	**4651**	T/C	0.23		0.09	0.69	0.02
4A	198.74	3422	T/C	0.74		0.68	0.65	0.88
4B	10.92	5739	T/C	0.91		0.91	0.94	0.88
4B	82.30	7566	T/G	0.81		0.67	0.87	0.94
4B	119.74	408	A/G	0.30		0.15	0.66	0.16
4D	22.36	5381	A/G	0.92		0.96	0.93	0.84
4D	52.81	2122	T/C	0.30	55, 286, 287, 2121, 3815	0.18	0.78	0.05
5A	36.39	8154	T/G	0.86	6287	0.79	0.83	0.99
5A	58.02	114	A/G	0.54	291, 1253, 1988	0.69	0.84	0.05
5A	64.45	5529	T/C	0.89		0.95	0.96	0.73
5A	71.10	5329	T/C	0.95		0.96	1.00	0.87
5A	107.93	5668	T/C	0.45	12, 3996	0.54	0.81	0.01
5A	129.60	454	T/C	0.07		0.07	0.09	0.05
5A	184.48	**5002**	A/G	0.18	5003	0.19	0.07	0.20
5B	12.07	4790	A/C	0.62		0.65	0.82	0.38

Table S5 continued

5B	32.79	4856	T/C	0.78		0.79	0.89	0.64
5B	62.90	**5166**	T/C	0.17		0.05	0.58	0.00
5B	151.16	4774	T/C	0.08		0.08	0.05	0.11
5B	172.48	584	T/G	0.18		0.10	0.06	0.40
5B	178.56	4954	A/G	0.58		0.30	0.62	0.93
5B	205.91	1786	A/G	0.68		0.80	0.80	0.36
5D1cult	43.91	6052	T/C	0.63		0.43	0.57	0.99
5D3cult	13.24	6190	T/C	0.53	6189	0.65	0.73	0.17
6A	7.84	3627	T/C	0.12	6871	0.13	0.24	0.00
6A	45.73	7286	T/C	0.81	1523	0.76	0.87	0.83
6A	63.61	2018	A/C	0.27	2017	0.44	0.15	0.12
6A	89.35	2458	T/C	0.34	2457, 3231, 2249, 5041, 5257, 5619	0.08	0.19	0.85
6A	204.49	8595	T/C	0.35		0.42	0.33	0.27
6A	210.29	7894	T/C	0.77		0.74	0.65	0.90
6B	27.78	1850	A/C	0.75		0.83	0.63	0.71
6B	36.68	4408	A/G	0.52	7369	0.66	0.74	0.12
6B	47.66	7257	T/G	0.25		0.48	0.11	0.00
6B	62.22	4169	T/G	0.47	4924, 5966, 4848, 6101	0.69	0.20	0.40
6B	103.69	4338	T/C	0.92	4339	0.89	0.89	0.99
6B	126.02	349	T/C	0.46		0.20	0.39	0.89
6B	147.91	7098	A/C	0.59		0.48	0.72	0.62
6D1	0.00	6360	T/G	0.27		0.21	0.11	0.50
6D2	64.57	4307	A/G	0.14		0.03	0.49	0.00
7A	32.82	954	A/G	0.78		0.62	0.81	0.98
7A	55.88	3737	A/C	0.90		0.94	0.74	0.98
7A	80.94	4574	T/C	0.06		0.06	0.03	0.01
7A	105.21	6868	A/G	0.61	4845, 4846, 7755, 7756	0.39	0.63	0.89
7A	133.84	1031	T/C	0.46	1032	0.47	0.69	0.22
7B	1.73	1525	T/C	0.80		0.80	0.65	0.91
7B	14.03	2568	A/G	0.88		0.83	0.93	0.88
7B	64.03	1361	A/G	0.93		0.94	0.83	1.00
7B	98.22	1971	T/C	0.89		0.84	0.86	0.99
Favorable alleles with freq >0.95						3	7	14
Favorable alleles with freq <0.05						1	5	13
Percent >0.95 + <0.05 by subpopulation						4.00	12.00	27.00
Polymorphism Information Content (PIC) based on 5,347 SNPs						0.27	0.25	0.19

[a]Chromosome

[b]Scaled position from hexaploid wheat consensus map (Cavanagh *et al*. 2013).
[c]SNP indexes from Illumina iSelect 9K wheat assay (Cavanagh *et al*. 2013). **Bold**: QTL significant at genome-wide adjust P <0.1.
[d]SNP variant associated with *Pst* resistance is underlined.
[e]Frequency of favorable allele variant.
[f]SNP loci in linkage disequilibrium with QTL-tag SNP and significantly associated with reactions to *Pst* (IWA).
