# Supplementary material for: Genetic Architecture of Resistance to Stripe Rust in a Global Winter Wheat Germplasm Collection
Source: G3 (Bethesda). 2016 May 25;6(8):2237–53. doi: 10.1534/g3.116.028407 (PMC4978880; doi:10.1534/g3.116.028407)
Supplement: Supplemental Material [file supp_g3.116.028407_TableS6.pdf]

Table S6 Frequencies of favorable alleles of QTL-tag SNPs associated with partial resistance in population structure subgroups of the global winter wheat germplasm collection

						Allele frequency		
						Population genetic structure (subgroups by Bayesian clustering)		
					Associated SNP	1A	1B	2
Chr[a]	Pos[b]	Index[c]	Alleles[d]	Freq[e]	Index IWA[f]	(518)	(309)	(348)
1A	52.28	7715[*]	T/C	0.10		0.11	0.22	0.00
1A	72.78	3666[*]	A/G	0.54		0.31	0.90	0.62
1A	78.76	6835[*]	A/G	0.16	1615	0.24	0.18	0.02
1A	104.02	3859[*]	A/G	0.25	5493	0.16	0.02	0.55
1A	120.29	5822[*]	T/C	0.52		0.39	0.15	0.99
1A	125.50	4523	T/C	0.63		0.60	0.20	0.98
1A	132.02	**5505**[*]	A/G	0.52	475, 3284, 4934	0.42	0.14	0.95
1A	152.23	**2819**[*]	A/G	0.54		0.61	0.22	0.68
1A	176.39	4271[*]	T/C	0.48		0.51	0.73	0.26
1A	182.67	**3215**	A/G	0.67		0.67	0.85	0.51
1A	N/A	3680[*]	A/G	0.07		0.11	0.03	0.03
1B	9.56	406	A/G	0.67		0.77	0.72	0.48
1B	46.00	5963[*]	A/C	0.94		0.97	0.99	0.86
1B	63.93	5779[*]	T/C	0.20	573	0.22	0.39	0.05
1B	68.72	6018[*]	T/C	0.63		0.89	0.84	0.08
1B	76.37	606	A/G	0.15		0.25	0.11	0.01
1B	97.13	**5749**[*]	T/C	0.66	5749	0.82	0.91	0.25
1B	101.89	3097[*]	T/C	0.67		0.76	0.92	0.36
1B	136.89	545[*]	A/C	0.76		0.88	0.96	0.43
1B	N/A	**62**[*]	A/G	0.06		0.06	0.01	0.13
2A	4.67	6745[*]	A/C	0.72	5424	0.73	0.67	0.73
2A	39.36	5087[*]	A/G	0.85		0.79	0.85	0.95
2A	46.11	**2526**[*]	T/C	0.30		0.39	0.44	0.09
2A	72.31	**5824**[*]	A/G	0.58	5495	0.73	0.78	0.24
2A	82.27	690[*]	T/C	0.72		0.95	0.97	0.20
2A	158.93	544[*]	T/C	0.70		0.78	0.69	0.58
2B	4.98	8128[*]	T/C	0.84		0.75	0.83	0.99
2B	47.95	**4285**	T/C	0.25		0.20	0.35	0.21
2B	112.35	6075[*]	T/C	0.31		0.27	0.48	0.27
2B	162.84	243[*]	A/G	0.69		0.89	0.85	0.30

Table S6 continued

2B	199.32	4096[*]	T/C	0.16		0.13	0.36	0.05
2B	225.51	2343	T/C	0.18		0.10	0.56	0.01
2B	240.34	570	A/G	0.86		0.94	0.97	0.66
2B	264.59	4118[*]	A/G	0.93	3773	0.90	0.97	0.95
2B	271.78	2946[*]	A/G	0.73		0.66	0.58	0.93
2D	159.88	6851[*]	T/C	0.26		0.34	0.42	0.03
2D	173.84	2792	T/C	0.34		0.35	0.73	0.01
3A	5.88	2993	T/C	0.84		0.72	0.85	1.00
3A	62.49	132[*]	T/C	0.61		0.87	0.72	0.18
3A	82.94	133[*]	T/C	0.50		0.65	0.67	0.18
3A	135.53	**3401**[*]	T/C	0.09	2263, 2264, 2265, 2266	0.07	0.00	0.21
3B	13.82	5106[*]	T/C	0.20		0.08	0.16	0.41
3B	84.55	3218[*]	T/C	0.31		0.37	0.43	0.11
3B	103.72	3601[*]	T/C	0.85		0.81	0.89	0.89
3D3	15.21	1715	T/G	0.43	7274	0.55	0.37	0.31
4A	53.14	5897[*]	T/C	0.17		0.12	0.06	0.30
4A	61.63	4513	A/G	0.33		0.14	0.03	0.82
4A	85.19	**3981**[*]	A/G	0.89		0.78	0.98	1.00
4A	117.63	3757[*]	T/G	0.25	3758	0.16	0.07	0.51
4A	131.65	**3774**[*]	A/G	0.20		0.07	0.05	0.53
4A	166.59	1066[*]	T/C	0.40	1067	0.20	0.68	0.46
4A	184.19	**6697**[*]	A/G	0.93		0.91	0.88	0.94
4A	193.19	**4651**[*]	T/C	0.20		0.09	0.69	0.02
4A	198.74	**3422**[*]	T/C	0.73		0.68	0.65	0.88
4A	207.06	4083	A/G	0.64		0.61	0.47	0.80
4B	68.33	4347	T/C	0.51		0.66	0.65	0.20
4B	119.74	408[*]	A/G	0.28		0.15	0.66	0.16
4D	22.36	**5381**[*]	A/G	0.92		0.96	0.93	0.84
4D	52.81	2122[*]	T/C	0.27	55, 286, 287, 2121, 3815,	0.18	0.78	0.05
5A	12.83	7801	T/C	0.59		0.45	0.72	0.72
5A	36.39	8154[*]	T/G	0.86	6287	0.79	0.83	0.99
5A	58.02	114[*]	A/G	0.53	291, 1253, 1988	0.69	0.84	0.05
5A	64.45	5529[*]	T/C	0.89		0.95	0.96	0.73
5A	71.10	5329[*]	T/C	0.94		0.96	1.00	0.87
5A	101.10	2363	T/C	0.34		0.53	0.36	0.02
5A	107.93	5668[*]	T/C	0.44	12, 3996	0.54	0.81	0.01
5A	146.15	2959	T/G	0.42		0.43	0.37	0.43

Table S6 continued

5A	184.48	**5002***	A/G	0.18	5003	0.19	0.07	0.20
5B	0.00	868	T/C	0.79	757	0.88	0.83	0.63
5B	32.79	4856*	T/C	0.77		0.79	0.89	0.64
5B	62.90	5166*	T/C	0.15		0.05	0.58	0.00
5B	151.16	**4774***	T/C	0.08		0.08	0.05	0.11
5B	172.48	584*	T/G	0.18		0.10	0.06	0.40
5B	212.38	3360	T/C	0.64		0.75	0.93	0.25
5B	N/A	1621	T/C	0.94		0.94	0.96	0.93
5D3cult	13.24	6190*	T/C	0.52	6189	0.65	0.73	0.17
6A	7.84	3627*	T/C	0.11	6871	0.13	0.24	0.00
6A	45.73	7286*	T/C	0.81	1523	0.76	0.87	0.83
6A	63.61	2018*	A/C	0.27	2017	0.44	0.15	0.12
6A	115.76	6938	A/C	0.64	1856, 3269, 6811, 6812	0.87	0.82	0.16
6A	180.19	**3487**	T/C	0.80	2705	0.86	0.92	0.62
6A	204.49	8595*	T/C	0.34		0.42	0.33	0.27
6A	N/A	8617	T/C	0.08		0.16	0.01	0.00
6B	36.68	4408*	A/G	0.50	7369	0.66	0.74	0.12
6B	47.66	**7257***	T/G	0.25		0.48	0.11	0.00
6B	62.22	**4169***	T/G	0.49	4924, 5966, 4848, 6101	0.69	0.20	0.40
6B	103.69	4338*	T/C	0.92	4339	0.89	0.89	0.99
6B	126.02	**349***	T/C	0.45		0.20	0.39	0.89
6D1	0.00	6360*	T/G	0.28		0.21	0.11	0.50
6D2	64.57	4307*	A/G	0.12		0.03	0.49	0.00
7A	67.23	3351	T/C	0.75		0.77	0.37	0.99
7A	80.94	4574*	T/C	0.06		0.06	0.03	0.01
7A	93.50	2252	T/C	0.68		0.68	0.25	0.96
7A	105.21	6868*	A/G	0.59	4845, 4846, 7755, 7756	0.39	0.63	0.89
7A	133.84	1031*	T/C	0.44	1032	0.47	0.69	0.22
7B	14.03	2568*	A/G	0.87		0.83	0.93	0.88
7B	26.03	4549	T/C	0.66		0.51	0.73	0.82
7B	40.62	418	T/C	0.93		0.93	0.95	0.90
7B	50.22	2272	T/G	0.56		0.74	0.34	0.44
7B	98.22	1971*	T/C	0.89		0.84	0.86	0.99
Favorable alleles with freq >0.95						3	9	10
Favorable alleles with freq <0.05						1	7	16
Percent >0.95 + <0.05 by subpopulation						4.00	16.00	26.00
Polymorphism Information Content (PIC) based on 5,347 SNPs						0.27	0.25	0.19

[a]Chromosome

[b]Scaled position from hexaploid wheat consensus map (Cavanagh *et al*. 2013).
[c]SNP indexes from Illumina iSelect 9K wheat assay (Cavanagh *et al*. 2013). **Bold**: QTL significant at genome-wide adjust *P* <0.1.
[d]SNP variant associated with *Pst* resistance is underlined.
[e]Frequency of favorable allele variant.
[f]SNP loci in linkage disequilibrium with QTL-tag SNP and significantly associated reactions to *Pst* (IWA).
[*]Loci identified in GWAS using entire 1,175 accessions.
