# Supplementary material for: Genetic Architecture of Resistance to Stripe Rust in a Global Winter Wheat Germplasm Collection
Source: G3 (Bethesda). 2016 May 25;6(8):2237–53. doi: 10.1534/g3.116.028407 (PMC4978880; doi:10.1534/g3.116.028407)
Supplement: Supplemental Material [file supp_g3.116.028407_TableS7.pdf]

Table S7 Analysis of variance for infection type (IT) and disease severity (SEV) using 20 quantitative trait loci (QTL) with significant genome-wide associations (adjusted *P* <0.1)

	Structure		Structure and 20 QTL		Structure and 20 QTL and 24 pairwise interactions	
Source	IT	SEV	IT	SEV	IT	SEV
Q1	<.0001	<.0001	<.0001	<.0001	<.0001	<.0001
Q2	<.0001	<.0001	<.0001	<.0001	<.0001	<.0001
IWA5505			<.0001	<.0001	0.0717	0.1133
IWA3215			0.0002	<.0001	0.0017	0.0088
IWA5963			<.0001	0.0024	<.0001	0.007
IWA5915			0.0031	0.0055	<.0001	0.0005
IWA62			<.0001	0.0076	0.1409	0.4093
IWA2526			<.0001	<.0001	0.4862	0.0292
IWA5824			0.0025	0.0007	0.2676	0.227
IWA3401			<.0001	<.0001	0.7689	0.3108
IWA3981			0.0004	0.0017	<.0001	<.0001
IWA3774			0.0092	0.1701	0.0148	0.0151
IWA6697			0.004	0.0001	0.6679	0.3644
IWA4651			0.001	<.0001	<.0001	<.0001
IWA3422			<.0001	0.0002	0.387	0.2618
IWA5381			<.0001	<.0001	<.0001	<.0001
IWA5002			0.0081	0.0007	0.0005	<.0001
IWA5166			<.0001	<.0001	0.1881	0.9413
IWA8595			0.0003	<.0001	<.0001	<.0001
IWA4169			0.0199	0.0347	<.0001	<.0001
IWA7257			<.0001	0.0022	0.0083	0.7061
IWA349			<.0001	<.0001	<.0001	<.0001
IWA3215*IWA5963					0.0268	0.0257
IWA5505*IWA62					0.0032	0.033
IWA3215*IWA62					<.0001	0.0054
IWA5505*IWA2526					0.0009	0.0019
IWA3215*IWA5824					0.0396	0.3969
IWA2526*IWA5824					0.0003	0.0012
IWA5505*IWA3401					0.0035	0.0268
IWA5915*IWA3774					<.0001	0.0005
IWA5824*IWA6697					0.0336	0.0095
IWA2526*IWA4651					0.0922	0.042

Table S7 continued

IWA6697*IWA3422					0.0308	0.0937
IWA5824*IWA5381					0.0026	0.0174
IWA3774*IWA5381					0.0687	0.0067
IWA3215*IWA5002					0.0093	0.0007
IWA3401*IWA5166					0.0016	0.038
IWA4651*IWA5166					<.0001	<.0001
IWA4651*IWA8595					0.0949	0.0034
IWA5505*IWA4169					<.0001	<.0001
IWA3981*IWA4169					<.0001	<.0001
IWA5505*IWA7257					0.0006	0.0012
IWA3401*IWA349					0.0472	0.0179
IWA3774*IWA349					0.0002	0.0117
IWA4169*IWA349					0.0034	0.0143
IWA7257*IWA349					0.2899	0.0069
R^2	0.23	0.31	0.51	0.57	0.60	0.64

Models including only population structure, 20 QTL with population structure as covariate, and 20 QTL and their significant pairwise interactions with population structure as covariate were tested.
