# Supplementary material for: Genetic Architecture of Resistance to Stripe Rust in a Global Winter Wheat Germplasm Collection
Source: G3 (Bethesda). 2016 May 25;6(8):2237–53. doi: 10.1534/g3.116.028407 (PMC4978880; doi:10.1534/g3.116.028407)
Supplement: Supplemental Material [file supp_g3.116.028407_TableS8.pdf]

Table S8 Analysis of molecular variance (AMOVA), and F_{ST} values between pairs of subpopulations of subset of the global winter wheat core germplasm collection based on 5,347 SNPs

Source of variation	DF	Sum of Square	% of variation	F_{ST}		
				1A	1B	2
Entire population	2	1023409.50	21.78	-	-	-
1A	517	1836261.94	39.08	0.0000	0.1776	0.3224
1B	308	998739.64	21.26		0.0000	0.3875
2	347	839981.37	17.88			0.0000

All values are significant at P <0.001 with 10000 permutations.
