# Supplementary material for: Genetic Architecture of Resistance to Stripe Rust in a Global Winter Wheat Germplasm Collection
Source: G3 (Bethesda). 2016 May 25;6(8):2237–53. doi: 10.1534/g3.116.028407 (PMC4978880; doi:10.1534/g3.116.028407)
Supplement: Supplemental Material [file supp_g3.116.028407_TableS9.pdf]

Table S9 Summary of enrichment of genomic regions with significant marker-trait associations, with reactions to *Puccinia striiformis* f. sp. *tritici*, and loci annotations

			SNPs within QTL confidence interval			Functional annotations	
Marker	Chr	Pos (cM)	9K-anchor	90K-projected	Total	Annotated orthologs	Corresponding *R* genes
IWA5505	1A	132.0	4	11	15	11	0
IWA3215[a]	1D	164.7	14	34	48	17	2
IWA5963	1B	46.0	40	68	108	46	6
IWA5915	1B	97.1	8	94	102	38	8
IWA62	1B	Unknown	-	-	-	-	0
IWA2526	2A	46.1	3	18	21	14	4
IWA5824	2A	72.3	11	27	38	10	3
IWA3401	3A	131.4	9	32	41	26	1
IWA3981	4A	85.2	4	20	24	13	0
IWA3774	4A	131.7	7	19	26	9	0
IWA6697	4A	184.2	10	37	47	17	6
IWA4651[b]	4A	193.2	9	94	103	29	14
IWA3422[b]	4A	198.7	11	61	72	18	11
IWA5381	4D	22.4	4	12	16	7	1
IWA5002[c]	5A	184.5	9	21	30	18	2
IWA5166	5B	62.9	54	0	54	29	2
IWA8595	6A	204.5	18	2	20	9	1
IWA7257	6B	47.66	17	70	87	35	0
IWA4169	6B	62.22	14	90	104	40	2
IWA349	6B	126.0	5	18	23	7	0
**Total**			**251**	**728**	**979**	**393**	**63**

Annotations of contigs from which the QTL-tag SNPs are derived from were based on best hits in the *Brachypodium* and rice protein databases using Phytozome 10.3. *R* gene products include LRR-containing proteins, and NB-ARC domains. Detailed descriptions of the 9K anchor and 90K-projected SNPs are in Supplemental File S4.

[*]IWA3215 also maps to 1A at 182.67 cM based on the hexaploid wheat 9K consensus map (Cavanagh *et al*. 2013).

[b]*IWA4651*, and *IWA3422* are in the region of 4AL translocated from 7BS (homology 7AS/4AL/7DS).

[c]*IWA5002* is in the region of 5AL translocated from 4AL (homology 5AL/4BL/4DL).
