# Supplementary material for: Genetic Architecture of Resistance to Stripe Rust in a Global Winter Wheat Germplasm Collection
Source: G3 (Bethesda). 2016 May 25;6(8):2237–53. doi: 10.1534/g3.116.028407 (PMC4978880; doi:10.1534/g3.116.028407)
Supplement: Supplemental Material [file supp_g3.116.028407_FileS3.pdf]

File S3 Supporting information for Figure 6

Genetic Architecture of Resistance to Stripe Rust in a Global Winter Wheat Germplasm Collection

Peter Bulli[*], Junli Zhang[†], Shiaoman Chao[‡], Xianming Chen[§], Michael Pumphrey[*,1]

[*] Department of Crop and Soil Sciences, Washington State University, Pullman, WA 99164-6420, USA

[†] Department of Plant Sciences, University of California, Davis, CA 95616, USA

[‡] USDA-ARS Genotyping Laboratory, Biosciences Research Laboratory, Fargo, ND 58102

[§] USDA-ARS, Wheat Health, Genetics and Quality Research Unit, and Department of Plant Pathology, Washington State University, Pullman, WA 99164, USA

[1] Corresponding author: Michael Pumphrey. Address: Department of Crop and Soil Sciences, Washington State University, 381 Johnson Hall, PO Box 646420, Pullman, WA 99164-6420, United States of America. Phone: +1 (509) 335-0509. E-mail: m.pumphrey@wsu.edu.

File S3 Supporting information for Figure 6. Comparative mapping was performed to determine the relationships between QTL identified in this study and previously mapped *Pst* resistance genes and QTL. Start and end positions of QTL are based on percent of total length of the chromosomes in the integrated map described in Maccaferri *et al*. (2015). Numbers in column Reference number correspond to reference numbers of QTL intervals on left of the chromosomes in Figure 6. For genes included in the Catalogue of Gene Symbols for Wheat (McIntosh *et al*. 2013) or the 2013-2014 Supplement (http://wheat.pw.usda.gov/GG2/Triticum/wgc/2013/2013-2014_Supplement.pdf), the catalogue is indicated as reference, except if more recent or precise references are available (a single paper is selected). Positions of known *Yr* genes in Figure 6 are based on projection of the reported positions onto an integrated map developed by Maccaferri *et al*. (2015); and positions of intervals where *Yr* genes or QTL reside are based on published flanking markers. A linkage disequilibrium-derived ±2.3 cM confidence interval is used for all QTL identified in the present study. The positions of gene and confidence intervals described in this study are tentative because the integrated map was developed from genetic maps that do not share sufficient number of common markers. More SNP-DArT-SNP genetics maps are required for developing an integrated map with more refined positions. Allelism tests are necessary to determine whether closely linked resistance loci based on the integrated map represent different genes or alleles of same gene.

	**Relative position (%)**				
**Chr**	**Start**	**End**	***QTL* name/*Yr* gene**	**Reference**	**Reference number**
1A	0.0	5.7	*QYrid.ui-1A_Rio Blanco*	Chen *et al*. 2012	11
1A	16.5	20.1	*QYr.sgi-1A.1_Kariega*	Prins *et al*. 2011	48
1A	21.7	31.8	*QYr.tam-1AS_TAM 111*	Basnet *et al*. 2014a	6
1A	24.5	46.8	*QYr.sun-1A_Janz*	Bariana *et al*. 2010	4
1A	59.3	59.3	*IWA5754_APR*	Zegeye *et al*. 2014	68
**1A**	**64.0**	**66.3**	***QYr.wsu-1A.1 (IWA5505)***	**This study**	
1A	75.7	100.0	*QYr.tam-1AL_TAM112*	Basnet *et al*. 2014a	6
1A	77.8	80.3	*QYr.caas-1AL_Naxos*	Ren *et al*. 2012a	52
1A	79.9	80.7	*QRYr1A.1*	Jighly *et al*. 2015	26
1A	81.9	83.0	*QYr.cim-1AL_Pastor*	Rosewarne *et al*. 2012	55
**1A**	**89.0**	**91.2**	***QYr.wsu-1A.2 (IWA3215)***	**This study**	
1B	0.0	1.7	*Yr10*	Ma *et al*. 2001	41
1B	1.7	9.3	*Yr9*	Lukaszewski 2000	39
1B	5.1	28.0	*QYr.cau-1BS_AQ24788-53*	Quan *et al*. 2013	49

1B	17.3	23.5	*QYrco.wpg-1B.1_Coda*	Case *et al*. 2014	10
1B	22.1	22.8	*IWA6489_APR*	Zegeye *et al*. 2014	68
1B	23.4	24.0	*QYr.wpg-1B.1 (IWA4715)*	Naruoka *et al*. 2015	47
1B	24.8	34.6	*YrAlp*	Lin and Chen 2007	31
**1B**	**25.4**	**28.0**	***QYr.wsu-1B.1 (IWA5963)***	**This study**	
1B	28.2	34.6	*QYr.caas-1BL.1RS_SHA3/CBRD*	Ren *et al*. 2012a	52
1B	27.7	30.3	*QYr.wpg-1B.2 (IWA63)*	Naruoka *et al*. 2015	47
1B	30.1	32.1	*Yr15*	Cheng *et al*. 2014	12
1B	30.1	37.6	*QYrco.wpg-1B.2_Coda*	Case *et al*. 2014	10
1B	30.9	32.7	*YrH52*	Cheng *et al*. 2014	12
1B	32.5	32.5	*QYr.wpg-1B.3 (IWA7578)*	Naruoka *et al*. 2015	47
1B	32.7	34.5	*Yr64*	Cheng *et al*. 2014	12
1B	35.6	40.2	*IWA6891_Seedling*	Zegeye *et al*. 2014	68
1B	37.4	39.0	*Yr65*	Cheng *et al*. 2014	12
1B	39.0	41.3	*Yr24/Yr26*	Cheng *et al*. 2014	12
1B	45.0	45.0	*QYrdr.wgp-1BL.1 (gwm131)*	Hou *et al*. 2015	23
1B	41.2	54.5	*QYr.cim-1BS_Pastor*	Rosewarne *et al*. 2012	55
1B	61.3	67.2	*QYr-1B_Sachem*	Singh *et al*. 2013	59
1B	70.8	75.2	*YrExp1*	Lin and Chen 2008	32
**1B**	**72.2**	**74.9**	***QYr.wsu-1B.2 (IWA5915)***	**This study**	
1B	75.3	100.0	*QYrex.wgp-1BL_Express*	Lin and Chen 2009	33
1B	80.0	96.7	*QYr.sun-1B_Kukri*	Bariana *et al*. 2010	4
1B	80.0	90.2	*QYr.sun-1B_CPI133872*	Zwart *et al*. 2010	72
1B	84.2	96.3	*QYr.sun-1B_Wollaroi*	Bansal *et al*. 2014	3
1B	87.9	89.7	*QYr.ucw-1B (IWA3892)*	Maccaferri *et al*. 2015	42
1B	90.0	100.0	*Yr29/Lr46*	William *et al*. 2003	64
1B	90.1	94.8	*QYr.jic-1B_Guardian*	Melichar *et al*. 2008	46
1B	90.2	98.7	*QYr.tam-1B_Quaiu*	Basnet *et al*. 2014b	7
1B	90.2	98.7	*QYr.cim-1BL_Francolin#1*	Lan *et al*. 2014	28
1B	90.3	90.3	*QYrdr.wgp-1BL.2 (IWA8581)*	Hou *et al*. 2015	23
1B	90.3	96.3	*QYr-1B_Saar*	Lillemo *et al*. 2008	30
1B	93.6	100.0	*QYr.cim-1BL (Lr46/Yr29) _Pastor*	Rosewarne *et al*. 2012	55
1D	0.8	8.5	*QYr.caas-1DS_Naxos*	Ren *et al*. 2012a	52
1D	7.3	7.3	*QYrdr.wgp-1DS (IWA2268)*	Hou *et al*. 2015	23
1D	7.8	7.8	*QYr.wpg-1D.1 (IWA6960)*	Naruoka *et al*. 2015	47
1D	1.4	10.1	*QYrst.orr-1DS_Stephens*	Vazquez *et al*. 2012	62
1D	5.1	8.5	*QYr.sun-1D_CPI133872*	Zwart *et al*. 2010	72
1D	24.0	25.6	*QYr.ucw-1D (IWA980)*	Maccaferri *et al*. 2015	42
2A	0.0	6.1	*QYr.tam-2AS_TAM 111*	Basnet *et al*. 2014a	6
2A	0.0	20.0	*Yr17* (2NS -2AS translocation)	Helguera *et al.* 2003	20
2A	0.1	1.1	*IWA3122_Seedling*	Zegeye *et al*. 2014	68
2A	1.9	16.8	*QYr.uga-2AS_26R61*	Hao *et al*. 2011	19
2A	2.0	6.3	*Yr56*	Wheat Catalogue	45

2A	2.9	4.0	*QYr.ucw-2A.2 (IWA422)*	Maccaferri *et al*. 2015	42
2A	2.1	7.6	*QYr.ufs-2A (Yr16)_Cappelle-Desprez*	Agenbag *et al*. 2012	1
2A	2.3	2.5	*QYr.sun-2A_Wollaroi*	Bansal *et al*. 2014	3
2A	2.5	7.6	*QYr.inra_2AS.1_Recital*	Dedryver *et al*. 2009	14
2A	3.4	4.0	*QYr.wpg-2A.2 (IWA8274)*	Naruoka *et al*. 2015	47
2A	4.0	15.0	*QYrva.vt-2AS_VA00W-38*	Christopher *et al*. 2013	13
2A	7.6	7.6	*QYrtb.orz-2AS (cfd36)*	Vazquez *et al*. 2015	63
2A	8.3	8.3	*QYr.wpg-2A.3 (IWA3047)*	Naruoka *et al*. 2015	47
2A	8.3	11.9	*QYr.inra-2AL_Camp Remy*	Mallard *et al*. 2005	43
2A	9.9	13.1	*QYrst.orr-2AS_Stephens*	Vazquez *et al*. 2012	62
2A	6.7	14.1	*QYr.ucw-2AS_PI610750*	Lowe *et al*. 2011	36
2A	11.9	11.9	*QYr.wpg-2A.1 (gwm359)*	Naruoka *et al*. 2015	47
2A	13.3	13.3	*QYr.wpg-2A.4 (IWA7410)*	Naruoka *et al*. 2015	47
**2A**	**15.0**	**16.6**	***QYr.wsu-2A.1 (IWA2526)***	**This study**	
**2A**	**24.0**	**25.6**	***QYr.wsu-2A.2 (IWA5824)***	**This study**	
2A	26.3	27.4	*QYr.ucw-2A.3 (IWA424)*	Maccaferri *et al*. 2015	42
2A	37.9	41.4	*QYr.sun-2AS_Kukri*	Bariana *et al*. 2010	4
2A	33.3	41.4	*Yrxy2*	Zhou *et al*. 2011	71
2A	48.3	59.5	*Yr32*	Eriksen *et al*. 2004	15
2A	53.7	55.2	*IWA7339_APR*	Zegeye *et al*. 2014	68
2A	54.6	54.9	*IWA544*	Jighly *et al*. 2015	26
2A	58.3	58.3	*QYrns.orz-2AL (wPt-7011)*	Vazquez *et al*. 2015	63
2A	58.3	59.7	*QYr.caas-2AL (IWB11764)_Zhong 892*	Liu *et al*. 2015	35
2A	60.8	60.8	*QYr.wpg-2A.5 (IWA5855)*	Naruoka *et al*. 2015	47
2A	75.9	83.2	*Yr1*	Wheat Catalogue	45
2A	77.0	77.0	*QYr.wpg-2A.6 (IWA966)*	Naruoka *et al*. 2015	47
2A	79.1	100.0	*QYr.inra_2AL.2_Camp Remy*	Boukhatem *et al*. 2002	8
2B	2.3	2.3	*wPt-6271*	Jighly *et al*. 2015	26
2B	10.0	15.5	*QYr.inra-2BS_Renan*	Dedryver *et al*. 2009	14
2B	10.6	11.4	*QYrst.orr-2B.1_Stephens*	Vazquez *et al*. 2012	62
2B	13.9	13.9	*QYr.wpg-2B.1 (IWA7370)*	Naruoka *et al*. 2015	47
2B	18.6	21.3	*QYr-2B_Attila*	Rosewarne *et al*. 2008	56
2B	19.9	30.4	*QYrlu.cau-2BS1_Luke*	Guo *et al*. 2008	18
2B	20.8	20.8	*QYr.wpg-2B.2 (IWA7697)*	Naruoka *et al*. 2015	47
2B	22.7	26.4	*IWA1929-IWA2572*	Jighly *et al*. 2015	26
2B	25.1	27.4	*QYrid.ui-2B.1_IDO444*	Chen *et al*. 2012	11
2B	27.4	30.0	*QYr.sgi-2B.1_Kariega*	Prins *et al*. 2011	48
2B	27.4	29.2	*YrP81*	Wheat Catalogue	45
2B	27.5	29.1	*YrC51*	Zheng *et al*. 2014	70
2B	28.2	32.0	*QYr.sgi-2B.1_Kariega*	Ramburan *et al*. 2004	50
2B	29.6	47.9	*Yr41*	Wheat Catalogue	45
2B	29.1	32.2	*QYr.cim-2BS (Yr31)_Chapio*	Yang *et al*. 2013	67
2B	29.6	44.5	*QYrlo.wpg-2BS_Louise*	Carter *et al*. 2009	9

2B	30.4	50.2	*QYrid.ui-2B.2_IDO444*	Chen *et al*. 2012	11
2B	30.4	36.4	*QYrst.orr-2BS.2_Stephens*	Vazquez *et al*. 2012	62
2B	30.6	32.3	*QYrlu.cau-2BS2_Luke*	Guo *et al*. 2008	18
2B	31.4	31.4	*IWA2624_Seedling*	Zegeye et al. 2014	68
2B	32.3	33.5	*YrH9014*	Ma *et al*. 2013	40
2B	32.3	49.1	*QYr.caas-2BS_Pingyuan 50*	Lan *et al*. 2010	27
2B	34.4	36.4	*Yr27*	Wheat Catalogue	45
2B	35.4	44.3	*QYr-2B_Opata 85*	Boukhatem *et al*. 2002	8
2B	36.6	47.9	*QYr.tam-2BL_TAM 111*	Basnet *et al*. 2014a	6
2B	30.4	44.3	*YrKK*	Wheat Catalogue	45
2B	39.8	49.1	*QYr.ucw-2B_UC1110*	Lowe *et al*. 2011	36
2B	42.1	50.5	*QYr.inra-2B.1_Camp Remy*	Mallard *et al*. 2005	43
2B	44.3	47.9	*QYr.cim-2BS_Francolin#1*	Lan *et al*. 2014	28
2B	59.0	59.0	*IWA1488_APR*	Zegeye *et al*. 2014	68
2B	59.0	65.7	*QRYr2B.2*	Jighly *et al*. 2015	26
2B	59.8	60.0	*QYr.inra-2B.2_Camp Remy*	Mallard *et al*. 2005	43
2B	60.1	66.6	*QYr.caas-2BL_Naxos*	Ren *et al*. 2012a	52
2B	62.1	73.6	*QYraq.cau-2BL_Aquileja*	Guo *et al*. 2008	18
2B	62.1	64.5	*Yr5*	McGrann *et al*. 2014	44
2B	63.9	66.1	*Yr44*	Xu *et al*. 2013	66
2B	67.5	67.5	*QYrdr.wgp-2BL (IWA6286)*	Hou *et al*. 2015	23
2B	71.4	71.4	*QYrns.orz-2BL (wPt-0950)*	Vazquez *et al*. 2015	63
2B	66.1	78.1	*Yr53*	Xu *et al*. 2013	66
2B	78.1	82.1	*Yr43*	Xu *et al*. 2013	66
2B	86.8	90.7	*Yr3*	Wheat Catalogue	45
2B	88.3	91.8	*QYr.caas-2BL.3 (IWB36313)_Linmai 2*	Liu *et al*. 2015	35
2B	89.2	90.8	*QYr-2B_Avocet*	Rosewarne *et al*. 2008	56
2D	0.0	4.8	*QYr.caas-2DS_Libellula*	Lu *et al*. 2009	37
2D	0.7	0.7	*QYr.wpg-2D.1 (IWA1939)*	Naruoka *et al*. 2015	47
2D	24.5	34.2	*QYr.caas-2DS.2_Lumai 21*	Ren *et al*. 2015	54
2D	34.2	36.9	*QYr.ufs-2DS_ Cappelle-Desprez*	Agenbag *et al*. 2012	1
2D	31.4	55.8	*QYr.inra-2DS_Camp Remy*	Mallard *et al*. 2005	43
2D	44.1	55.8	*QYr.caas-2DL_Naxos*	Ren *et al*. 2012a	52
2D	51.0	51.0	*QYr.wpg-2D.2 (IWA6851)*	Naruoka *et al*. 2015	47
2D	55.6	61.3	*QYr.jic-2D_Guardian*	Melichar *et al*. 2008	46
2D	68.4	79.5	*QYr.tam_2D_Quaiu*	Basnet *et al*. 2014b	7
2D	72.1	81.5	*QYr.jic-2D_Briagdier*	Jagger *et al*. 2011	25
2D	67.1	74.5	*Yr55*	Wheat Catalogue	45
2D	70.7	80.3	*Yr54*	Wheat Catalogue	45
3A	5.0	9.2	*QYr-1B_Saar*	Lillemo *et al*. 2008	30
3A	9.5	22.3	*QYr-3A_Seedling*	Jighly *et al*. 2015	26
3A	10.4	20.4	*QYr.caas-3AS (IWB48450)_Zhong 892*	Liu *et al*. 2015	35
3A	12.8	22.4	*QYrst.orr-3AL_Stephens*	Vazquez *et al*. 2012	62

3A	37.4	42.0	*QYr.cau-3AL_AQ24788-83*	Quan *et al*. 2013	49
3A	42.3	42.3	*IWA8630_APR*	Zegeye *et al*. 2014	68
3A	52.3	56.6	*IWA5849-IWA3739*	Jighly *et al*. 2015	26
**3A**	**53.8**	**55.9**	***IWA7877_Seedling***	**This study**	
3A	59.7	59.7	*QYrdr.wgp-3AL (IWA6834)*	Hou *et al*. 2015	23
**3A**	**61.5**	**63.6**	***QYr.wsu-3A (IWA3401)***	**This study**	
3A	69.7	75.5	*QYr.cim-3A_Avocet*	Rosewarne *et al*. 2012	55
3B	0.0	6.2	*QYr-3B_Opata 85*	Singh *et al*. 2000	58
3B	0.0	1.8	*Yr4*	Wheat Catalogue	45
3B	0.0	2.3	*Yr57*	Wheat Catalogue	45
3B	2.3	6.9	*Yr30*	Suenaga *et al*. 2003	60
3B	0.0	5.3	*QYr-3B.1_Pavon 76*	William *et al*. 2006	65
3B	0.0	6.7	*QYr.cim-3BS (Yr30) _Chapio*	Yang *et al*. 2013	67
3B	2.1	13.7	*QYr.tam-3B_Quaiu*	Basnet *et al*. 2014b	7
3B	2.3	14.9	*QYr.cim-3BS.2_Francolin#1*	Lan *et al*. 2014	28
3B	2.3	7.0	*QYr-3B_Oligoculm*	Suenaga *et al*. 2003	60
3B	2.3	5.7	*QYr-3B_Alturas*	Zhao *et al*. 2012	69
3B	2.8	7.9	*QYr.inra-3BS_Renan*	Dedryver *et al*. 2009	14
3B	4.4	6.7	*QYr.ucw-3BS_UC1110*	Lowe *et al*. 2011	36
3B	5.4	20.7	*QYrco.wpg-3B.1_Brundage*	Case *et al*. 2014	10
3B	6.5	8.0	*QYr.ucw-3B.2 (IWA5202)*	Maccaferri *et al*. 2015	42
3B	6.7	11.6	*QYr.uga-3BS.1_AGS2000*	Hao *et al*. 2011	19
3B	8.2	8.2	*IWA6651_Seedling*	Zegeye *et al*. 2014	68
3B	14.9	16.5	*Yrns-B1*	Wheat Catalogue	45
3B	15.7	21.0	*wPt-800213-IWA6510*	Jighly *et al*. 2015	26
3B	28.3	37.6	*QYr.sun-3B_Kukri*	Bariana *et al*. 2010	4
3B	28.9	29.7	*QYr.caas-3BS (IWB35069)_Zhong 892*	Liu *et al*. 2015	35
3B	39.3	41.5	*QYrco.wpg-3B.2_Brundage*	Case *et al*. 2014	10
3B	42.1	60.7	*QYr.cim-3B_Pastor*	Rosewarne *et al*. 2012	55
3B	46.9	46.9	*QRYr3B.2*	Jighly et al. 2015	26
3B	50.5	60.9	*QYr.inra-3Bcentr_Renan*	Dedryver *et al*. 2009	14
3B	51.2	65.1	*QYrpi.vt-3BL_VA00W-38*	Christopher *et al*. 2013	13
3B	53.2	53.5	*IWA3834_APR*	Zegeye *et al*. 2014	68
3B	68.6	68.6	*QYrns.orz-3BL (wPt-3107)*	Vazquez *et al*. 2015	63
3B	68.6	71.9	*QYr.sun-3B_Wollaroi*	Bansal *et al*. 2014	3
3B	85.6	98.1	*QYrid.ui-3B_Rio Blanco*	Chen *et al*. 2012	11
3B	86.5	86.5	*QYr.wpg-3B.1 (IWA6930)*	Naruoka *et al*. 2015	47
3B	88.2	99.5	*QYrex.wgp-3BL_Express*	Lin and Chen 2009	33
3D	0.4	2.4	*Yr66*	Wheat Catalogue	45
3D	7.1	8.6	*Yr49*	Wheat Catalogue	45
3D	4.6	24.1	*QYr.tam-3D_Quaiu*	Basnet et al. 2014b	7
3D	60.2	72.0	*Yr45*	Wheat Catalogue	45

3D	92.1	92.1	*IWA3012_Seedling*	Zegeye *et al*. 2014	68
4A	30.7	30.9	*QYr.wpg-4A.1 (IWA1940)*	Naruoka *et al*. 2015	47
**4A**	**38.8**	**40.9**	***QYr.wsu-4A.1 (IWA3981)***	**This study**	
4A	55.0	55.0	*IWA3756_APR*	Zegeye *et al*. 2014	68
**4A**	**60.5**	**62.7**	***QYr.wsu-4A.2 (IWA3774)***	**This study**	
4A	70.5	80.8	*Yr51*	Randhawa *et al*. 2014	51
4A	71.0	72.2	*IWA8475_APR*	Zegeye *et al*. 2014	68
4A	74.2	79.1	*QYr-4A_Sachem*	Singh *et al*. 2013	59
4A	74.2	81.2	*QYr.orr-4AL_Stephens*	Vazquez *et al*. 2012	62
4A	74.4	80.2	*QYr.sgi-4A.2_Kariega*	Ramburan *et al*. 2004	50
4A	76.1	84.5	*QYr.sgi-4A.1_Kariega*	Ramburan *et al*. 2004	50
4A	78.8	85.7	*QYrid.ui-4A_IDO444*	Chen *et al*. 2012	11
4A	80.1	80.1	*QYrns.orz-4AL (wPt-6440)*	Vazquez *et al*. 2015	63
4A	80.1	94.8	*QYr.sgi-4A.1 and 4A.2_Kariega*	Prins *et al*. 2011	48
4A	83.3	85.7	*Yr60*	Herrera-Foessel *et al*. 2015	22
4A	84.3	85.8	*QYr.ucw-4A (IWA1034)*	Maccaferri *et al*. 2015	42
**4A**	**85.1**	**87.3**	***QYr.wsu-4A.3 (IWA6697)***	**This study**	
**4A**	**89.3**	**91.5**	***QYr.wsu-4A.4 (IWA4651)***	**This study**	
**4A**	**91.9**	**94.1**	***QYr.wsu-4A.5 (IWA3422)***	**This study**	
4A	92.3	92.3	*QYrtb.orz-4AL (wPt-1007)*	Vazquez *et al*. 2015	63
4B	32.9	40.7	*QYr-4B_Sachem*	Singh *et al*. 2013	59
4B	34.6	51.9	*QYr.ufs-4B_Palmiet*	Agenbag *et al*. 2012	1
4B	36.3	64.0	*QYr.sun-4B_Janz*	Zwart *et al*. 2010	72
4B	39.0	68.6	*Yr50*	Liu *et al*. 2013	34
4B	39.4	51.9	*QYr-4B_Avocet*	William *et al*. 2006	65
4B	49.2	52.4	*QYr.caas-4BL_Libellula*	Lu *et al*. 2009	37
4B	49.7	53.8	*QYr.ui-4B_Rio Blanco*	Chen *et al*. 2012	11
4B	52.1	58.1	*Yr62*	Lu *et al*. 2014	38
4B	54.8	62.1	*QYr.jic-4B_Alcedo*	Jagger *et al*. 2011	25
4B	55.4	55.4	*QYr.wpg-4B.1 (IWA4348)*	Naruoka *et al*. 2015	47
4B	58.1	60.4	*QYr.vt-4BL_VA00W-38*	Christopher *et al*. 2013	13
4B	58.1	62.1	*QYr.jic-4B_Guardian*	Melichar *et al*. 2008	46
4B	65.8	73.3	*QYr-4B_Oligoculm*	Suenaga *et al*. 2003	60
4B	82.6	86.3	*QYr.wpg-4B.2 (IWA3994)*	Naruoka *et al*. 2015	47
4D	9.4	11.4	*Yr28*	Wheat Catalogue	45
4D	14.0	15.9	*YrAS2388*	Wheat Catalogue	45
4D	18.4	34.0	*QYr.cim-4DS_Pastor*	Rosewarne *et al*. 2012	55
**4D**	**19.8**	**24.3**	***QYr.wsu-4D (IWA5381)***	**This study**	
4D	36.6	42.1	*QYr.caas-4DL.2_Lumai 21*	Ren *et al*. 2015	54
4D	45.4	53.7	*QYr.caas-4DL_Bainong 64*	Ren *et al*. 2012b	53
4D	47.2	49.1	*QYr.ucw-4D (IWA5375)*	Maccaferri *et al*. 2015	42
4D	49.5	50.8	*Yr46/Lr67*	Herrera-Foessel *et al*. 2011	21
4D	54.0	62.3	*QYr-4D_Oligoculm*	Suenaga *et al*. 2003	60

5A	15.0	15.0	*IWA4767_APR*	Zegeye *et al*. 2014	68
5A	22.3	23.9	*QYr.cau-5AS_AQ24788-53*	Quan *et al*. 2013	49
5A	28.8	28.8	*IWA7129_Seedling*	Zegeye *et al*. 2014	68
5A	35.7	42.3	*QYr.cim-5AL_Francolin#1*	Lan *et al*. 2014	28
5A	40.1	40.1	*IWA6949_APR*	Zegeye *et al*. 2014	68
5A	66.8	79.2	*QYr-5A_Opata 85*	Boukhatem *et al*. 2002	8
5A	66.8	79.9	*QYr.cim-5AL_Pastor*	Rosewarne *et al*. 2012	55
5A	81.2	84.6	*QYr.caas-5AL.2_SHA3/CBRD*	Ren *et al*. 2012a	52
5A	81.5	88.3	*QYr.caas-5AL_Pingyuan 50*	Lan *et al*. 2010	27
5A	86.8	100	*Yr48*	Lowe *et al*. 2011	36
**5A**	**84.2**	**86.3**	***QYr.wsu-5A (IWA5002)***	**This study**	
5A	84.5	100.0	*QYr.ucw-5AL_PI610750*	Lowe *et al*. 2011	36
5A	86.7	88.1	*QYr.ucw-5A.1 (IWA6988)*	Maccaferri *et al*. 2015	42
5A	89.7	89.7	*QYrns.orz-5AL (gwm291)*	Vazquez *et al*. 2015	63
5A	89.9	89.9	*QYrdr.wgp-5AL (IWA2646)*	Hou *et al*. 2015	23
5A	89.9	100.0	*Yr34*	Bariana *et al*. 2006	5
5B	3.3	10.0	*Yr47*	Bansal *et al*. 2011	2
5B	11.4	16.7	*QYr.uga-5B_AGS2000*	Hao *et al*. 2011	19
5B	20.8	37.0	*QYr.cim-5BL_Chapio*	Yang *et al*. 2013	67
**5B**	**27.3**	**29.2**	***QYr.wsu-5B (IWA5166)***	**This study**	
5B	37.0	41.2	*QYr.ufs-5B_Cappelle-Desprez*	Agenbag *et al*. 2012	1
5B	37.5	38.9	*QYr.tem-5B.1_Flinor*	Feng *et al*. 2011	17
5B	42.1	46.5	*QYr.inra-5B.1_Camp Remy*	Mallard *et al*. 2005	43
5B	43.3	44.4	*QYrdr.wgp-5BL.2 (IWA6867)*	Hou *et al*. 2015	23
5B	48.8	48.8	*QYrns.orz-5BL (wPt-8285)*	Vazquez *et al*. 2015	63
**5B**	**52.1**	**54.1**	***IWA7815_Seedling***	**This study**	
5B	53.8	55.2	*YrExp2*	Lin and Chen 2008	32
5B	45.0	50.9	*QYrco.wpg-5B_Coda*	Case *et al*. 2014	10
5B	45.2	47.6	*QYr.caas-5BL.1_Libellula*	Lu *et al*. 2009	37
5B	47.6	52.3	*QYr-5B_Oligoculm*	Suenaga *et al*. 2003	60
5B	52.7	52.7	*QYrtb.orz-5BL (wPt-6105)*	Vazquez *et al*. 2015	63
5B	59.7	59.7	*IWA3002_Seedling*	Zegeye *et al*. 2014	68
5B	61.1	61.1	*QRYr5B.1 (IWA2565, IWA3432)*	Jighly *et al*. 2015	26
5B	63.3	72.9	*QYr.sun-5B_Janz*	Bariana *et al*. 2010	4
5B	65.4	67.4	*QYr.caas-5BL.3_SHA3/CBRD*	Ren *et al*. 2012a	52
5B	70.3	75.7	*QYr.tem-5B.2_Flinor*	Feng *et al*. 2011	17
5B	70.3	73.2	*QYr.caas-5BL.2_Libellula*	Lu *et al*. 2009	37
5B	70.3	74.8	*QYr.inra-5BL.2_Camp Remy*	Mallard *et al*. 2005	43
5B	84.4	97.1	*QYr.sun-5B_Wollaroi*	Bansal *et al*. 2014	3
5B	84.8	100.0	*QYr.ui-5B_IDO444*	Chen *et al*. 2012	11
5B	90.3	90.3	*QYrdr.wgp-5BL.1 (IWA6271)*	Hou *et al*. 2015	23
5D	0.0	4.0	*Yr40*	Wheat Catalogue	45
5D	17.5	33.8	*Yr.caas-5DS_Jingshuang 16*	Ren *et al*. 2015	54

5D	54.3	57.6	*QYr.caas-5DL (IWA4087)_Zhong 892*	Liu *et al*. 2015	35
5D	92.1	92.1	*QYrdr.wgp-5DL (IWA8331)*	Hou *et al*. 2015	23
5D	95.7	97.9	*QYrco.wpg-5D_Brundage*	Case *et al*. 2014	10
6A	0.0	7.1	*QYr.uga-6AS_26R61*	Hao *et al*. 2011	19
6A	3.8	7.1	*QYr.wgp-6AS_Express*	Lin and Chen 2009	33
6A	7.6	17.8	*QYr.cim-6A_Avocet*	Rosewarne *et al*. 2012	55
6A	37.6	45.3	*QYr.caas-6AL (IWB39473)_Zhong 892*	Liu et al. 2015	35
6A	46.7	49.2	*QRYr6A.1*	Jighly *et al*. 2015	26
6A	48.2	48.2	*QYr.wpg-6A.1 (IWA3023)*	Naruoka *et al*. 2015	47
6A	49.7	68.0	*QYr.cim-6AL_Francolin#1*	Lan *et al*. 2014	28
6A	56.9	67.3	*YrLM168*	Feng *et al*. 2014	16
6A	61.6	66.8	*QYr.ufs-6A_Kariega*	Prins *et al*. 2011	48
6A	62.7	74.6	*QYr-6A_Saar*	Lillemo *et al*. 2008	30
6A	63.4	63.4	*IWA7994_APR*	Zegeye *et al*. 2014	68
6A	63.9	73.1	*QYr-6A_Avocet*	William *et al*. 2006	65
6A	71.3	74.7	*QYrpl.orr-6AL_Stephens*	Vazquez *et al*. 2012	62
**6A**	**71.7**	**73.3**	***QYr.wsu-6A (IWA8595)***	**This study**	
6A	75.2	75.2	*QYrtb.orz-6AL (wPt-4229)*	Vazquez *et al*. 2015	63
6B	0	14.9	*Yr35*	Wheat Catalogue	45
6B	1.3	1.3	*QYrdr.wgp-6BL.1 (IWA3297)*	Hou *et al*. 2015	23
6B	6.9	12.3	*QYr.ufs-6B_Kariega*	Prins *et al*. 2011	48
6B	9.4	19.7	*QYr.tam-6BS_TAM 111*	Basnet *et al*. 2014a	6
6B	10.3	16.4	*QYr.caas-6BS.2_Naxos*	Ren *et al*. 2012b	53
6B	12.6	16.4	*QYr.caas-6BS_Bainong 64*	Ren *et al*. 2012b	53
6B	15.9	21.4	*QYr.wgp-6BS.2_Stephens*	Santra *et al*. 2008	57
6B	17.1	34.8	*QYr.sun-6B_Janz*	Bariana *et al*. 2010	4
6B	23.1	28.6	*QYr-6B_Oligoculm*	Suenaga *et al*. 2003	60
6B	23.1	25.1	*Yr36*	Uauy *et al*. 2005	61
**6B**	**23.2**	**25.6**	***QYr.wsu-6B.1 (IWA7257)***	**This study**	
6B	23.9	25.6	*QYr.ucw-6B (IWA7257)*	Maccaferri *et al*. 2015	42
6B	24.1	34.8	*QYr.wgp-6B.1_Stephens*	Santra *et al*. 2008	57
6B	24.4	24.4	*QYr.wpg-6B.1 (IWA7257)*	Naruoka *et al*. 2015	47
**6B**	**25.8**	**34.8**	***QYr.wsu-6B.2 (IWA4169)***	**This study**	
6B	27.4	42.3	*QYr.caas-6BS_Pingyuan 50*	Lan *et al*. 2010	27
6B	42.3	50.2	*QYr-6B_Pavon 76*	William *et al*. 2006	65
6B	42.3	50.2	*QYr.inra-6B_Renan*	Dedryver *et al*. 2009	14
6B	43.5	43.5	*QYrdr.wgp-6BL.2 (IWA6420)*	Hou *et al*. 2015	23
**6B**	**63.4**	**65.7**	***QYr.wsu-6B.3 (IWA349)***	**This study**	
6B	70.0	79.9	*QYr.cim-6BL_Pastor*	Rosewarne *et al*. 2012	55
6B	76.8	76.8	*QYr.wpg-6B.2 (IWA3222)*	Naruoka *et al*. 2015	47
6D	42.4	56.1	*QYr.ufs-6D_Cappelle-Desprez*	Agenbag *et al*. 2012	1
6D	47.9	49.8	*QYr.ucw-6D (IWA167)*	Maccaferri *et al*. 2015	42
6D	71.6	77.8	*QYr-6D_W-7984*	Boukhatem *et al*. 2002	8

6D	92.4	92.4	*IWA4455_Seedling*	Zegeye *et al*. 2014	68
7A	0.0	6.1	*QYr.cim-7AS_Avocet*	Rosewarne *et al*. 2012	55
7A	6.5	10.1	*QYr.inra-7A_Recital*	Dedryver *et al*. 2009	14
7A	12.5	22.8	*QYr.caas-7A_Jingshuan 16*	Ren *et al*. 2012b	53
7A	12.5	40.2	*QYr.sun-7A_CPI133872*	Zwart *et al*. 2010	72
7A	16.6	16.6	*IWA2710_APR*	Zegeye *et al*. 2014	68
7A	19.5	32.4	*Yr61*	Wheat Catalogue	45
7A	39.8	39.8	*IWA4996_APR*	Zegeye *et al*. 2014	68
7A	42.0	49.3	*Yrxy1*	Zhou *et al*. 2011	71
7A	54.4	54.4	*IWA4621_APR*	Zegeye *et al*. 2014	68
7A	58.4	58.4	*IWA4187_Seedling*	Zegeye *et al*. 2014	68
7A	62.3	69.8	*QYr.caas-7AL (IWB24300)_Zhong 892*	Liu *et al*. 2015	35
7A	69.9	77.1	*QYr.orr-7A_Stephens*	Vazquez *et al*. 2012	62
7A	74.4	85.5	*QYr.cim-7BL_Avocet*	Rosewarne *et al*. 2012	55
7A	74.6	100.0	*QYr.sgi-7A_Kariega*	Prins *et al*. 2011	48
**7A**	**75.8**	**77.6**	***IWA7592_Seedling***	**This study**	
7B	0.0	1.0	*Yr63*	Wheat Catalogue	45
7B	21.0	27.7	*QYr-7B_Oligoculm*	Suenaga et al. 2003	60
7B	27.5	48.1	*Yr39*	Lin and Chen 2007	31
7B	33.3	33.3	*IWA8469_APR*	Zegeye *et al*. 2014	68
7B	33.8	56.3	*QYr.caas-7B.1_SHA3/CBRD*	Ren *et al*. 2012a	52
7B	41.6	47.8	*QHtap.wsu-7BL_Alpowa*	Lin and Chen 2007	31
7B	41.8	45.9	*QYr.orr-7BS_Stephens*	Vazquez *et al*. 2012	62
7B	43.8	58.7	*QYr.sun-7B_Kukri*	Bariana *et al*. 2010	4
7B	61.5	61.5	*IWA4309_APR*	Zegeye *et al*. 2014	68
7B	68.8	75.2	*QYr-7B_Tiritea*	Imtiaz *et al*. 2004	24
7B	70.0	70.0	*QYrtb.orz-7BL (wPt-2356)*	Vazquez *et al*. 2015	63
7B	71.8	73.6	*QYr.caas-7BL.2_SHA3/CBRD*	Ren *et al*. 2012a	52
7B	73.5	87.6	*QYr.cim-7BL_Pastor*	Rosewarne *et al*. 2012	55
7B	75.5	77.5	*Yr67*	Wheat Catalogue	45
7B	80.4	82.7	*YrZH84*	Wheat Catalogue	45
7B	80.5	86.2	*QYr-7BL_Strongfield*	Singh *et al*. 2013	59
7B	80.9	84.7	*Yr59*	Wheat Catalogue	45
7B	81.6	83.2	*Yr52*	Wheat Catalogue	45
7B	82.6	88.4	*QYr-7B_Attila*	Rosewarne *et al*. 2008	56
7B	82.7	87.8	*YrC591*	Wheat Catalogue	45
7D	19.8	19.8	*wPt-668026*	Jighly *et al*. 2015	26
7D	26.1	33.6	*Yr18/Lr34* (csLV23)	Yang *et al*. 2013	67
7D	32.1	37.8	*QYr.caas-7DS.1 (IWB56317)_Zhong 892*	Liu *et al*. 2015	35
7D	49.8	60.2	*Yr33*	Wheat Catalogue	45

References ordered in alphabetically. Numbers on left correspond to numbers of references for the QTL regions on right side of Figure 6.

1. Agenbag, G. M., Z. A. Pretorius, L. A. Boyd, C. M. Bender, and R. Prins, 2012 Identification of adult plant resistance to stripe rust in the wheat cultivar Cappelle-Desprez. Theor. Appl. Genet. **125:** 109–120.
2. Bansal, U. K., K. L. Forrest, M. J. Hayden, H. Miah, D. Singh *et al.*, 2011 Characterisation of a new stripe rust resistance gene *Yr47* and its genetic association with the leaf rust resistance gene *Lr52*. Theor. Appl. Genet. **122:** 1461–1466.
3. Bansal, U. K., A. G. Kazi, B. Singh, R. A. Hare, and H. S. Bariana, 2014 Mapping of durable stripe rust resistance in a durum wheat cultivar Wollaroi. Mol. Breed. **33:** 51–59.
4. Bariana, H. S., U. K. Bansal, A. Schmidt, A. Lehmensiek, J. Kaur *et al.*, 2010 Molecular mapping of adult plant stripe rust resistance in wheat and identification of pyramided QTL genotypes. Euphytica **176:** 251–260.
5. Bariana, H. S., N. Parry, I. R. Barclay, R. Loughman, R. J. McLean *et al.*, 2006 Identification and characterization of stripe rust resistance gene *Yr34* in common wheat. Theor. Appl. Genet. **112:** 1143–1148.
6. Basnet, B. R., A. M. H. Ibrahim, X. Chen, R. P. Singh, E. R. Mason *et al.*, 2014a Molecular mapping of stripe rust resistance in hard red winter wheat TAM 111 adapted to the U.S. high plains. Crop Sci. **54:** 1361-1373.
7. Basnet, B. R., R. P. Singh, A. M. H. Ibrahim, S. A. Herrera-Foessel, J. Huerta-Espino *et al.*, 2014b Characterization of *Yr54* and other genes associated with adult plant resistance to yellow rust and leaf rust in common wheat Quaiu 3. Mol. Breed. **33:** 385–399.
8. Boukhatem, N., P. V. Baret, D. Mingeot, and J. M. Jacquemin, 2002 Quantitative trait loci for resistance against yellow rust in two wheat-derived recombinant inbred line populations. Theor. Appl. Genet. **104:** 111–118.
9. Carter, A. H., X. M. Chen, K. Garland-Campbell, and K. K. Kidwell, 2009 Identifying QTL for high-temperature adult-plant resistance to stripe rust (*Puccinia striiformis* f. sp. *tritici*) in the spring wheat (*Triticum aestivum* L.) cultivar "Louise." Theor. Appl. Genet. **119:** 1119–1128.

10. Case, A. J., Y. Naruoka, X. Chen, K. A. Garland-Campbell, R. S. Zemetra *et al*., 2014 Mapping stripe rust resistance in a BrundageXCoda winter wheat recombinant inbred line population. PLoS ONE **9:** e91758.

11. Chen, J., C. Chu, E. J. Souza, M. J. Guttieri, X. Chen *et al*., 2012 Genome-wide identification of QTL conferring high-temperature adult-plant (HTAP) resistance to stripe rust (*Puccinia striiformis* f. sp. *tritici*) in wheat. Mol. Breed. **29:** 791–800.

12. Cheng, P., L. S. Xu, M. N. Wang, D. R. See, and X. M. Chen, 2014 Molecular mapping of genes *Yr64* and *Yr65* for stripe rust resistance in hexaploid derivatives of durum wheat accessions PI 331260 and PI 480016. Theor. Appl. Genet. 1**27:** 2267–2277.

13. Christopher, M. D., S. Liu, M. D. Hall, D. S. Marshall, M. O. Fountain *et al*., 2013 Identification and mapping of adult plant stripe rust resistance in soft red winter wheat VA00W-38. Crop Sci. **53:** 871-879.

14. Dedryver, F., S. Paillard, S. Mallard, O. Robert, M. Trottet *et al*., 2009 Characterization of genetic components involved in durable resistance to stripe rust in the bread wheat “Renan”. Phytopathology **99:** 968–973.

15. Eriksen, L., F. Afshari, M. J. Christiansen, R. A. McIntosh, A. Jahoor *et al*., 2004 *Yr32* for resistance to stripe (yellow) rust present in the wheat cultivar Carstens V. Theor. Appl. Genet. **108:** 567–575.

16. Feng, J., G. Chen, Y. Wei, Y. Liu, Q. Jiang *et al*., 2014 Identification and genetic mapping of a recessive gene for resistance to stripe rust in wheat line LM168-1. Mol. Breed. **33:** 601–609.

17. Feng, J., L. L. Zuo, Z. Y. Zhang, R. M. Lin, Y. Y. Cao *et al*., 2011 Quantitative trait loci for temperature-sensitive resistance to *Puccinia striiformis* f. sp. *tritici* in wheat cultivar Flinor. Euphytica **178:** 321–329.

18. Guo, Q., Z. J. Zhang, Y. B. Xu, G. H. Li, J. Feng *et al*., 2008 Quantitative trait loci for high-temperature adult-plant and slow-rusting resistance to *Puccinia striiformis* f. sp. *tritici* in wheat cultivars. Phytopathology **98:** 803–809.

19. Hao, Y., Z. Chen, Y. Wang, D. Bland, J. Buck *et al*., 2011 Characterization of a major QTL for adult plant resistance to stripe rust in US soft red winter wheat. Theor. Appl. Genet. **123:** 1401–1411.

20. Helguera, M., I. A. Khan, J. Kolmer, D. Lijavetzky, L. Zhong-qi *et al*., 2003 PCR assays for the cluster of rust resistance genes and their use to develop isogenic hard red spring wheat lines. Crop Sci. **43:** 1839–1847.

21. Herrera-Foessel, S. A., E. S. Lagudah, J. Huerta-Espino, M. J. Hayden, H. S. Bariana *et al*., 2011 New slow-rusting leaf rust and stripe rust resistance genes *Lr67* and *Yr46* in wheat are pleiotropic or closely linked. Theor. Appl. Genet. **122:** 239–249.

22. Herrera-Foessel, S. A., R. P. Singh, and C. X. Lan, 2015 *Yr60*, a gene conferring moderate resistance to stripe rust in wheat. Plant Dis. **99:** 508-511.

23. Hou, L., X. Chen, M. Wang, D. R. See, S. Chao *et al*., 2015 Mapping a large number of QTL for durable resistance to stripe rust in winter wheat Druchamp using SSR and SNP markers. PLoS ONE **10:** e0126794.

24. Imtiaz, M., M. Ahmad, M. G. Cromey, W. B. Griffin, and J. G. Hampton, 2004 Detection of molecular markers linked to the durable adult plant stripe rust resistance gene *Yr18* in bread wheat (*Triticum aestivum* L.). Plant Breed. **123:** 401–404.

25. Jagger, L. J., C. Newell, S. T. Berry, R. MacCormack, and L. A. Boyd, 2011 The genetic characterisation of stripe rust resistance in the German wheat cultivar Alcedo. Theor. Appl. Genet. **122:** 723–733.

26. Jighly, A., B. C. Oyiga, F. Makdis, K. Nazari, O. Youssef *et al*., 2015 Genome-wide DArT and SNP scan for QTL associated with reistsance to strip rust (*Puccinia striiformis* f. sp. *tritici*) in elite ICARDA wheat (*Triticum aestivum* L.) germplasm. Theor. Appl. Genet. **128:**1277–1295.

27. Lan, C., S. Liang, X. Zhou, G. Zhou, Q. Lu *et al*., 2010 Identification of genomic regions controlling adult-plant stripe rust resistance in chinese landrace Pingyuan 50 through bulked segregant analysis. Phytopathology **100:** 313–318.

28. Lan, C., G. M. Rosewarne, R. P. Singh, S. A. Herrera-Foessel, J. Huerta-Espino *et al*., 2014 QTL characterization of resistance to leaf rust and stripe rust in the spring wheat line Francolin#1. Mol. Breed. **34:** 789–803.

29. Li, Z. F., T. C. Zheng, Z. H. He, G. Q., Li, S. C. Xu *et al*., 2006 Molecular tagging of stripe rust resistance gene *YrZH84* in Chinese wheat line Zhou 8425B. Theor. Appl. Genet. **112:** 1098–1103.

30. Lillemo, M., B. Asalf, R. P. Singh, J. Huerta-Espino, X. M. Chen *et al*., 2008 The adult plant rust resistance loci *Lr34/Yr18* and *Lr46/Yr29* are important determinants of partial resistance to powdery mildew in bread wheat line Saar. Theor. Appl. Genet. **116:** 1155–1166.

31. Lin, F., and X. M. Chen, 2007 Genetics and molecular mapping of genes for race-specific all-stage resistance and non-race-specific high-temperature adult-plant resistance to stripe rust in spring wheat cultivar Alpowa. Theor. Appl. Genet. **114:** 1277–1287.

32. Lin, F., and X. M. Chen, 2008 Molecular mapping of genes for race-specific overall resistance to stripe rust in wheat cultivar Express. Theor. Appl. Genet. **116:** 797–806.

33. Lin, F., and X. M. Chen, 2009 Quantitative trait loci for non-race-specific, high-temperature adult-plant resistance to stripe rust in wheat cultivar Express. Theor. Appl. Genet. **118:** 631–642.

34. Liu, J., Z. Chang, X. Zhang, Z. Yang, X. Li *et al*., 2013 Putative Thinopyrum intermedium-derived stripe rust resistance gene *Yr50* maps on wheat chromosome arm 4BL. Theor. Appl. Genet. **126:** 265–274.

35. Liu, J., Z. He, L. Wu, B. Bai, W. Wen, *et al*., 2015 Genome-wide linkage mapping of QTL for adult-plant resistance to stripe rust in a Chinese wheat population Linmai 2 x Zhong 892. PLoS ONE **10:** e0145462.

36. Lowe, I., D. Cantu, and J. Dubcovsky, 2011 Durable resistance to the wheat rusts: integrating systems biology and traditional phenotype-based research methods to guide the deployment of resistance genes. Euphytica **179:** 69–79.

37. Lu, Y., C. Lan, S. Liang, X. Zhou, D. Liu *et al*., 2009 QTL mapping for adult-plant resistance to stripe rust in Italian common wheat cultivars Libellula and Strampelli. Theor. Appl. Genet. **119:** 1349–1359.

38. Lu, Y., M. Wang, X. Chen, D. See, S. Chao *et al*., 2014 Mapping of *Yr62* and a small-effect QTL for high-temperature adult-plant resistance to stripe rust in spring wheat PI 192252. Theor. Appl. Genet. **127:** 1449–1459.

39. Lukaszewski, A. J., 2000 Manipulation of the 1RS.1BL translocation in wheat by induced homoeologous recombination. Crop Sci. **40:** 216-225.

40. Ma, D., D. Y. Hou, M. Tang, H. Wang, Q. Li *et al*., 2013 Genetic analysis and molecular mapping of a stripe rust resistance gene *YrH9014* in wheat line H9014-14-4-6-1. J. Integr. Agric. **12:** 638–645.

41. Ma, J., R. Zhou, Y. Dong, L. Wang, X. Wang *et al*., 2001 Molecular mapping and detection of the yellow rust resistance gene *Yr26* in wheat transferred from *Triticum turgidum* L. using microsatellite markers. Euphytica **120:** 219–226.

42. Maccaferri, M., J. Zhang, P. Bulli, Z. Abate, S. Chao *et al*., 2015 A genome-wide association study of resistance to stripe rust (*Puccinia striiformis* f. sp. *tritici*) in a worldwide collection of hexaploid spring wheat (*Triticum aestivum* L.). G3 Genes Genom. Genet. **5:** 449–465.

43. Mallard, S., D. Gaudet, A. Aldeia, C. Abelard, A. L. Besnard *et al*., 2005 Genetic analysis of durable resistance to yellow rust in bread wheat. Theor. Appl. Genet. **110:** 1401–1409.

44. McGrann, G. R., P. H. Smith, C. Burt, G. R. Mateos, T. N. Chama *et al*., 2014 Genomic and genetic analysis of the wheat race-specific yellow rust resistance gene *Yr5*. J. Plant Sci. Mol. Breed. **3:** http://dx.doi.org/10.7243/2050-2389-3-2.

45. McIntosh, R. A., Y. Yamazaki, J. Dubcovsky, W. J. Rogers, C. F. Morris *et al*., 2013 Catalogue of gene symbols for wheat in 12th Int. Wheat Genet. Symp., edited by R.A. McIntosh, Yokohama, Japan

46. Melichar, J. P. E., S. Berry, C. Newell, R. MacCormack, and L. A. Boyd, 2008 QTL identification and microphenotype characterisation of the developmentally regulated yellow rust resistance in the UK wheat cultivar Guardian. Theor. Appl. Genet. **117:** 391–399.

47. Naruoka, Y., K. A. Garland-Campbell, and A. H. Carter, 2015 Genome-wide association mapping for stripe rust (*Puccinia striiformis* f. sp. *tritici*) in US Pacific Northwest winter wheat (*Triticum aestivum* L.). Theor. Appl. genet. **128:** 1083–110.

48. Prins, R., Z. A. Pretorius, C. M. Bender, and A. Lehmensiek, 2011 QTL mapping of stripe, leaf and stem rust resistance genes in a Kariega × Avocet S doubled haploid wheat population. Mol. Breed. **27:** 259–270.
49. Quan, W., G. Hou, J. Chen, Z. Du, F. Lin *et al*., 2013 Mapping of QTL lengthening the latent period of Puccinia striiformis in winter wheat at the tillering growth stage. Eur. J. Plant Pathol. **136:** 715–727.
50. Ramburan, V. P., Z. A. Pretorius, J. H. Louw, L. A. Boyd, P. H. Smith *et al*., 2004 A genetic analysis of adult plant resistance to stripe rust in the wheat cultivar Kariega. Theor. Appl. Genet. **108:** 1426–1433.
51. Randhawa, M., U. Bansal, M. Valárik, B. Klocová, J. Doležel *et al*., 2014 Molecular mapping of stripe rust resistance gene *Yr51* in chromosome 4AL of wheat. Theor. Appl. Genet. **127:** 317–324.
52. Ren, Y., Z. He, J. Li, M. Lillemo, L. Wu *et al*., 2012a QTL mapping of adult-plant resistance to stripe rust in a population derived from common wheat cultivars Naxos and Shanghai 3/Catbird. Theor. Appl. Genet. **125:** 1211–1221.
53. Ren, Y., Z. Li, Z. He, L. Wu, B. Bai *et al*., 2012b QTL mapping of adult-plant resistances to stripe rust and leaf rust in Chinese wheat cultivar Bainong 64. Theor. Appl. Genet. **125:** 1253–1262.
54. Ren, Y., L. S. Liu, Z. H. He, L. Wu, B. Bai *et al*., 2015 QTL mapping of adult-plant resistance to stripe rust in 'Lumai 21 x Jingshuang 16' wheat population. Plant Breeding **134:** 501–507.
55. Rosewarne, G. M., R. P. Singh, J. Huerta-Espino, S. A. Herrera-Foessel, K. L. Forrest *et al*., 2012 Analysis of leaf and stripe rust severities reveals pathotype changes and multiple minor QTLs associated with resistance in an Avocet × Pastor wheat population. Theor. Appl. Genet. **124:** 1283–1294.
56. Rosewarne, G. M., R. P. Singh, J. Huerta-Espino, and G. J. Rebetzke, 2008 Quantitative trait loci for slow-rusting resistance in wheat to leaf rust and stripe rust identified with multi-environment analysis. Theor. Appl. Genet. **116:** 1027–1034.

57. Santra, D. K., X. M. Chen, M. Santra, K. G. Campbell, and K. K. Kidwell, 2008 Identification and mapping QTL for high-temperature adult-plant resistance to stripe rust in winter wheat (*Triticum aestivum* L.) cultivar "Stephens." Theor. Appl. Genet. **117:** 793–802.

58. Singh, R. P., J. C. Nelson, and M. E. Sorrells, 2000 Mapping *Yr28* and other genes for resistance to stripe rust in wheat. Crop Sci. **40:** 1148–1155.

59. Singh, A., M. P. Pandey, A. K. Singh, R. E. Knox, K. Ammar *et al*., 2013 Identification and mapping of leaf, stem and stripe rust resistance quantitative trait loci and their interactions in durum wheat. Mol. Breed. **31:** 405–418.

60. Suenaga, K., R. P. Singh, J. Huerta-Espino, and H. M. William, 2003 Microsatellite markers for genes *Lr34/Yr18* and other quantitative trait loci for leaf rust and stripe rust resistance in bread wheat. Phytopathology **93:** 881–890.

61. Uauy, C., J. C. Brevis, X. Chen, I. Khan, L. Jackson *et al*., 2005 High-temperature adult-plant (HTAP) stripe rust resistance gene *Yr36* from *Triticum turgidum* ssp. *dicoccoides* is closely linked to the grain protein content locus *Gpc-B1*. Theor. Appl. Genet. **112:** 97–105.

62. Vazquez, M. D., C. J. Peterson, O. Riera-Lizarazu, X. Chen, A. Heesacker *et al*., 2012 Genetic analysis of adult plant, quantitative resistance to stripe rust in wheat cultivar "Stephens" in multi-environment trials. Theor. Appl. Genet. **124:** 1–11.

63. Vazquez, M. D., T. Zemetra, C. J. Peterson, X. M. Chen, A. Heesacker *et al*., 2015 Multi-location wheat stripe rust QTL analysis: genetic background and epistatic interactions. Theor. Appl. Genet. **128:** 1307-1218.

64. William, M., R. P. Singh, J. Huerta-Espino, S. O. Islas, and D. Hoisington, 2003 Molecular marker mapping of leaf rust resistance gene *Lr46* and its association with stripe rust resistance gene *Yr29* in wheat. Phytopathology **93:** 153–159.

65. William, H. M., R. P. Singh, J. Huerta-Espino, G. Palacios, and K. Suenaga, 2006 Characterization of genetic loci conferring adult plant resistance to leaf rust and stripe rust in spring wheat. Genome **49:** 977–990.

66. Xu, L. S., M. N. Wang, P. Cheng, Z. S. Kang, S. H. Hulbert *et al*., 2013 Molecular mapping of *Yr53*, a new gene for stripe rust resistance in durum wheat accession PI 480148 and its transfer to common wheat. Theor. Appl. Genet. **126:** 523–533.

67. Yang, E. N., G. M. Rosewarne, S. A. Herrera-Foessel, J. Huerta-Espino, Z. X. Tang *et al*., 2013 QTL analysis of the spring wheat “Chapio” identifies stable stripe rust resistance despite inter-continental genotype × environment interactions. Theor. Appl. Genet. **126:** 1721–1732.

68. Zegeye, H, A. Rasheed, F. Makdis, A. Badebo, and F. C. Ogbonnaya, 2014 Genome-wide association mapping for seedling and adult plant resistance to stripe rust in synthetic hexaploid wheat. PLoS ONE **9:** e105593.

69. Zhao, L., J. Feng, C.-Y. Zhang, X.-D. Xu, X.-M. Chen *et al*., 2012 The dissection and SSR mapping of a high-temperature adult-plant stripe rust resistance gene in American spring wheat cultivar Alturas. Eur. J. Plant Pathol. **134:** 281–288.

70. Zheng, J., Z. Yan, L. Zhao, S. Li, Z. Zhang *et al*., 2014 Molecular mapping of a stripe rust resistance gene in wheat line C51. J. Genet. **93:** 443–450.

71. Zhou, X. L., W. L. Wang, L. L. Wang, D. Y. Hou, J. X. Jing *et al*., 2011 Genetics and molecular mapping of genes for high-temperature resistance to stripe rust in wheat cultivar Xiaoyan 54. Theor. Appl. Genet. **123:** 431–438.

72. Zwart, R. S., J. P. Thompson, A. W. Milgate, U. K. Bansal, P. M. Williamson *et al*., 2010 QTL mapping of multiple foliar disease and root-lesion nematode resistances in wheat. Mol. Breed. **26:** 107–124.
