# Supplementary material for: Genetic Architecture of Resistance to Stripe Rust in a Global Winter Wheat Germplasm Collection
Source: G3 (Bethesda). 2016 May 25;6(8):2237–53. doi: 10.1534/g3.116.028407 (PMC4978880; doi:10.1534/g3.116.028407)
Supplement: Supplemental Material [file supp_g3.116.028407_FigureS1.pptx]

## Slide 1
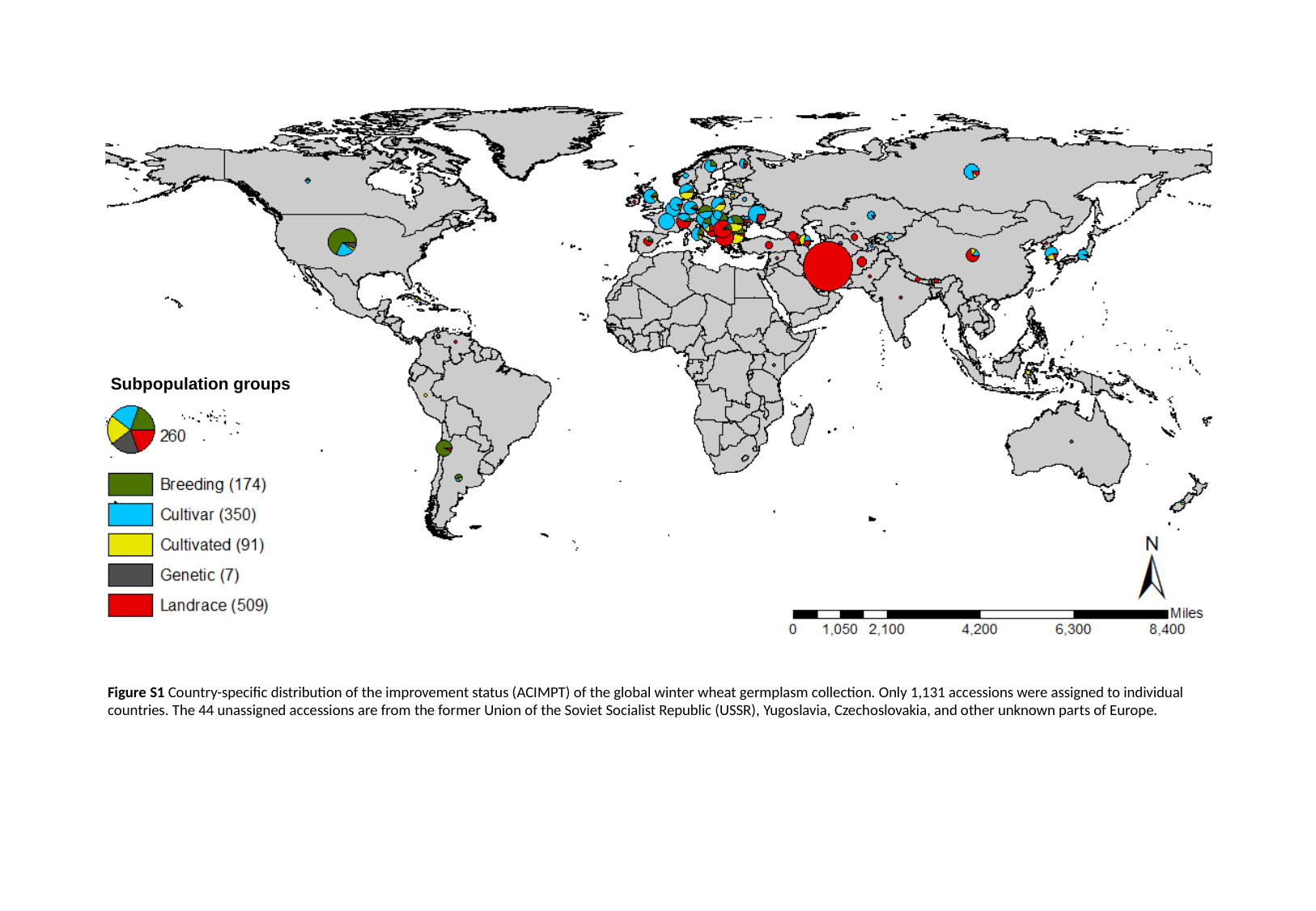

Subpopulation groups
Figure S1 Country-specific distribution of the improvement status (ACIMPT) of the global winter wheat germplasm collection. Only 1,131 accessions were assigned to individual countries. The 44 unassigned accessions are from the former Union of the Soviet Socialist Republic (USSR), Yugoslavia, Czechoslovakia, and other unknown parts of Europe.
