# Supplementary material for: Genetic Architecture of Resistance to Stripe Rust in a Global Winter Wheat Germplasm Collection
Source: G3 (Bethesda). 2016 May 25;6(8):2237–53. doi: 10.1534/g3.116.028407 (PMC4978880; doi:10.1534/g3.116.028407)
Supplement: Supplemental Material [file supp_g3.116.028407_FigureS2.pptx]

## Slide 1
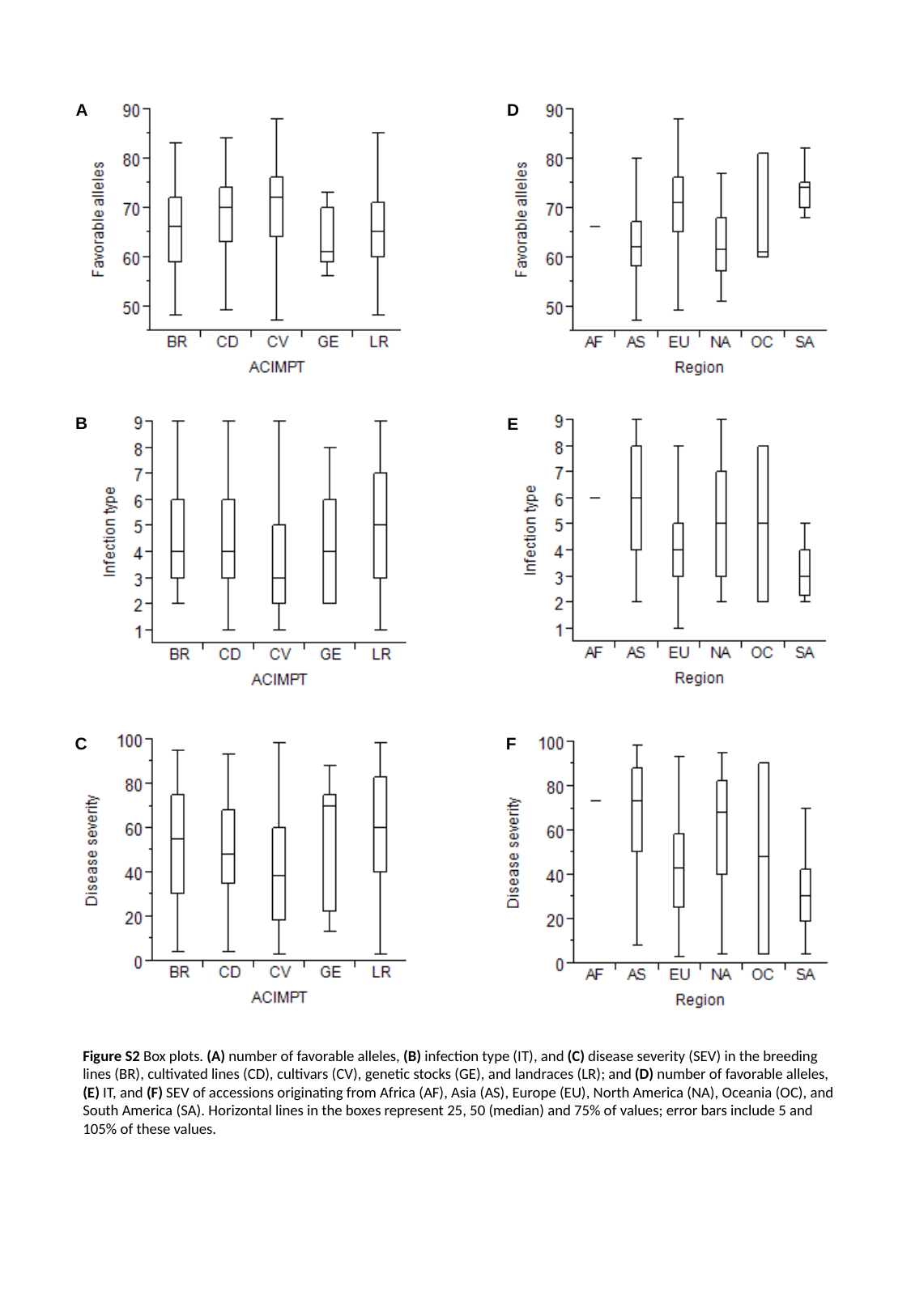

A
D
B
E
C
F
Figure S2 Box plots. (A) number of favorable alleles, (B) infection type (IT), and (C) disease severity (SEV) in the breeding lines (BR), cultivated lines (CD), cultivars (CV), genetic stocks (GE), and landraces (LR); and (D) number of favorable alleles, (E) IT, and (F) SEV of accessions originating from Africa (AF), Asia (AS), Europe (EU), North America (NA), Oceania (OC), and South America (SA). Horizontal lines in the boxes represent 25, 50 (median) and 75% of values; error bars include 5 and 105% of these values.
